
JOURNAL INFORMATION
==============================
NLM Title Abbreviation: JBMR Plus
Journal ID: JBMR Plus
NLM Journal ID: 101707013
Journal ID: jbmrplus

JBMR Plus

EISSN: 2473-4039
Publisher: Oxford University Press

ARTICLE INFORMATION
==============================
PMCID: PMC11327403
DOI: 10.1093/jbmrpl/ziae109
Article ID: ziae109
Article version: 1
Subjects: Abstracts, AcademicSubjects/MED00010, AcademicSubjects/MED00160, AcademicSubjects/MED00250

Abstracts of the Bone Research Society Annual Meeting 2024

Publication date: 2024 Aug
Electronic publication date: 2024 Aug 16
Volume: 8
Issue: Suppl 3
First page: iii1
Last page: iii130
Copyright: © The Author(s) 2024. Published by Oxford University Press on behalf of the American Society for Bone and Mineral Research.
Copyright year: 2024
License: This is an Open Access article distributed under the terms of the Creative Commons Attribution License (https://creativecommons.org/licenses/by/4.0/), which permits unrestricted reuse, distribution, and reproduction in any medium, provided the original work is properly cited.
License URL: https://creativecommons.org/licenses/by/4.0/

Page count: 1

==============================

Bone Research Society
ANNUAL MEETING 2024
10-12 JULY 2024 | SHEFFIELD, UK

### Book of Abstracts

### Sheffield, UK

### Wednesday 10 – Friday 12 July 2024

**Invited Speaker Talk Summaries**

**Clinical Studies Oral Communications**

**Oral Communications**

**Printed Poster Abstracts**

### Overview of the BRS Annual Meeting 2024

The Bone Research Society (BRS), formerly the Bone and Tooth Society, was founded in 1950. The BRS is one of the largest national scientific societies in Europe dedicated to clinical and basic research into mineralised tissues and is the oldest such society in the world. Meetings are held annually, attracting a wide audience from throughout the UK and beyond. The presentations are traditionally balanced between clinical and laboratory studies. The participation of young scientists and clinicians is actively encouraged.

We were delighted to welcome over 150 delegates, faculty and sponsors to our in-person meeting in Sheffield, which was held at The Edge, part of the University of Sheffield campus from Wednesday 10 to Friday 12 July 2024.

There were two days with a full programme of invited speakers, debate, oral communications, posters presenting and two satellite symposia from our industry partners. We would like to thank our 13 industry sponsors and the Bone Cancer Research Trust for their valuable support of our meeting.

The day before we also held our popular workshops; this year we added the specialised Image based BioMechanics Workshop and CT to Printed Models Workshop. These were followed by the popular Cancer and Bone Workshop, a new Single-cell Multiomics and Spatial Transcriptomics in Musculoskeletal Research session and then the Rare Bone Disease session. We were delighted to hold our increasingly well attended Early Career Researchers Workshop (formerly known as the New Investigators Workshop) on the Wednesday evening.

Topics covered were:

- Rare Bone Disease
- Muscle and Bone
- Novel Regulatory Mechanisms in Bone
- Osteoporosis and Bone Disorders
- Extracellular Vesicles
- Cancer and bone
- Musculoskeletal Tissue Cross Talk
- Next generation approaches for next generation therapies

We were delighted to have excellent submissions from our authors which resulted in 23 being selected for Oral Communication presentations, 25 more for Quick Fire Poster Pitches and we had 60 printed posters on display.

***BRS 2024 Local Organising Committee***
Professor Allie Gartland (Chair)
Dr Enrico Dall'Ara
Professor Richard Eastell
Dr Michelle Lawson
Professor Christine Le Maitre
Professor Graham Russell
Dr Karan Shah
Professor Mark Wilkinson

### Invited Speaker Talk Summaries

### CBW1.1

*No longer presenting at meeting*

### CBW1.2

### Models of metastatic prostate cancer and tumour dormancy

Dr Ning Wang (University of Leicester, Leicester, UK)

Prostate cancer is the most commonly diagnosed cancer and the second leading cause of cancer death in men in the UK. Bone is the most common site for metastasis in prostate cancer. Once clinically detectable bone metastases are established, the disease is considered incurable. Importantly, even after the primary tumour is diagnosed, metastatic lesions can remain indolent or 'dormant' and undetected for many years. Therefore, identifying key mechanisms associated with prostate cancer entering and existing dormancy is a major priority for stratifying patients at risk of developing bone metastasis, and preventing/treating the establishment of prostate cancer bone metastases.

In our previous studies, we used vital dyes that rapidly disappear as cells divide to track cells and, more importantly, to show when they have proliferated. Using this model, combined with multiphoton microscopy, we demonstrated that the cells that arrive and initially survive in bone are mitotically dormant. We also defined the distribution of these dormant tumour cells and the so-called bone metastasis niche in bone in vivo. This mitotically dormant phenotype exists at low frequency (<1% of the total population) in prostate cancer cell lines grown in vitro, and it is not a defined subset of the cell lines but rather a 'plastic' phenotype that can be re-derived from isolated rapidly growing populations.

Further multiomics studies have enabled us to identify new candidate molecules and pathways central to the development of metastatic bone disease in prostate cancer. These findings guide our research in finding potential therapeutic targets for this deadly disease.

### CBW1.3

### New treatment approaches using oncolytic viruses

Professor Munitta Muthana (University of Sheffield, Sheffield, UK)

The survival strategies of infectious organisms have inspired many therapeutics over the years. Indeed, the advent of cancer killing oncolytic viruses (OVs) exploits the uncontrolled replication of cancer cells for production of their progeny resulting in a cancer-targeting treatment that leaves healthy cells unharmed. Their success against inaccessible tumours (deep in the body), however, is highly variable due to inadequate tumour targeting following injection into the circulation. To overcome this, we have developed several Trojans for delivering OVs. These include cells (macrophages), extracellular vesicles, liposomes, nanohydrogels, silk fibroin and magnetic nanoparticles (MNPs) derived from magnetotactic bacteria. Here we describe the co-assembly of these nanobugs within Trojans and how this enables tumour targeting from the circulation whilst protecting the OV against neutralising antibodies and thereby enhancing viral replication within tumours. These platforms additionally enhanced the intratumoural recruitment of activated immune cells, induced tumour shrinkage, reduced metastasis and increased survival in mouse models of cancer by 50%. Exploiting the properties of versatile nanocarriers for active tumour targeting, rather than tropism of the virus, offers an exciting, novel approach for enhancing the efficacy of tumour immunotherapies like OV, for disseminated neoplasms. This is particularly pertinent for cancer types that are refractory to immunotherapies.

### RBD1.1

### Skeletal Dysplasia's - Clinical and Radiological Clues to Diagnosis?

Professor Mohnish Suri (Nottingham University Hospitals NHS Trust, Nottingham, UK)

The skeletal dysplasias are a diverse group of genetically determined, constitutional disorders of bone growth and development. These disorders are classified based on the responsible genes, mechanisms, clinical or radiological features. The nosology of the skeletal dysplasias was most recently revised in 2023 and it encompasses 771 conditions in 41 diagnostic groups caused by variants in 552 genes.

Variants in some genes can cause multiple skeletal dysplasias, with a spectrum ranging from antenatal onset, severe, often lethal, disorders to very mild, adult-onset disorders. Examples of this are the spectrum of disorders that can be caused by variants in FGFR3, COL2A1, COL11A1, FLNA, FLNB, and TRPV4. There are also many examples of the same skeletal dysplasia phenotype being caused by variants in multiple genes. Examples of this include Stickler syndrome, multiple epiphyseal dysplasia, osteogenesis imperfecta, severe early onset osteopetrosis, and hereditary multiple osteochondromas.

Diagnosis of skeletal dysplasias can be difficult and relies on the usual triad of history, with a particular emphasis on family history, including consanguinity, clinical examination findings, and radiological features from a set of x-rays called a skeletal survey, followed by genetic testing. In this talk, I will present a clinico-radiological approach to the diagnosis of skeletal dysplasias, highlighting some diagnostically helpful clinical and radiological findings.

Although genetic testing is available for most skeletal dysplasias, I will demonstrate the utility of a clinico-radiological approach for the diagnosis of skeletal dysplasias caused by pathogenic or likely pathogenic variants in the COL2A1 gene.

### RBD1.2

### Pre-clinical studies informing skeletal biology and new treatments for rare skeletal disorders

Professor Mike Briggs (Newcastle University, Newcastle, UK)

Genetic skeletal diseases are a clinically variable and genetically heterogeneous group of over 350 different phenotypes. Although individually rare, as a group of related diseases they have a combined incidence of 1/3,500 and there are currently very few treatment options available.

One approach to develop novel therapies is to identify common disease mechanisms, which are genotype and/or phenotype independent, and can be targeted therapeutically. In this context, we have unequivocally demonstrated that endoplasmic reticulum (ER) stress, induced in chondrocytes due to the retention of mutant cartilage structural proteins, is a shared mechanism in a broad group of phenotypes. Notably, ER-stress underpins the pathologies in diseases resulting from mutations in type X collagen, matrilin-3 and COMP, and a lesser extent aggrecan, type II and IX collagenopathies.

We have previously demonstrated that carbamazepine can reduce ER-stress, correct growth plate dysplasia and restore long bone growth in a mouse model of metaphyseal chondrodysplasia type Schmid (MCDS; type  X collagen). We are currently undertaking an open label multi-national clinical trial to determine the safety and efficacy of treating MCDS children with CBZ. In parallel to the clinical trial we are also identifying and validating relevant biomarkers that will aid in future treatment protocols.

Unfortunately, CBZ has no effect in cell and mouse models of conditions resulting from mutations in matrilin-3 and COMP (i.e. PSACH and MED). We have therefore developed an ER-stress luciferase-based assay to undertake high throughput screening of an FDA approved drug library. This screening will identify potential lead compounds that will then be assessed in greater detail in cell models of PSACH-MED, before selected compounds will eventually be tested in the relevant mouse models.

Targeting shared disease mechanisms, such as ER-stress, potentially through drug repurposing is a powerful and timely approach to identify new therapies for genetic skeletal diseases and rare disease in general.

### RBD1.3

### New era of drug repurposing for rare skeletal disorders and wrap up

Professor Nick Bishop (University of Sheffield, Sheffield, UK)

"Drug repurposing", often referred also to as "drug repositioning", means taking a drug that has a licence for one indication and using it for another. There are some 3000 drugs currently licenced and around 12000 known diseases; some diseases have more than one drug licenced for their treatment, so the "gap" is significant. One approach to closing the gap is that we use the drugs we already have, either alone or in combination, to target pathways that have been identified as critical to the as-yet untreated diseases. The paradigm that “all drugs have side-effects” provides the clue that “off-target” effects might actually be beneficial in treating some diseases.

Already active in this area are the repurposing consortia funded by Horizon Europe as well the NHS Medicines Repurposing Programme, the UKRI with their “repurposing toolkit”, and the EveryCure initiative in the US. As well as giving an over view of the landscape and challenges of repurposing, I will give a specific example in the area of rare bone disease, the use of losartan in osteogenesis imperfecta.

### S1.1

### Incretin hormones as regulators of bone turnover

Dr Morten Frost (University of Southern Denmark, Odense, Denmark)

Incretin hormones GIP and GLP-1 are secreted after food intake and promote insulin secretion and satiety, and incretin hormone analogues are used clinically to treat type 2 diabetes and obesity. Moreover, GIP and GLP-1 are the main regulators of the acute meal-associated changes in bone turnover. The talk will cover preclinical and clinical data on the effects of incretin hormones and their analogues on bone turnover, bone mass, and fracture risk.

### S1.2

### Regulation of phosphate metabolism and its interaction with iron metabolism

Dr Tatiane Vilaca (University of Sheffield, Sheffield, UK)

Phosphate is essential for life, playing a fundamental role in bone mineralization, cell signalling, the synthesis of lipids and nucleic acids, and energy metabolism. The homeostatic control of phosphate concentrations is dependent on intricate endocrine feedback processes that involve the parathyroids, intestines, kidneys, and bones.

Parathyroid hormone (PTH), vitamin D, and fibroblast growth factor 23 (FGF23) are key regulators in maintaining phosphate homeostasis. PTH stimulates bone resorption, releasing phosphate and calcium into the bloodstream while enhancing renal phosphate excretion. Vitamin D promotes intestinal phosphate absorption. FGF23, primarily produced by osteocytes, acts on the kidneys to decrease phosphate reabsorption and inhibit vitamin D activation, promoting phosphaturia and decreasing serum phosphate levels.

Research findings suggest that iron deficiency, acute blood loss, and inflammation act as potent stimulators of FGF-23 production and cleavage. Moreover, recent evidence indicates that FGF23 fragments have a physiological function in iron metabolism. These findings show a two-way connection between phosphate and iron metabolism. Exploring these interactions can help to elucidate the pathophysiology of disorders involving mineral and iron imbalances and provide potential insights for therapeutic interventions.

### S2.1

### Bone mineral density as a surrogate for fracture risk reduction in osteoporosis clinical trials

Dr Marian Schini (University of Sheffield, Sheffield, UK)

Fragility fractures related to osteoporosis, lead to high morbidity and increased mortality, and are a large and growing public health problem.  Currently one in two women and one in five men over age 50 will suffer a fracture in their remaining lifetime.  Despite availability of several FDA-approved therapies that reduce the risk of fractures, there an urgent need for new osteoporosis medications. At the moment, clinical trials use fracture endpoints, resulting in a large number of participants required, with long follow up periods, increasing the cost substantially.

The SABRE study is an initiative funded by the Foundation for the National Institutes of Health (FNIH) and the American Society for Bone and Mineral Research (ASBMR) to advance bone mineral density (BMD) as a regulatory endpoint for osteoporosis drugs. The SABRE study has assembled the largest dataset of individual patient data from randomised, placebo-controlled trials of osteoporosis through a public-private partnership.

This unique dataset allowed us to test whether changes in BMD would be useful as a surrogate outcome for fractures in future clinical trials of new osteoporosis drugs. The use of a surrogate marker will reduce the need for large, costly studies for drug approval.

We have progressed through the FDA qualification process by submitting a Full Qualification Package in January 2024. If this is approved, it will promote the development of new drugs and increase the treatment options for patients.

### S2.2

### Heterotopic ossification

Professor Liam Grover (University of Birmingham, Birmingham, UK)

The pathological formation of bone in soft tissues following traumatic injury remains relatively poorly understood.  Numerous, plausible mechanisms have been proposed, each leading to the development of novel therapeutic options, however, there is still no consensus as to the precise aetiology of the condition.  Over the past ten years we have explored the structure and chemistry of pathological ossification and compared the tissue with non-pathological bone.  This work has shown structural similarities of this tissue to normal bone, but some interesting compositional variations, identifying potential differences in the mechanism of mineralisation and targets for the development of therapeutics.  In parallel with this, with collaborators, we have developed new culture systems to allow for high-throughput screening of therapeutic agents and have taken one of these candidates into an in vivo model of the condition.  Recent work reporting a neuroinflammatory component of HO suggests alternative approaches to locally inhibit bone formation in high-risk patients.

### S3.1

### Understanding the origin and prospective therapeutic application of pro-osteogenic extracellular vesicles

Dr Owen Davies (Loughborough University, Loughborough, UK)

Dr Davies' talk will focus on the application of extracellular vesicles (EVs) for regenerative applications in bone. During the talk he will discuss the importance of EV source, distinguishing between matrix-bound and un-bound vesicles. The talk will also propose prospective markers required for EVs to drive mineralisation. Dr Davies will demonstrate how advanced multi-disciplinary techniques can be applied to characterise variations in EV composition at the single particle level and how an advanced understanding of EV composition is essential for driving future translation.

### S3.2

### Extracellular vesicles analysis in liquid biopsies

Professor An Hendrix (Ghent University, Ghent, Belgium)

Research into extracellular vesicles (EVs) has yielded important biological insights and raised the prospect of developing novel biomarkers for cancer detection and monitoring (Hendrix, Nat Rev Mol Cell Biol, 2021). As with other emerging and transformative fields in research, it requires a broad, supportive base for EV research to mature into clinical application (Hendrix et al., Nat Rev Meth Prim, 2023). My lab developed protocols, reference materials and reporting tools to enable biomarker discovery and validation in liquid biopsies (Van Deun et al., Nat Methods, 2017; Geeurickx et al., Nat Comm, 2019; Tulkens et al., Gut, 2020; Vergauwen et al., JEV, 2021, Dhondt et al., JEV, 2023).

### S4.1

### Fluorescence guided surgery for benign and malignant bone tumours - intraoperative and histopathological findings

Dr Kenneth Rankin (Newcastle University, Newcastle, UK)

Background: Fluorescence guided surgery (FGS) using indocyanine green (ICG) has emerged as an effective technology to guide surgery for a range of indications over the last 15 to 20 years including the assessment of tissue perfusion and lymph node mapping. More recently, the concept of tumour margin identification has gained traction and in our institution we have demonstrated that FGS can be effective for curettage of benign bone tumours and bone metastases and to assist with the marginal or wide excision of primary bone sarcomas and large bone metastases.

Methodology: ICG is administered intravenously at a dose of 1mg/kg on the day prior to surgery for the excision of primary bone sarcomas. For cases undergoing curettage, intravenous ICG can be administered on induction of anaesthesia. Fluorescence is assessed with an infrared camera designed for use in open surgery. Postoperatively, the tumour specimens are processed in a standard manner into FFPE blocks and a pathology sub-study has demonstrated that the spatial orientation of ICG at the tumour margins can be analysed using standard fluorescence microscopy and also fluorescence lifetime imaging.

Results: We have obtained funding from the National Institute of Health and Care Research to run the SarcoSIGHT randomised controlled trial which is open in the UK to recruit patients with bone and soft tissue sarcomas to assess the effect of FGS on reduction of the positive margins rates. This trial includes fluorescence mapping and pathology sub-studies which will form the cornerstone for future projects.

### S4.2

### Unravelling the differential antitumor effects of adjuvant bisphosphonates in pre- and post-menopausal breast cancer patients

Professor Penelope Ottewell (University of Sheffield, Sheffield, UK)

The development of targeted treatments for breast cancer has led to increased survival for patients whose tumours are retained in the primary site. However, no curative treatments are available for those whose tumours have metastasised to distal organs. Bone is the most prevalent site for breast cancer metastases followed by lung, liver and brain. A large number of preclinical studies indicated that combining bisphosphonates with standard of care therapies would be effective at reducing bone metastases as well as tumour growth in soft tissues. Clinical trials including AZURE and ABCSG-12 confirmed that adding adjuvant bisphosphonates to standard of care reduced incidences of bone and soft tissue metastasis improving survival in postmenopausal women. However, in premenopausal women although adjuvant bisphosphonates reduced bone metastasis no increased survival was observed and, in some studies, pre-menopausal women experienced worse outcome due to increased soft tissue metastases. In this talk I will discuss the aforementioned data before focusing on new laboratory-based investigations that are providing novel insights into the reasons for differential anti-tumour effects of bisphosphonates in pre-and postmenopausal women with breast cancer. Finally, we will discuss potential methods for stimulating the anti-tumour actions of bisphosphonates for this young population.

### S5.1

### Cells are not alone - the importance of maintaining tissue crosstalk in ex vivo culture models

Professor Christine Le Maitre (University of Sheffield, Sheffield, UK)

Musculoskeletal tissues including bone, cartilage and disc are composed of dense extracellular matrix which is deposited and maintained by a low density of cells. However during the majority of laboratory studies these cells are issolated from the dense extracellular matrix environment and cultured within non-physiological systems in vitro oftern as issolated cell cultures resulting in their de-differentiation and behaviour which does not mimic that of the in vivo environment. This presentation will discuss alternative tissue explant culture systems which can be deployed to investigate cell function and in particular response to mechanical load where it is essential to maintain cell:ECM cross talk. Importantly these culture systems also enable cell to cell cross talk within bone explants and with adjacent tissues such as cartilage in osteochondral explants and the intervertebral disc in disc organ cultures which have been designed to retain boney end plates ensuring maintance of tissue cross talk. The potential of such systems will be discussed.

### S5.2

### What happens in the joint when inflammation meets damage

Professor Christopher Buckley (The Kennedy Institute of Rheumatology, Oxford, Uk)

The synovium is a thin mesenchymal membrane encapsulating the joint space and is the major site of pathology in rheumatoid arthritis. Synovial fibroblasts comprise a key cell type in the hyperplastic pannus that invades and destroys cartilage and bone via their production of matrix degrading enzymes [1]. However, they are also major contributors to inflammation by providing an amplificatory loop that drives the production of cytokines such as IL6 [1-2] Emerging data now suggests that they also play a role in dictating stromal memory (the tendency for arthritis to flare at the same set of joints) [3] and mediating chronic pain via the production of nerve growth factors [4].

Until now, it has remained unclear if all these functions are mediated by one, or several different subsets of fibroblasts as functional subclasses of fibroblasts have proven difficult to define, characterize and study in health and disease. In contrast the identification of leucocyte subsets with non-overlapping effector functions provided a molecular framework for the development of targeted therapies that have demonstrated spectacular success in immune-mediated inflammatory diseases.

In this lecture I will explain the interrelationships between synovial fibroblast subsets in the synovium and show how distinct fibroblast subsets drive inflammation and damage in arthritis

I will describe the functional relationships between alterations in fibroblast subsets and disease outcome during the development of human arthritis. Finally, I will speculate that distinct subsets of synovial fibroblasts are responsible for tissue inflammation, damage, memory, and pain.

### S6.1

### Optimising diagnoses of patients with genetic diseases

Professor Matt Brown (Genomics England, London, UK)

Over the past 15 years, the diagnosis of genetic conditions including skeletal disorders has been revolutionized by the advent of next-generation sequencing and novel analytic approaches. These advances have enabled the diagnosis of individual patients often without the need for testing relatives or performing functional assays. What began as a research-only tool has rapidly evolved into a routine clinical service within genomic medical services. However, despite these advances, only about 25-30% of patients referred for diagnostic testing receive a definitive genetic diagnosis.

Increasing this diagnostic rate is crucial to reducing the prolonged diagnostic odyssey faced by these patients and to providing effective treatments. Current efforts to improve diagnostic rates are focusing on better 'omics-enabled profiling and enhanced analytic methods. New 'omics technologies, including long-read and epigenetic profiling, transcriptomics and proteomics (including bulk, single-cell, and spatial approaches), and metabolomics, offer far more comprehensive insights into the biological processes underlying genetic diseases. Although considerable development and research are needed to optimize the use of these technologies and identify their particular clinical niches, early examples indicate significant potential benefits.

Research into analytic methods is also advancing, with efforts including telomere-to-telomere genetics, AI interpretation of variants of uncertain significance, and multivariate analysis techniques. Furthermore, the expansion of population and disease-related data in non-European ancestries is enhancing genomic diagnostic performance across diverse populations.

In conclusion, while significant progress has been made in diagnosing genetic disorders, there is a lot of scope for improvement. By continuing to refine these approaches, we can improve diagnostic rates, reduce the diagnostic odyssey for patients, and pave the way for more effective treatments.

### S6.2

### Precision Medicine in Bone and Soft Tissue Sarcomas

Dr Paul Huang (Institute of Cancer Research, London, UK)

Bone and soft tissue sarcomas are a group of rare and heterogeneous cancers that are challenging to treat. Therapeutic advances for these diseases have lagged behind many of the more common cancer types. My talk will discuss the use of various Omics based approaches to develop predictive and prognostic tools to better diagnose and treat patients with these diseases. I will describe our efforts to use cutting edge proteomics based strategies to define new biological ways to classify sarcomas and identify new targets for drug discovery and clinical trials.

### Clinical Studies Oral Communications

### CS1

### Evaluating the efficacy and safety of Setrusumab for osteogenesis imperfecta: Six-month phase 2 data from the Phase 2/3 Orbit Study

Luigi A Picaro[1], Gary Gottesman[2], Thomas O Carpenter[3], Maegen Wallace[4], Peter Smith[5], Erik A Imel[6], Huy Wang[7], Heather M Byers[7], Stanley Krolczyk[7], Michael Lewiecki[8]

[1]Mereo Biopharma Ltd, London, UK. [2]Washinton University Schools, St Louis, USA [3]Yale University School Of Medicine, New Haven, USA [4]Phoenix Children's Hospital, Phoenix, USA [5]Shriner's Hospital For Children, Chicago, USA [6]Indiana University School Of Medicine, Indianapolis, USA [7]Ultragenyx Pharmaceuticals Inc, Novato, USA [8]University Of New Mexico Health Science Center, Albuquerque, USA

### Abstract

Osteogenesis imperfecta (OI) is a rare genetic disorder characterized by low bone mass and fragility with no universally accepted treatment. In a prior Phase 2 study, setrusumab, a fully human anti-sclerostin monoclonal antibody, improved bone mineral density (BMD), strength, and turnover markers in adults with OI. In the Phase 2/3 Orbit study (NCT05125809), Phase 2 evaluated setrusumab in children and young-adults with OI based on PK/PD, safety, and BMD data, to determine the dosing strategy for Phase 3. Subjects with OI Types I, III, or IV, ages 5-<26 years, and having fractures within the prior 2 years randomized 1:1 to receive 20 or 40 mg/kg setrusumab intravenously monthly. Six-month data from August 2023 are presented.

Twenty-four subjects (50% female, 75% <18 years of age) with OI Type I (n=17/24, 71%) or III/IV (n=7/24, 29%) were enrolled and randomized to receive setrusumab 20 mg/kg (n=14/24) or 40 mg/kg (n=10/24).

The mean (SD) baseline-corrected area under the effect curve for serum P1NP for 20 mg/kg setrusumab was 4153 (4407) µg/(L*day) over M1 of treatment, and 5256 (5521) µg/(L*day) for 40 mg/kg.

Mean (SE) spine BMD change from baseline to M3 and M6 was 9.1% (1.8%) and 12.8% (2.5%), respectively (all p<0.05 vs baseline) for setrusumab 20 mg/kg and 9.3% (2.4%) and 16.1% (3.9%) for 40 mg/kg (M6 p<0.05 vs baseline). Spine BMD Z-score [baseline -2.1 (0.8)] increased to -1.5 (0.8) at M3 and -1.3 (0.8) at M6 with setrusumab 20 mg/kg; after 40 mg/kg, spine BMD Z-score [baseline -1.1 (0.4)] increased to -0.6 (0.4) at M3 and -0.2 (0.4) at M6 (all p<0.05 vs baseline).

Early M6 outcomes show the median annualized fracture rate reduced significantly from 0.7 to 0 (p=0.042) after setrusumab initiation (calculated reduction: 67%). Fingers, toes, and craniofacial fractures excluded. 4/24 (17%) had new radiographically-confirmed fractures reported (precipitating events: one subject: slipped on ice, stubbed toe, one each: fell off tricycle, bending over, tripped/fell).

No unexpected adverse events were reported. Treatment-related adverse events included infusion-related reaction (7/24, 29%), headache (3/24, 13%) infusion site pain, bone pain, and upper respiratory tract infection (each 1/24, 4%).

In Orbit Phase 2, we observed significant increases from baseline in lumbar spine BMD at M3 and M6 for 20 and 40 mg/kg doses, with no differences between groups. Preliminary data suggests decreased fracture rates after setrusumab initiation.

### CS2

### Following hip fracture, hospital organisational factors associated with prescription of anti-osteoporosis medication on discharge, to address imminent refracture risk: Findings from the REDUCE record-linkage cohort study in England and Wales

Rita Patel[1], Andrew Judge[1], Antony Johansen[2], Muhammad K Javaid[3], Xavier Griffin[4], Tim Chesser[5], Jill Griffin[6], Elsa Marques[1], Yoav Ben-Shlomo[1], Celia Gregson[1]

[1]University of Bristol, Bristol, UK [2]Cardiff University and University Hospital of Wales, Cardiff, UK [3]University of Oxford, Oxford, UK [4]Queen Mary University of London, London, UK [5]North Bristol NHS Trust, Bristol, UK [6]Royal Osteoporosis Society, Bath, UK

### Abstract

Patients who sustain a hip fracture are known to be at imminent refracture risk. Their complex multidisciplinary rehabilitation needs to include falls prevention and anti-osteoporosis medication (AOM) to prevent such fractures. This study aimed to determine which hospital-level organisational factors predict prescription of post-hip fracture AOM, and refracture risk.

A cohort of 178,757 patients aged ≥60 years who sustained a hip fracture in England and Wales over 3 years (2016-19) was examined and followed for 1 year. Patient-level hospital admission datasets from 172 hospitals, the National Hip Fracture Database, and Office of National Statistics mortality data were linked to 71 metrics extracted from 18 hospital-level organisational reports. Multilevel models determined organisational factors, independent of patient case-mix, associated with (i) AOM prescription, (ii) refracture (by ICD10 coding).

Patients were mean (SD) 82.7 (8.6) years old, 71% female, with 18% admitted from care homes. Overall, 101,735 (57%) were prescribed AOM during admission; while 50,354 (28%) died during 1-year of follow-up, and 12,240 (7%) refractured. Twelve organisational factors were associated with AOM prescription, e.g., orthogeriatrician-led care compared to traditional care models (OR 4.65 [95%CI: 2.25-9.59]), and AOM was 9% (95%CI: 6%-13%) more likely to be prescribed in hospitals providing routine bone health assessment to all patients.

Refracture occurred at median 126 days (IQR 59-234). Eight organisational factors were associated with refracture risk; hospitals providing orthogeriatrician assessment to all patients within 72-hours of admission had an 18% (95%CI: 2-31%) lower refracture risk, those providing weekend physiotherapy an 8% (95%CI: 3-14%) lower risk, and where occupational therapists attended clinical governance meetings, a 7% (95%CI: 2-12%) lower risk. Delays initiating post-discharge community rehabilitation were associated with a 15% (95%CI: 3-29%) greater refracture risk.

This novel, national study highlights the importance of orthogeriatrician, physiotherapist and occupational therapist involvement in secondary fracture prevention in frail hip fracture populations. Fracture risk reductions associated with this multidisciplinary care provision are apparent within 12 months of hip fracture; notably longer timeframes may be needed to see such fracture risk reductions from some anti-osteoporosis treatments.

### CS3

### Hip shape has a causal effect on hip fracture but not hip osteoarthritis: Findings for a GWAS meta-analysis and Mendelian randomisation study

Benjamin Faber[1], Monika Frysz[1], Jiayi Zheng[2], Huandong Lin[3], Kaitlyn Flynn[4], Raja Ebsim[5], Fiona Saunders[6], Rhona Beynon[1], Jenny Gregory[1], Richard Aspden[6], Nicholas Harvey[7], Claudia Lindner[5], Tim Cootes[5], George Davey Smith[1], David Evans[4], Xin Gao[8], Sijia Wang[2], John Kemp[4], Jon Tobias[1]

[1]University of Bristol, Bristol, UK [2]Shanghai Institute of Nutrition and Health, University of Chinese Academy of Sciences, Shanghai, China [3]Fudan University, Shanghai, UK [4]The University of Queensland, Brisbane, Australia [5]The University of Manchester, Manchester, UK [6]University of Aberdeen, Aberdeen, UK [7]University of Southampton, Southampton, UK [8]Chinese Academy of Sciences, Shanghai, China

### Abstract

### Purpose

Hip shape is thought to be an important causal risk factor for hip osteoarthritis and hip fracture. However, observational studies on which these causal assumptions are based are liable to confounding. Mendelian randomisation uses genetic instrumental variables obtained from genome wide association studies (GWAS) to estimate causal effects. In this study we aimed to conduct GWAS meta-analyses for hip shape, and use the resultant single nucleotide polymorphisms (SNPs) to provide genetic instruments for hip shape in Mendelian randomisation analyses to examine whether hip shape has a causal effect on the development hip osteoarthritis and hip fracture.

### Methods

Statistical hip shape modelling was used to derive 10 hip shape modes (HSMs) from dual-energy X-ray absorptiometry (DXA) images in UK Biobank and Shanghai Changfeng cohorts (n=43,485, mean age 63 years old). Random-effects GWAS meta-analyses were conducted for each HSM (genome wide significance $P<5x10^{-8}$). Two sample Mendelian randomisation was used to estimate causal effects between each HSM and hip osteoarthritis and fracture. The primary analysis employed the inverse variance weighted (IVW) method. Sensitivity analyses, which included MR-Egger, simple mode, weighted median and mode approaches, were also conducted.

### Results

Analysis of the first 10 HSMs, which explained 86.5% of hip shape variance, identified 290 independent association signals ($P<5x10^{-8}$). Mendelian randomisation analyses provided no evidence of a causal relationship between any HSM and hip osteoarthritis (Figure 1). Interestingly, the reverse direction analyses showed some evidence of a causal effect of a genetic predisposition to hip osteoarthritis on hip shape, as described by HSM3 (cam-type femoral head with bulging of the lateral aspect) ($\beta_{IVW}$ 1.37 [95% CI 0.54-2.20], P $1.21x10^{-3}$). In contrast, HSM2 (higher neck-shaft angle) and HSM4 (wider femoral neck) appeared to have causal effects on hip fracture ($OR_{IVW}$ 1.27 [95% CI 1.12-1.44], P $1.79x10^{-4}$ and $OR_{IVW}$ 0.74 [0.65-0.84], P $7.60x10^{-6}$ respectively, Figure 1). Sensitivity analyses supported these findings.

**Conclusions**

We report the largest hip shape GWAS meta-analysis to date, that identified 171 novel hip shape loci. Mendelian randomisation analyses suggest hip shape may not cause hip osteoarthritis within the general population, rather that altered cam-type hip shape is a consequence of genetic predisposition to hip osteoarthritis. In contrast, causal relationships were observed between hip shape and hip fracture, supporting the utility of our hip shape genetic instruments.

Fig 1. Bi-directional causal analyses between hip shape, and hip osteoarthritis and hip fracture

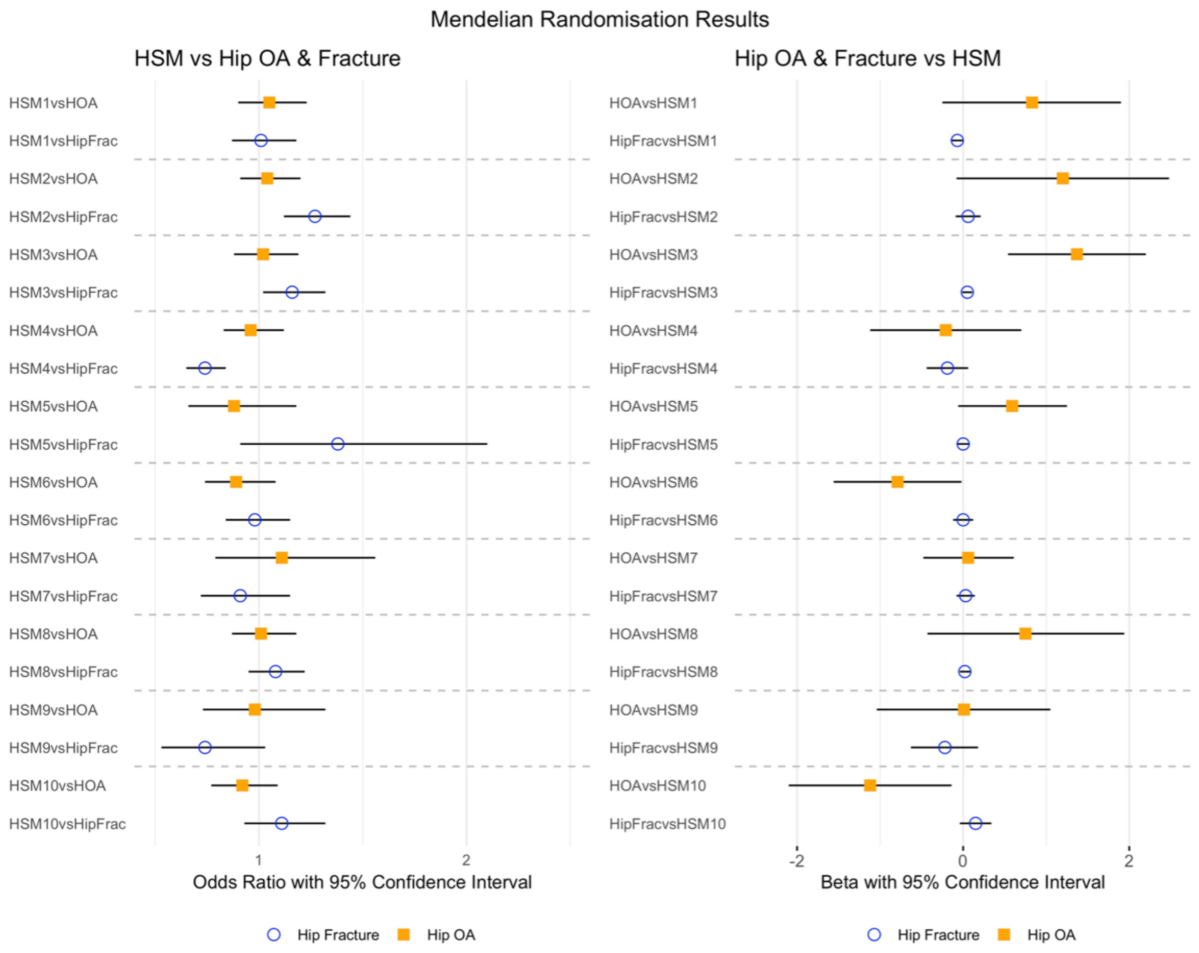

### CS4

### Camurati-Engelmann disease: A novel TGFB1 variant causing high bone mass with high bone turnover

Neelam Hassan[1], Celia Gregson[1], Emma Duncan[2], Jon Tobias[1]

[1]University of Bristol, Bristol, UK [2]King's College London, London, UK

### Abstract

We report a diagnostically challenging case of a patient with Camurati-Engelmann disease (CED) associated with a novel *TGFB1* variant.

A 34 year old male presented to clinic in 2014 with bone pain and hearing loss. He had a history of joint and limb pain and generalised muscle weakness from adolescence. He sustained a series of fractures through falls between the ages of 2 and 21. He developed bilateral hearing loss in his 20s requiring the use of hearing aids. There was no family history of note.

On examination, he was noted to be thin with reduced proximal muscle mass. He had shortened great toes, restricted range of movement in his hips and lumbar spine and bilateral sensorineural hearing loss. Examination was otherwise normal.

Investigations revealed a mildly elevated alkaline phosphatase (ALP 155U/L) with normal adjusted calcium, phosphate, parathyroid hormone and vitamin D. Procollagen 1 N-terminal propeptide (P1NP 179µg/L; reference range 20-76µg/L) and serum cross-linked C-telopeptide of type I collagen (CTX 1.7µg/L; 0.1-0.5µg/L) were significantly elevated. Radiographs of hands, hips, knees and feet demonstrated diffuse coarsened trabeculae and marked cortical thickening. DXA showed significantly increased bone mineral density (Table 1).

He was diagnosed with juvenile Paget's disease with a decision made to monitor his symptoms. The patient presented again in 2020 with worsening bone pain and raised ALP (411U/L). Alendronate resulted in almost complete resolution of his musculoskeletal symptoms and partial suppression of CTX (1.03µg/L) whereas zolendronate caused worsening bone pain and an increase in CTX (1.27µg/L). He was switched back to alendronate which provided effective symptomatic relief.

In 2024, he presented with unsteadiness and fatigue alongside worsening hearing loss. CT head revealed diffuse skull sclerotic thickening with narrowing of the bony internal auditory meati.

A skeletal dysplasia genetic panel did not reveal any causative variants; however a limited gene agnostic analysis of whole genome sequence data revealed a c.31_48 inframe duplication in *TGFB1* on chromosome 19q13. Protein modelling was conducted using AlphaFold2 demonstrating a 6 residue duplication (p.Leu11_Leu16dup) at the N-terminal of the transforming growth factor beta-1 (TGFβ1) proprotein (Figure 1). Activating mutations in TGFβ1 are known to cause CED, a rare autosomal dominant skeletal dysplasia, characterised by symmetrical cortical diaphyseal hyperostosis. Further studies are underway to investigate how this variant may affect protein function.

**Table 1**

| Site | T-score |
|---|---|
| Lumbar spine (L1-L4) | 4.1 |
| Right total hip | 3.0 |
| Right femoral neck | 5.3 |
| Left total hip | 2.8 |
| Left femoral neck | 5.3 |

**Figure 1: Protein model of the wild type TGFβ1 homodimer (in green) aligned with the mutant TGFβ1 homodimer (in blue). The p.Leu11_Leu16 duplication is highlighted in magenta at the N-terminal of the mutant homodimer.**

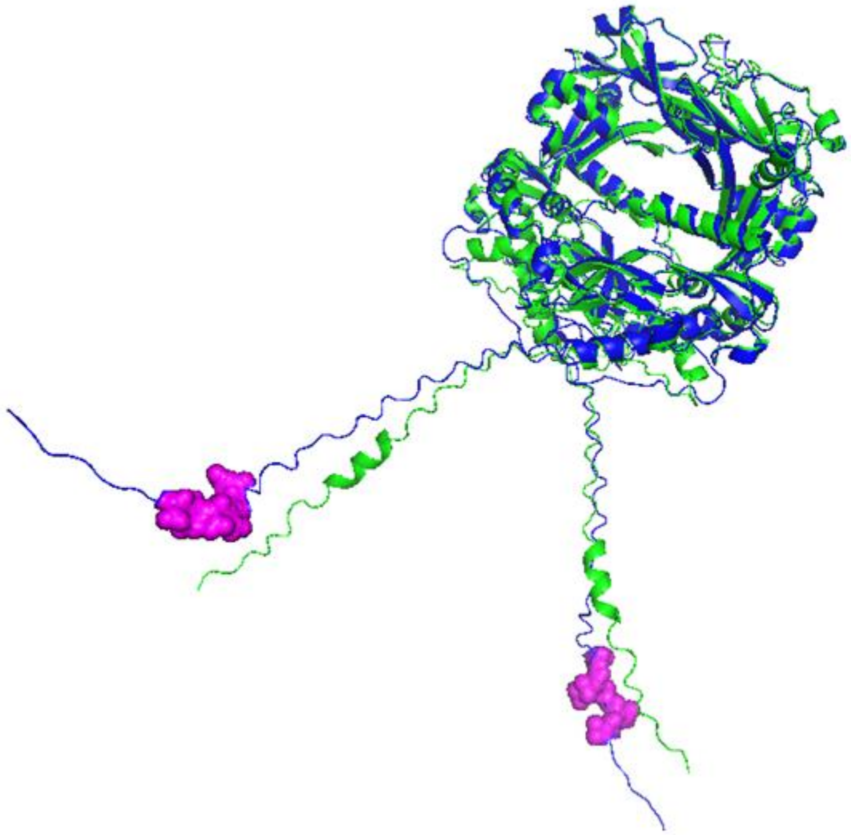

### CS5

### Identification of a rare heterozygous variant in WDR5 associated with high bone mass

Neelam Hassan[1], Marc van der Kamp[1], Celia Gregson[1], Emma Duncan[2], Jon Tobias[1]

[1]University of Bristol, Bristol, UK [2]King's College London, London, UK

### Abstract

### Introduction

This study aimed to identify novel monogenic causes of unexplained high bone mass (HBM).

### Methods

All participants in the UK HBM study (355 HBM cases, 200 unaffected relatives) underwent clinical assessment and DXA scanning. HBM was defined as a Z-score of ≥+3.2 at the total hip (TH) or first lumbar vertebra ($L_1$) (index cases) or a summed Z-score (TH + $L_1$) of ≥+3.2 (relatives). Whole exome sequencing (WES) was undertaken in 23 UK HBM pedigrees. Data were analysed for carriage of novel or rare (MAF<0.005) nonsynonymous single nucleotide variants or indels in highly conserved gene regions, segregating with HBM. Variants were filtered by functional prediction using SIFT, Polyphen-2, PMut and Mutation Taster. WES data from 493 low bone mass cases in the Anglo-Australasian Osteoporosis Genetics Consortium (hip Z-Score <-1.5) were analysed to ensure absence of variants. Protein homology modelling was conducted using the SwissModel server with visualisation in PyMOL.

### Results

A rare (MAF 0.000008) heterozygous missense variant in *WDR5* (GRCh37/hg19 NM_017588.3:c.854T>C, p.Ile285Thr) segregated with HBM in five affected family members (four affected siblings and child of one affected person; probability of random segregation with affection status = 0.03125). Clinical and biochemical characteristics are described in Table 1. All five family members had HBM with no history of fracture. Four reported difficulty floating when swimming and two had tori. P1NP was mildly suppressed in two individuals but other markers of bone turnover were normal.

*WDR5* encodes a highly conserved nuclear protein which forms a core scaffolding component of several multiprotein complexes involved in histone modification. There are limited data available from animal models regarding its role in bone but previous studies have indicated that WDR5 regulates osteoblast differentiation.

WDR5 has two major binding interfaces: the WDR5 interaction motif (WIN) and the WDR5-binding motif (WBM) on opposite sides of its barrel-shaped structure. Protein modelling showed that p.Ile285 is located in a hydrophobic pocket, close to the WBM site (Figure 1). Substituting hydrophobic isoleucine with hydrophilic threonine is predicted to cause instability, indirectly affecting substrate binding to the WBM site.

### Conclusion

Variants in *WDR5* may cause autosomal dominant HBM through effects on the WBM site.

**Table 1**

| | HBM pedigree, WDR5 c.854T>C p.Ile285Thr | | | | |
|---|---|---|---|---|---|
| | **P1** | **P2** | **P3** | **P4** | **P5** |
| Age | 76 | 74 | 70 | 68 | 43 |
| Sex | Male | Female | Male | Female | Female |
| Height (cm) | 171 | 164 | 173 | 158 | 165 |
| Weight (kg) | 96 | 80 | 96 | 76 | 78 |
| BMI ($kg/m^2$) | 33 | 30 | 32 | 30 | 29 |
| Adult fracture | No | No | No | No | No |
| Torus | No | Yes | No | No | Yes |
| Nerve compression | No | No | No | No | No |
| Broad frame | Yes | Yes | Yes | Yes | No |
| Buoyant when swimming | Yes | No | No | No | No |
| **DXA measurements** | | | | | |
| $L_1$ Z-score | +5.2 | +3.5 | +3.0 | +5.5 | +1.7 |
| TH Z-score | +2.3 | +3.4 | +2.2 | +1.3 | +1.8 |
| Bone mineral content (kg) | 3.95 | 3.20 | 3.86 | 2.63 | 2.92 |
| Fat mass (kg) | 32 | 42 | 28 | 31 | 33 |
| Lean mass (kg) | 59 | 47 | 63 | 41 | 41 |
| **Blood tests**[a] | | | | | |
| Adjusted calcium (mmol/L) | 2.4 | 2.54 | 2.47 | 2.47 | 2.4 |
| Phosphate (mmol/L) | 1.04 | 1.02 | 0.94 | 1.20 | 0.77 |
| ALP (IU/L) | 63 | 79 | 84 | 94 | 60 |
| P1NP (µg/L) | 18 | 35 | 26 | 27 | 18 |
| CTX (µg/L) | 0.13 | 0.30 | 0.15 | 0.13 | 0.11 |
| Osteocalcin (µg/L) | 10 | 23 | 14 | 21 | 14 |

[a] **Reference ranges**: adjusted calcium 2.25-2.70; phosphate 0.8-1.5; ALP 20-120; P1NP: postmenopausal female 26–110, male 20–76; CTX 0.1–0.5; osteocalcin 6.8–32.2

**Abbreviations:** BMI: Body mass index ; DXA: Dual energy X-ray absorptiometry; ALP: alkaline phosphatase; P1NP: Procollagen type 1 N-propeptide; CTX: C-terminal telopeptide type I collagen

### Oral Communications

### OC CBW1

### An adaptable 3D model of myeloma cell dormancy for pre-clinical drug screening

Alexandria Sprules[1], Caitlin Jackson[2], Alanna Green[1], Frederik Claeyssens[2], Michelle Lawson[1]

[1]The University of Sheffield, School of Medicine and Population Health, Sheffield, UK [2]The University of Sheffield, Department of Materials Science and Engineering, Sheffield, UK

### Abstract

Despite treatment advances, multiple myeloma patients encounter persistent relapse challenges. Dormant myeloma cells (DMCs), which reside in endosteal niches and interact with bone-resident cells that influence their dormancy, have been identified as contributors to relapse. Therefore, targeting DMCs is crucial so we have developed a 3D *in vitro* model replicating osteoblast-myeloma cell interactions, offering a representative microenvironment for assessing drug effects on DMCs, reducing reliance on animal models of myeloma dormancy, and increasing drug screening throughput. Our objective was to evaluate standard of care myeloma drug effects on DMCs within the 3D model and validate these results with *in vivo* DMC data. Our 3D model comprised of Polycaprolactone polymerised High Internal Phase Emulsions (PolyHIPE) scaffolds pre-seeded with osteoblast lineage cells (hFOb or MG63), then 3 days later GFP-tagged, 1,1'-dioctadecyl-3,3,3',3'-tetramethylindodicarbocyanine (DiD) stained JJN3 myeloma cells were incorporated into the model, and 24 hours later bortezomib (3 nM) was administered. Drug responses where then evaluated 72 hours post-treatment, assessing cell viability using alamarBlue™ assays and presence of DMCs using flow cytometry. For *in vivo* data*,* 6–8-week-old female NSG mice were intravenously injected with $1x10^6$ DiD-stained JJN3-GFP-luc cells, then 5 days later mice were treated with 0.5 mg/kg bortezomib or PBS (intraperitoneally) x2/week. Groups of mice were sacrificed at 7 days (n=8/group) or 19 days (n=5/group) post tumour inoculation for flow cytometric analyses of DMCs (flow panel consisted of DiD, GFP, Live/dead investigations). The 3D model revealed whole cell population (myeloma and osteoblast) sensitivity to bortezomib, with MG63 co-cultures exhibiting less cell death than hFOb co-cultures. DMC levels were similar in both co-culture systems, and more DMCs were observed on scaffolds post-drug exposure compared to vehicle controls (**Figure 1**). *In vivo*, at day 19, bortezomib-treated mice had lower tumour burden as expected, but they also had significantly higher DMCs compared to PBS vehicle treated mice. Therefore, drug efficacy was determined to be similar between the 3D *in vitro* JJN3 DMC model and the *in vivo* JJN3 model, highlighting resistance to bortezomib. In conclusion, when initially assessing drug efficacy against DMCs, our 3D *in vitro* DMC model could be used as a replacement for initial *in vivo* studies.

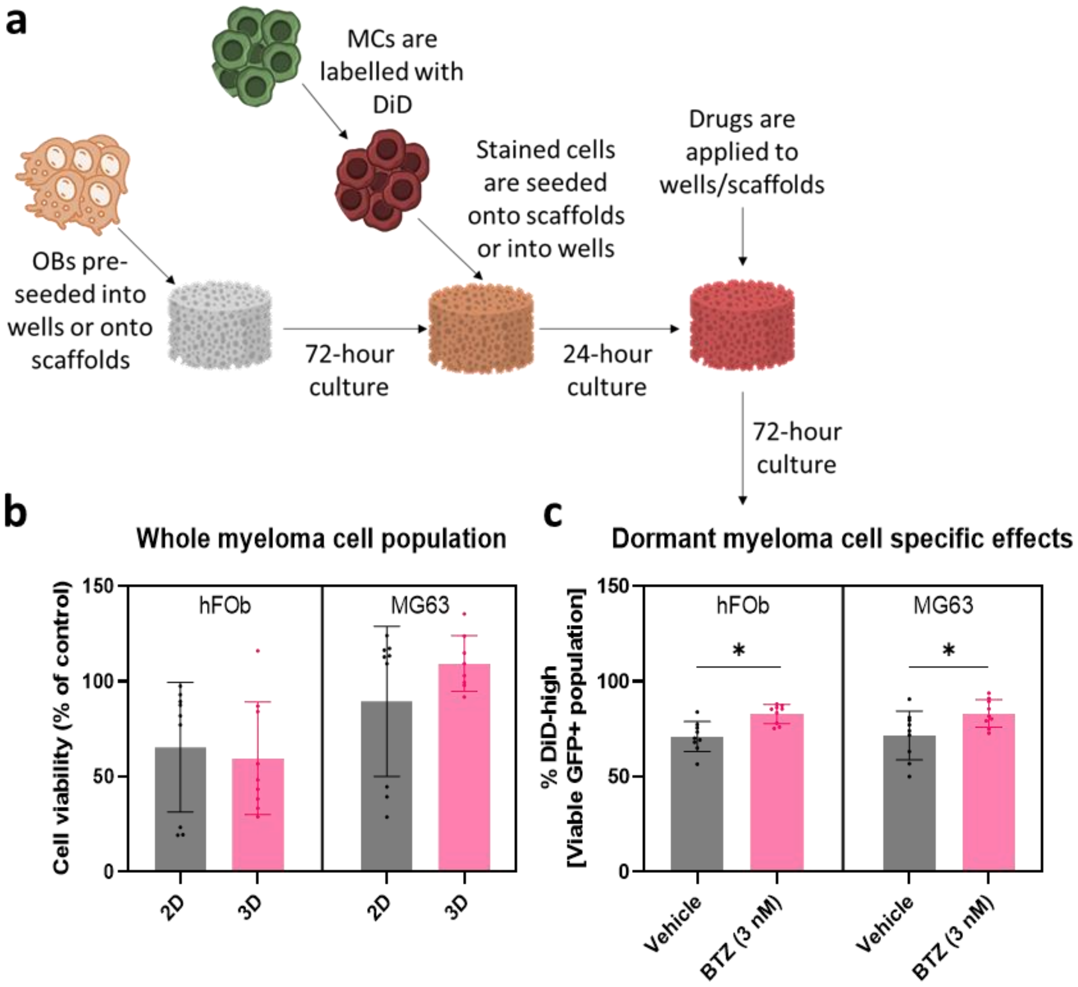

**Figure 1. Drug effects on a 3D PolyHIPE scaffold model**. a) PolyHIPE scaffolds were pre-seeded with osteoblasts (OBs) prior to the culture of pre-stained JJN3 human myeloma cells (MCs). 24 hours post MC seeding, bortezomib (BTZ) or vehicle (PBS) was incorporated into the system and cells cultured with drug for 3 days. Following this flow cytometry revealed b) whole cell population effects of bortezomib treatment and c) dormant myeloma cell specific effects.

### OC CBW2

### Multiple myeloma increases local bone mechanical anisotropy in a murine model

Saira Mary Farage-O'Reilly[1,2,3], Holly Evans[1], Rebecca Andrews[1,4], Jacob Trend[5], Goran Lovric[6], Vee San Cheong[2,7], Michelle Lawson[1], Enrico Dall'Ara[1,2,3]

[1]Division of Clinical Medicine, University of Sheffield, Sheffield, UK [2]Insigneo Institute for in silico Medicine, University of Sheffield, Sheffield, UK [3]Healthy Lifespan Institute, University of Sheffield, Sheffield, UK [4]Sheffield Teaching Hospitals, Royal Hallamshire Hospital NHS Foundation Trust, Sheffield, UK [5]School of Biological Sciences, University of Southampton, Southampton, UK [6]Swiss Light Source, Paul Scherrer Institut, Villigen PSI, Switzerland [7]Department of Mechanical Engineering, University of Sheffield, Sheffield, UK

### Abstract

Myeloma bone disease, a life-altering complication of multiple myeloma (MM), affects 80-90% of MM patients. Loss of trabecular bone and decreased bone mineral density increase the risk of fracture. Understanding how MM affects the bone mechanically could help guide the development of MM treatments. The aim of this study was to investigate if MM increases the local bone mechanical anisotropy in a murine myeloma bone disease model.

Nine 9–10-week-old NOD *scid* gamma (NOD.Cg-*Prkdc*$^{scid}$*Il2rg*$^{tm1Wjl}$*/SzJ, NSG)* mice were injected with $10^6$ U266-GFP-luc cells (disease group, n=4) or PBS (naïve group, n=5). At 10 weeks post-inoculation, all mice were sacrificed. The right femora were scanned at the growth plate using Synchrotron radiation computed tomography (SR-µCT; 0.65µm voxel size). Four cubes (side length: 80µm) were isolated from a section starting 100µm proximally from the edge of the femoral growth plate, in the anterior, posterior, medial, and lateral regions (avoiding bone lesions and blood vessels). Each image of the cube was segmented, and a micro-finite element model (micro-FE) model was generated. Each micro-FE model was loaded by a prescribed uniaxial displacement resulting in 1% apparent compressive strain along the axial, medio-lateral, or anterior-posterior direction. The normalised apparent stiffness was calculated as the ratio between the sum of the reaction forces and the applied displacement, divided by the size of the cube. The degree of anisotropy was calculated as the ratio between the maximum and minimum normalised stiffness values for each cube. Differences between the groups were evaluated using the Mann-Whitney U-test ($p<0.05$).

The normalised stiffness decreased in the anterior ($p<0.001$), posterior ($p=0.012$) and lateral ($p=0.014$) locations in the disease group when compared against the naïve group. The degree of anisotropy increased in the anterior ($p=0.032$) and lateral ($p=0.032$) locations in the disease group when compared to the naïve group.

These results suggest that MM affects the local mechanical properties of the cortical bone tissue: therefore, it is likely that it will affect the bone strength. Whether these changes can be mitigated by bone anabolic treatments remains to be investigated.

*Figure 1: Normalised stiffness (top) and degree of anisotropy (middle) boxplots for the naïve and disease groups across various locations (anterior, posterior, medial, lateral). *: Significant difference with*

*$p<0.05$. Minimum principal strain distribution (bottom) of two representative cubes (naïve - left, disease - right) taken from the anterior side, obtained using a uniaxial displacement resulting in 1% apparent compressive strain in the anterior-posterior direction.*

### OC CBW3

### Development of the chick Chorioallantoic Membrane (CAM) xenograft model to study metastasis in autophagy-deficient osteosarcoma cells

Bipusha Tha Shrestha[1], Lucy Ghali[1], Scott Roberts[2], Helen Roberts[1]

[1]Middlesex University, Hendon, UK [2]The Royal Veterinary College, London, UK

### Abstract

Osteosarcoma (OS) is the most common type of bone cancer in children. Despite advances in chemotherapy, the survival rate for metastatic OS is only 20% to 30%. We have previously shown that the activation of autophagy may be linked to metastasis and chemoresistance, leading to an unpredictable prognosis.

The Chicken Chorioallantoic Membrane (CAM) assay is an inexpensive and rapid methodology to visualise tumour development, making it a valuable tool in studying cancer metastasis. This study aimed to develop the CAM assay as a physiologically relevant model to better understand OS and how cancer markers may vary in a 3D environment when autophagy is suppressed.

In this study, the CAM assay was completed in 13-embryonic development days (EDD). After several optimisation steps for viability and tumour implantation, the experimental survival rate improved significantly to 69% ($p<0.01$), and successfully formed tumours from metastatic wild type (WT) HOS-143B cells (n=10), scrambled control (SC) (n=9), and CRISPR/Cas9 knockout (KO) ATG7 HOS-143B cells (n=9). ATG7 is an essential regulator of the autophagy process.

The role of autophagy in the *in ovo* and *in vitro* environment was found to be context-dependent. In the *in ovo*microenvironment, metastatic gene expression was downregulated in CXCR4 by 5.04-fold ($p<0.05$), CCL5 by 5.16-fold ($p<0.05$), and EZRIN by 3.75-fold in ATG7 CRISPR/Cas9 KO HOS-143B tumours compared to WT HOS-143B tumours, and CXCR4 by 1.37-fold, CCL5 by 1.59-fold, and EZRIN by 2.08-fold in ATG7 KO tumours compared to SC HOS-143B tumours. *In vitro*, ATG7 KO cells exhibited a 94.1-fold ($p<0.05$) and 2.56-fold ($p<0.05$) increase in MMP2 and MMP9 genes compared to WT and NC HOS-143B cells, respectively. However, in the *in ovo* environment, a reduction of 20.17-fold ($p<0.001$) and 14.31-fold ($p<0.05$) in MMP2 and MMP9 genes was seen compared to WT and NC HOS-143B tumours, suggesting that autophagy may trigger different signalling pathways relevant to the microenvironment. Immunohistochemistry demonstrated that WT and SC HOS-143B cells exhibited significantly greater levels of the angiogenesis marker VEGFR2, at 33.93% ($p<0.01$) and 24.40% ($p<0.05$), respectively, compared to ATG7 KO HOS-143B cells, which had reduced vascularisation.

This study contributes to our understanding of OS metastasis and also identifies autophagy as a therapeutic target to modify metastatic associated genes/proteins. Importantly, *in ovo* culturing was required to fully reveal these outcomes, thus defining the importance of considering the interaction of the microenvironment and potentially vasculature in understanding OS metastasis.

*Key words: Osteosarcoma, Chicken Chorioallantoic Membrane assay, Metastasis, angiogenesis, microenvironment.*

### OC CBW4

### Spatially resolved multi-omic profiling of metastatic osteosarcoma

Troy McEachron

National Cancer Institute, Bethesda, MD, USA

### Abstract

Osteosarcoma, the predominant malignant bone tumor in pediatric, adolescent, and young adult patients, poses a significant clinical challenge as there has been little improvement of outcome over the past four decades. Metastasis is the leading cause of death and strongest single prognosticator for patients with osteosarcoma. While various genome sequencing studies have provided impactful data on the genomic complexity of this disease, there is a paucity of data describing the microenvironment of this disease. In the current era of immunotherapy and targeted biologics, an increased comprehension of the immune and non-immune microenvironments of metastatic osteosarcoma is paramount. To address this knowledge gap we performed in-depth multi-omic profiling of osteosarcoma patient specimens, including spatial transcriptional profiling, imaging mass cytometry, single nuclei RNA sequencing, and targeted proteomic analysis. Integrated analysis of the data revealed that pulmonary metastases share a transcriptional architecture that partitions the tissues into distinct extratumoral, peritumoral, and intratumoral microenvironments based on conserved gene expression profiles across the specimens. Both spatial profiling and single nuclei RNA sequencing indicate that metastatic osteosarcoma employs various mechanisms to subvert immunotherapeutic interventions and promote disease progression including widespread immunoregulatory signaling and lymphocyte exclusion. Lastly, we employ an informatic approach to identify therapeutic modalities that target spatially distinct microdomains within the metastatic osteosarcoma microenvironment.

### OC RBD1

### Real-world experience with burosumab treatment in adults with X-linked hypophosphataemia (XLH) in the UK

Richard Keen[1], Robin Lachmann[2], Elaine Murphy[2], Gauri Krishna[2], Gavin Clunie[3], Jennifer Walsh[4], Marian Schini[4], Matthew Roy[5], Leigh Mathieson[6], Victoria Hayes[6], Ben Johnson[6], Daniel Stevens[7], Mark Nixon[8], Judith Bubbear[1]

[1]Royal National Orthopaedic Hospital, Stanmore, UK [2]University College Hospital, London, UK [3]Cambridge University Hospital Trust, Addenbrookes, Cambridge, UK [4]Sheffield Teaching Hospitals, Sheffield, UK [5]University Hospitals Bristol, Bristol, UK [6]Kyowa Kirin, Marlow, UK [7]Bionical Emas, Willington, UK [8]Chilli Consultancy, Salisbury, UK

### Abstract

Background/Introduction: XLH is a rare, genetic, progressive, phosphate-wasting disorder that causes skeletal morbidities, stiffness, pain, and impaired physical function throughout life. Burosumab is a fully human monoclonal antibody that inhibits fibroblast growth factor 23, resulting in increased renal phosphate reabsorption. An early access programme (EAP) was set up to enable access to burosumab for adults with XLH in the UK, providing an opportunity to capture real-world clinical experience with burosumab treatment.

Purpose: To describe the baseline characteristics of participants in the EAP and determine the proportion achieving a normal serum phosphate concentration (above lower limit of normal [LLN] according to local reference ranges) and mean change in serum phosphate concentration after 6 months' burosumab treatment.

Methods: This retrospective, longitudinal, real-world cohort study involved adults with XLH receiving treatment with burosumab via the EAP at five UK sites. Retrospective data were collected from medical records.

Results: A total of 142 patients were enrolled and received at least one dose of burosumab; 136 (96%) had not previously received burosumab. The patients had a mean age of 43.4 years (SD 14.7); 64% were female and 77% were white. At baseline, mean (SD) height was 155.2 (9.3) cm and weight was 73.6 (16.9) kg. Among the burosumab-naïve patients, 5% had serum phosphate concentration >LLN (95% CI 2–10%; n=127) at baseline, increasing to 63% after 6 months' burosumab treatment (95% CI 52–74%; n=82). Their mean serum phosphate concentration was 0.61 (SD 0.16) mmol/L (n=127) at baseline and had increased by 0.28 (0.24) mmol/L after 6 months' burosumab treatment (range −0.54 to 0.95 mmol/L, n=75).

Conclusions: Burosumab treatment in adults with XLH in real-world practice is associated with improvements in serum phosphate concentration to within the normal range.

### OC RBD2

### Mineral homeostasis in adults with suspected hypophosphatasia: Data from the FAME study

Tatiane Vilaca, Meena Balasubramanian, Richard Eastell

University of Sheffield, Sheffield, UK

### Abstract

Rationale and hypothesis

Hypophosphatasia (HPP) is caused by variants in the *ALPL* gene, which encodes the tissue non-specific isoform of alkaline phosphatase. The deficient enzyme activity disrupts the mineralization process and mineral metabolism. In children, hypercalcemia and hyperphosphatemia have been reported, but less is known about mineral metabolism in adults. We hypothesise that HPP affects mineral metabolism.

Objective

We aim to characterise mineral metabolism in a cohort of adults in a family mapping study (FAME) of HPP who carried a pathogenic (or likely pathogenic) variant in *ALPL*.

Methods

We recruited probands with HPP and investigated their first- or second-degree relatives. We tested for the familiar *ALPL* variant, and measured calcium (Ca), phosphate (P), parathyroid hormone (PTH), 25OH-vitamin D (25OHD), and creatinine using auto analysers, and pyridoxal 5'-phosphate (PLP) using HPLC in fasting blood samples.

Results

We recruited 70 participants: 26 probands (PROB) for *ALPL* variants, 20 positive relatives (POS) and 24 negative relatives (NEG). PROB and POS participants had lower ALP and PTH and higher P and PLP than NEG (table). There was no difference in Ca, 25OHD, creatinine or eGFR. PTH correlated negatively with P, r -0.326, p = 0.007.

Table mineral metabolism by group

| | Proband | Relative Positive | Relative Negative |
|---|---|---|---|
| Ca (nmol/L) | 2.34 (0.08) | 2.34 (0.08) | 2.34 (0.07) |
| P (nmol/L) | 1.22 (0.18)** | 1.36 (0.18)*** | 1.04 (0.20) |
| ALP (IU/L) | 26.0 (21-35)*** | 28.5 (24.5-40.5)*** | 81 (60.5–108.5) |
| 25OHD (nmol/L) | 60.9 (20.6) | 50.8 (19.1) | 50.9 (20.8) |
| Creatinine(umol/L) | 72.2 (11.1) | 70.3 (10.5) | 79.8 (19.5) |
| eGFR(ml/min.1.73m2) | 90 (81-90) | 90 (81-90) | 88 (67-90) |

| | | | |
|---|---|---|---|
| PTH(pmol/L) | 4.1 (3.5-5.2) | 3.7 (2.7-4.9)* | 4.9 (4.0-6.1) |
| PLP (nmol/L) | 137 (81-345)*** | 204 (108-315)*** | 37.5 (18.5-60.0) |

Variables are shown as mean (standard deviation) or median (interquartile range)

*p<0.05 **p=<0.01 ***p=<0.001

Conclusion

Pathogenic variants in *ALP* are associated with higher phosphate and lower PTH levels. Lower PTH might contribute the increase in P levels.

### OC1.1

### Porcupine inhibition as a novel therapeutic paradigm for sclerosteosis

Timothy J Dreyer[1], Jacob A C Keen[1], Mark Hopkinson[1], Gill Holdsworth[2], Andrew A Pitsillides[1], Scott J. Roberts[1]

[1]Royal Veterinary College, London, UK [2]UCB Pharma, Slough, UK

### Abstract

Sclerosteosis (OMIM accession number 269500) is an ultra-rare high bone mass (HBM) disorder, for which there is no available pharmacological treatment - acute clinical symptoms are currently managed through high-risk surgery. Sclerosteosis is caused by autosomal recessive *SOST* mutations that result in loss of functional sclerostin, which plays a pivotal role in bone formation by antagonising the Wnt/β-catenin signalling pathway. LGK974, a small molecule porcupine (PORCN) inhibitor, suppresses Wnt signalling by blocking palmitoylation and transport of Wnt ligands. Herein we assessed LGK974's potential as a novel sclerosteosis therapeutic.

*In vitro* cell assays explored the effect of LGK974 on osteoblast and primary osteoclast biology. To assess HBM reduction potential, 6-week-old male and female Sost deficient (*Sost*$^{-/-}$; model of sclerosteosis) mice were treated intermittently with 6 mg/kg LGK974 or DMSO control for 4 weeks (n = 20). Right tibiae were loaded (20N peak load) to simulate early childhood loading events. Collected tibiae, vertebrae and skulls were analysed by microcomputed tomography.

Analogous to sclerostin, LGK974 (100 nM) effectively reduced *in vitro* osteoblast alkaline phosphatase (ALP) activity and mineralisation (reduced by 65.9% and 95.4%, respectively; $p < 0.0001$) and decreased Axin2 (Wnt marker), Runx2 and OCN (osteoblast markers) expression (decreased by 17.8%, 42.4% and 98.6%, respectively; $p < 0.0001$), whilst primary osteoclast number and resorption remained unaffected. In *Sost*$^{-/-}$ mice, LGK974 treatment significantly reduced skull bone volume (BV; 11.6%; $p < 0.05$) and parietal bone area (BA; <10%; $p < 0.05$) and thickness (<20%; $p < 0.05$) in males. Otosclerosis was decreased in male ossicles (BV and BA reduced by 10.6% ($p < 0.01$) and 14.3% ($p < 0.0001$), respectively) and otic capsules (BA; 7.8% ($p < 0.05$)). A significant reduction in male and female trabecular number (reduced by 19.9% and 18.7%, respectively; $p < 0.0001$) was recorded in lumbar vertebrae of treated mice. Treatment reduced cortical BV in male and female loaded (reduced by 19.2% and 16.6%, respectively; $p < 0.0001$) and non-loaded (reduced by 18.2% and 18.1%, respectively; $p < 0.0001$) tibiae. Interestingly, sexual dimorphism was observed when comparing male and female *Sost*$^{-/-}$ mouse groups, aligning with data from human sclerosteosis patients and heterozygous carriers.

This study discloses that PORCN inhibition reduces bone mass in multiple sites associated with the most severe pathology in sclerosteosis (skull and ear), as well as the spine and limbs. These data provide the first evidence of a disease modifying treatment strategy to alleviate symptoms of sclerosteosis and potentially other *Wnt* related HBM conditions.

### OC1.2

### Engineering textured polymeric microparticles as cell-instructive bone matrix mimetics via activation of the hedgehog signalling pathway

Fatmah Ghuloum[1,2], Marco Domingos[1], Susan Kimber[1], Mahetab Amer[1]

[1]The University of Manchester, Manchester, UK [2]Kuwait University, Kuwait City, Kuwait

### Abstract

Engineering three-dimensional (3D) biomaterials which emulate biophysical properties of bone matrix, such as stiffness and topographical features, have demonstrated their capacity to stimulate osteogenesis in bone marrow-derived primary human mesenchymal stem cells (hMSCs) without requiring external osteo-inductive supplements. However, the molecular mechanisms underpinning this osteo-inductive property are unclear. The activation of Hedgehog (HH) signalling, a key pathway required in early stages of bone development, can be mechanically regulated in response to external stimulation during osteogenic differentiation. The aim of the study is to explore the correlation between the osteo-inductive effect of 3D topographically-textured polymeric microparticles and the activation of the HH signalling pathway *in vitro*. Poly(D,L-lactic acid) (PDLLA) microparticles with varying micro-topographical features, and human trabecular bone-mimetic elastic moduli, were fabricated by a solvent evaporation oil-in-water emulsion technique within the size range of 40-70µm. Primary hMSCs (3 females and 2 males, aged 19-34 years) were cultured on microparticles of varying designs and on tissue culture plastic (as 2D controls). Viability, proliferation and various markers of osteogenesis and HH signalling (N≥5) were assessed. hBM-MSCs showed excellent viability and proliferation up to day14 when seeded on the fabricated microparticles. Culture of hBM-MSCs on dimpled microparticles significantly increased the expression levels of several early and late markers of osteogenesis, including *RUNX2* (2.52-fold, $p \leq 0.01$) at day3, and *BGLAP* (2.25-fold, $p \leq 0.01$), *SPP-1* (8.43-fold, $p \leq 0.01$) and *IBSP* (2.34-fold, $p \leq 0.05$) at day10 relative to smooth microparticles. This osteo-inductive effect observed with dimpled microparticles coincided with a significant increase in the expression levels of HH signalling pathway components at day 3 relative to smooth microparticles, including *GLI1* (3.14-fold, $p \leq 0.05$), *SMO* (1.92-fold, $p \leq 0.05$), *PTCH1* (2.05-fold, $p \leq 0.01$) and *SHH* (3.99-fold, $p \leq 0.001$). Remarkably, the increase in *GLI1* expression induced by dimpled microparticles was comparable to the effect induced by purmorphamine, a widely used SMO receptor agonist. The involvement of the canonical HH activation was confirmed by the significant reduction in the expression of *GLI1* ($p \leq 0.05$) and *BGLAP* ($p \leq 0.01$) following treatment with KAAD-cyclopamine (a SMO receptor inhibitor) on cells cultured on dimpled microparticles relative to vehicle-treated counterparts. Our data demonstrates that topographically textured microparticles induce osteogenesis in hMSCs via the activation of the canonical Smo-dependent HH pathway. Better understanding of the regulation of HH signalling in mechanosensitive hMSCs will offer novel opportunities for exploring innovative approaches to model HH-related bone diseases and uncover potential ECM-related therapeutic targets.

### OC2.1

### The prevalence of vertebral fractures and associated factors in The Gambia, South Africa and Zimbabwe: The Fractures-E3 study

Lucy Gates[1], Anya Burton[2], Tadios Manyanga[3], Momodou Jallow[4], Bilkish Cassim[5], Christopher Grundy[6], Hannah Wilson[2], Rashida Ferrand[3,6], Farhanah Paruk[5], Nicola Crabtree[7], Emma Clark[2], Celia Gregson[2,3], Kate Ward[1,4]

[1]University of Southampton, Southampton, UK [2]University of Bristol, Bristol, UK [3]Biomedical Research and Training Institute, Harare, Zimbabwe [4]London School of Hygiene and Tropical Medicine, Banjul, Gambia [5]University of KwaZulu-Natal, Durban, South Africa [6]London School of Hygiene and Tropical Medicine, London, UK [7]Birmingham Women's and Children's NHS Trust, Birmingham, UK

### Abstract

Vertebral fractures (VFs) are the most common osteoporotic fracture. Few data describe the prevalence and associated factors for VFs from resource-limited settings, where the populations are rapidly ageing. We aimed to determine the prevalence and associated factors for VF in women and men in the Fractures-E3 study[1].

A population-based, cross-sectional study of community dwelling adults was conducted across three urban sites; Harare, Zimbabwe; Brufut/Sukuta, The Gambia; Kwamashu, South Africa. Recruitment was stratified in sex and age groups (40-54, 55-69, ≥70 years). Prevalence of VFs was determined using Genant semi-quantitative assessment on iDXA images in The Gambia and Zimbabwe, and lateral thoracic/lumbar spine radiographs in South Africa. Demographic and clinical factors were compared in those with and without prevalent VFs, by country and sex. Logistic regression determined associations between Vrac clinical decision tool[2] factors: age, weight, wall-to-tragus, back pain, prior fragility fracture, and glucocorticoid use.

Mean BMI (kg/m$^2$) was higher in women for all countries (Gambia: 28.0 [6.1] vs 23.6 [4.1], South Africa (34.9 [9.0] vs 25.4 [5.8] and Zimbabwe (28.9 [6.7] vs 23.0 [4.2]). Prevalence of VF was 10.6% (n=116) in The Gambia, 4.1% (n=39) in South Africa and 7.7% (n=84) Zimbabwe. Those with VF were older; The Gambia (69.6 (10.1) vs 59.3 (12.4) years, $p<0.001$), South Africa (66.4 (11.4) vs 59.8 (11.9) years, $p<0.001$) and Zimbabwe (71.6 (13.4) vs 61.6 (13.9) years, $p<0.001$). Sex was not associated overall, however VF prevalence was higher in younger men (in Gambia and South Africa) and higher in older women (Table 1). The odds of having a prevalent VF were higher in those who reported a prior adult fracture (Odds ratios [95% confidence intervals] Gambia 2.29 [0.96,5.47]; South Africa 2.28 [1.18,4.41]; Zimbabwe 2.18 [1.15,4.13]). Increasing weight in South Africa (0.98 [0.96,0.99]) and Zimbabwe (0.98 [0.97,1.00]) was associated with lower odds of having a prevalent VF and greater odds of VF with current steroid use in The Gambia (2.95 [1.47,5.95]). The remaining Vfrac variables were not associated with having a prevalent fracture.

VFs are common in African populations. Given the rising ageing population in countries with limited and stretched healthcare resources, it is important to understand further context specific factors associated with fragility fractures such as bone trauma in young, alcohol consumption, the role of fat mass, bone mineral density and HIV.

Table 1. Age group differences in VF prevalence by sex

| | | Gambia (n=1100) | | | South Africa (n=960) | | | Zimbabwe (n=1087) | | |
|---|---|---|---|---|---|---|---|---|---|---|
| | | Age group (years) | | | | | | | | |
| | | 40-54 (n=361) | 55-69 (n=348) | ≥70 (n=388) | 40-54 (n=344) | 55-69 (n=337) | ≥70 (n=273) | 40-54 (n=356) | 55-69 (n=355) | ≥70 (n=375) |
| Prevalent VF, n (%) | | 7 (1.9) | 30 (8.6) | 79 (20.4) | 7 (2.0) | 14 (4.2) | 118 (6.6) | 13 (3.7) | 18 (5.1) | 53 (14.1) |
| Sex, n (%) | Male | 4 (57.1) | 15 (50.0) | 32 (40.5) | 5 (71.4) | 7 (50.0) | 6 (33.3) | 8 (61.5) | 8 (44.4) | 22 (41.5) |
| | Female | 3 (42.9) | 15 (50.0) | 47 (59.5) | 2 (28.6) | 7 (50.0) | 12 (66.7) | 5 (38.5) | 10 (55.6) | 31 (58.5) |

*1. Burton et al. Wellcome Open Research, 2023 10.12688/wellcomeopenres.19391.1*

*2. Khalid TY et al. Arch Osteoporos. 2024 Feb 7;19(1):12.*

### OC2.2

### Associations between body mass index, bone mineral density and risk of hip fracture differ by sex: An international meta-analysis

Nicholas Harvey[1], Helena Johannson[2], Eugene McCloskey[3], Enwu Liu[4], Marian Schini[5], Liesbeth Vandenput[6], Mattias Lorentzon[7], William Leslie[8], John Kanis[2], FRAX Meta-analysis Cohort Group[1]

[1]MRC Lifecourse Epidemiology Centre, University of Southampton, Southampton, UK [2]Centre for Metabolic Bone Diseases, University of Sheffield, Sheffield, UK [3]MRC Versus Arthritis Centre for Integrated research in Musculoskeletal Ageing, Mellanby Centre for Musculoskeletal Research, University of Sheffield, Sheffield, UK [4]Flinders University, Adelaide, UK. [5]Mellanby Centre for Musculoskeletal Research, Department of Oncology and Metabolism, University of Sheffield, Sheffield, UK [6]Sahlgrenska Osteoporosis Centre, Department of Internal Medicine and Clinical Nutrition, Institute of Medicine, Sahlgrenska Academy, University of Gothenburg, Gothenburg, Sweden [7]Sahlgrenska Osteoporosis Centre, Institute of Medicine, University of Gothenburg, Gothenburg, Sweden [8]Department of Medicine, University of Manitoba, Winnipeg, Canada

### Abstract

### Objectives

We aimed to quantify, in the context of this international meta-analysis, the predictive value of body mass index (BMI) for incident hip fracture, and to investigate the relationship of this risk with sex and femoral neck bone mineral density (BMD).

### Materials and methods

The interim analysis dataset comprised 172,059 men and women with BMD measured, from 51 cohorts in 20 countries, with a total follow-up time of 1.4 million person-years. We used an extended Poisson model in each cohort to investigate relationships between WHO-defined BMI categories (Underweight: $<18.5 kg/m^2$; Normal: $18.5$-$24.9 kg/m^2$; Overweight: $25.0$-$29.9 kg/m^2$; Obese I: $30.0$-$34.9 kg/m^2$; Obese II: $\geq 35.0 kg/m^2$) and risk of hip fracture (HF). Age, sex, BMD and duration of follow-up were considered as covariates. The inverse-variance weighted β-coefficients were used to merge the results from the individual cohorts.

### Results

In models adjusted for age and time since baseline, lower BMI was associated with a greater risk of incident hip fracture in both sexes. Comparing underweight individuals with normal weight, the hazard ratio [95% CI] for HF was 2.30 [2.03-2.60] in women and for men was 3.51 [2.05-5.99]. In both sexes, HF risk was lower in overweight and obese categories compared to normal weight [obese II vs normal: women 0.71 [0.58-0.88]; men 0.95 [0.94-0.97]].

Further adjustment for femoral neck BMD T-score attenuated the increased risk associated with underweight [underweight vs normal: women 1.72 [1.44-2.06]; men 1.90 [1.05-3.45]].In these models, the protective effects of overweight and obesity were attenuated, and in both sexes inverted in the Obese II category. [obese II vs normal: women 1.32 [1.02-1.71]; men 2.07 [1.36-3.14]].

**Conclusions**

These findings, which will inform the next version of FRAX®, demonstrate that underweight is a risk factor for hip fracture in both men and women regardless of adjustment for BMD. However, whilst overweight and obesity appeared protective in age- and follow-up time adjusted models, they became risk factors after additional adjustment for femoral neck BMD, particularly in the Obese II category. This effect in the highest BMI categories appeared of greater magnitude in men than women. UK Biobank data are included under approved access agreement 3593.

### OC3.1

### Delineating the role of hypoxic extracellular vesicles in pulmonary metastasis of osteosarcoma

Rianna Koshy, Shresth Modi, Amy Wyatt, Luke Tattersall, Mark Collins, Alison Gartland, Karan Shah

The University of Sheffield, Sheffield, UK

**Abstract**

Osteosarcoma (OS) is the most common primary malignant neoplasm of the bone in children and adolescents, with a high propensity for metastasis to lungs. It has now become evident that primary tumours including OS secrete factors sequestered in extracellular vesicles (EVs) that make distant target organs more conducive for colonisation and metastases, creating the premetastatic niche (PMN). Here, we present exciting new data which shows a predominant role of the hypoxic subpopulation of OS EVs in the formation of PMN in the lungs.

EVs were isolated from human 143B-luciferase positive osteosarcoma cells cultured in normoxic (21%$O_2$) or hypoxic (1%$O_2$) conditions and characterised using western blotting and mass-spectrometry. Human alveolar basal epithelial A549 cells were exposed to the hypoxic and normoxic OS EVs and changes in their cytokine-chemokine secretion were assessed using the Proteome-Profiler Cytokine Array. To assess the effects of the EV sub-populations *in-vivo*, 8-week old female Balb/C nude mice (5/group) were injected with 0.5µg hypoxic, normoxic EVs or vehicle administered via alternate subcutaneous and intravenous injections every other day for 2 weeks. Lung tissues were collected and examined by immunohistochemical staining for ECM remodelling (fibronectin and periostin) and immune cells infiltration (neutrophils and macrophages).

We observed that acute exposure to hypoxic EVs (500 EVs/cell) altered the cytokine secretion profile of lung cells *in vitro*, with increased THBS1 (promotes tumour dormancy), VEGFA (increases microvessel permeability) and MMP9 (ECM remodelling). In the *in vivo* model, administration of hypoxic EVs altered the ECM composition with increased expression of periostin in hypoxic EV treated group compared to controls ($P<0.05$), and increased infiltration of macrophages in the lungs compared to normoxic EVs and vehicle controls ($P<0.01$).

This study shows that changes characteristic of lung PMN are mediated predominantly by hypoxic OS EVs and further research is required to evaluate the therapeutic potential of targeting these EVs for delaying or preventing metastasis or as a biomarker for early detection of metastases.

### OC3.2

### TNAP and PHOSPHO1 function synergistically to afford critical control over the initiation of bone mineralisation: Skeletal phenotyping of the double knockout mouse

Lucie Bourne[1], Aikta Sharma[2], Scott Dillon[3], Soher Jayash[4], Sonoko Narisawa[5], Kyoco Tan[1], Munira Osman[1], Humayra Iqbal[1], Louise Stephen[4], Brian Foster[6], José Luis Millán[5], Colin Farquharson[4], Katherine Staines[1]

[1]Centre for Lifelong Health, University of Brighton, Brighton, UK [2]UCL Mechanical Engineering, University College London, London, UK [3]Wellcome-Medical Research Council (MRC) Cambridge Stem Cell Institute, University of Cambridge, Cambridge, UK [4]The Roslin Institute and Royal (Dick) School of Veterinary Studies, University of Edinburgh, Edinburgh, UK [5]Sanford Burnham Prebys Medical Discovery Institute, San Diego, USA [6]Division of Biosciences, College of Dentistry, The Ohio State University, Columbus, USA

### Abstract

Phosphatases that liberate inorganic phosphate are essential for biomineralisation. TNAP and PHOSPHO1 play major roles, nevertheless, it is unknown whether both phosphatases co-ordinate their functions to control the initiation of this process in the skeleton. We generated mice with a conditional *Prx1*-specific deletion of TNAP on a global *Phospho1*$^{-/-}$ genetic background (*Alpl*$^{fl/fl}$;*Prx1-Cre*$^{+/-}$;*Phospho1*$^{-/-}$).

*Alpl*$^{fl/fl}$;*Prx1-Cre*$^{+/-}$;*Phospho1*$^{-/-}$ animals did not survive post-weaning. MicroCT analysis revealed that 1-day-old *Alpl*$^{fl/fl}$;*Prx1-Cre*$^{+/-}$;*Phospho1*$^{-/-}$ mice have severely hypomineralised skulls and almost non-existent mineralisation of long bones. Thus, to align with humane practices in animal research, *Alpl*$^{+/fl}$;*Prx1-Cre*$^{+/-}$;*Phospho1*$^{-/-}$ mice are being generated, which survive to skeletal maturity. Our analyses are ongoing to conduct full skeletal phenotyping in 6-week-olds, and to examine how these differ to *Alpl*$^{fl/fl}$;*Prx1-Cre*$^{+/-}$ and wild-type mice, which have not yet been examined at 6-weeks, or in both sexes.

Conditional *Alpl* deletion was confirmed by RT-qPCR showing a ≤175-fold reduction in *Alpl* gene expression in femurs, compared to wild-type ($p<0.05$). Whilst there was no change in body weight or nose-to-tail length, *Alpl*$^{fl/fl}$;*Prx1-Cre*$^{+/-}$ mice displayed a reduction in tibial bone length ($p<0.01$). *Alpl*$^{fl/fl}$;*Prx1-Cre*$^{+/-}$ mice exhibited an increase in growth plate width ($p<0.05$) and bridge formation ($p<0.01$). MicroCT analysis revealed trabecular parameters (BV/TV, intersection surface, trabecular number, connectivity density) were reduced in *Alpl*$^{fl/fl}$;*Prx1-Cre*$^{+/-}$ mice ($p<0.05$), whereas trabecular spacing and pattern factor were increased ($p<0.001$). This detrimental effect was also observed in the epiphysis ($p<0.05$). Further, whole-bone cortical analyses revealed that whilst *Alpl*$^{fl/fl}$;*Prx1-Cre*$^{+/-}$ mice from both sexes had reduced bone area and cortical thickness ($p<0.05$), parameters associated with geometry ($J$, $I_{max}$, $I_{min}$) were only altered in male mice ($p<0.05$). Mechanical testing also revealed this sex difference. Whilst stiffness was reduced in both sexes ($p<0.01$), *Alpl*$^{fl/fl}$;*Prx1-Cre*$^{+/-}$ male mice displayed reduced maximum load ($p$=0.0078) and yield ($p$=0.001), whereas females were unaffected. Backscattered scanning electron microscopy revealed focal hypomineralisation in *Alpl*$^{fl/fl}$;*Prx1-Cre*$^{+/-}$ bones, accompanied by an altered distribution of osteocytes and evidence of mineralisation defects surrounding osteocyte lacunae, compared to wild-type. The skull phenotype was largely unaltered in

*Alpl*$^{fl/fl}$;*Prx1-Cre*$^{+/-}$ of either sex. Similarly, no dental phenotype was evident. Gene expression of *Enpp1*, *Phospho1*, *Bglap* and *Spp1* were reduced ≤5-fold ($p<0.05$) in *Alpl*$^{fl/fl}$;*Prx1-Cre*$^{+/-}$ femurs.

Data here suggests that whilst in young adult animals conditional *Alpl* deletion phenocopies hypophosphatasia, sexual dimorphisms may exist. Further, the striking phenotype upon additional deletion of PHOSPHO1 suggests the synergistic actions of these two phosphatases are critical for effective biomineralisation. Ongoing characterisation of *Alpl*$^{+/fl}$;*Prx1-Cre*$^{+/-}$;*Phospho1*$^{-/-}$ mice will enable further delineation of this interaction.

### OC4.1

### Osteoclast-induced modulation of osteosarcoma migration: Exploring the role of CSF3/STAT3/MMP9

Daniela Paternina Martinez[1], Scott J Roberts[2], Helen C Roberts[1]

[1]Middlesex University, London, UK [2]Royal Veterinary College, London, UK

### Abstract

Metastatic disease significantly impacts the prognosis of osteosarcoma (OS) patients, with survival rates ranging from 20% to 30% for metastatic disease compared to up to 80% for nonmetastatic. Unfortunately, this figure has plateaued over the past few decades, partly due to a paucity of research into mediators of the metastatic process. Recent evidence shows that targeting osteoclasts (OCs) with zoledronic acid (ZA) increases the number of metastatic lung lesions clinically, whereas treatment with fulvestran increases OC numbers and decreases metastatic lesions. This links OC function to OS metastasis.

Through an in vitro model of OS metastasis, we have found that the highly metastatic OS cell line 143B contains a subset of highly migratory spheroids with enhanced expression of stemness and metastasis-associated genes Oct3/4 (10-fold ($p \leq 0.001$)), Nanog (4-fold; $p \leq 0.001$), Sox2 (6-fold; $p \leq 0.001$), and CXCR4 (2-fold; $p \leq 0.001$) compared to monolayer cells. We have termed these spheroids with enhanced migratory capabilities 'Migratory Bodies' (MBs).

We subsequently assessed the effect of OC-released factors on MB formation, where a significant reduction in MB formation in 143B cells cultured with OC-conditioned media compared to control (43%; $p \leq 0.001$) or conditioned media from OCs treated with ZA (OC-ZA, 33%, $p \leq 0.001$) was observed. Furthermore, 143B cells cultured with OC-conditioned media or OC-ZA-conditioned media exhibited divergent gene expression as assessed by RNA-sequencing. Differentially expressed genes were validated by RT-qPCR; MMP9 (2.4-fold decrease; $p \leq 0.001$), MMP14 (2-fold decrease; $p \leq 0.01$), CSF3 (2.5-fold decrease; $p \leq 0.01$) and SPOCK (1.6-fold decrease; $p \leq 0.01$). In an attempt to mimic OC-conditioned media, chemical inhibitors of MMP9 and STAT3 (downstream of CSF3) were tested in the MB metastasis assay. Both STAT3 and MMP9 expression is linked to lower survival in OS patients using the R2: Genomics Analysis and Visualization Platform. Inhibition of STAT3 and MMP9 significantly reduced MB formation from 143B cells by 76% and 31% ($p \leq 0.001$) respectively, compared to control.

Collectively, these data indicate that OCs release factors that repress MB formation in highly metastatic 143B cells. This supports our hypothesis that OS may reside in the bone environment due to interaction with bone cells. Contrary to Paget's "seed and soil" hypothesis, it appears that disruption of this interaction leads to increased incidence of pulmonary metastasis . Lastly, our data suggests a novel approach to combat highly metastatic and migratory osteosarcoma cells by targeting STAT3 and/or MMP9.

### OC4.2

### Patient specific mechanobiological model of bone adaption in vertebrae with multiple myeloma

Fiona Gibson[1,2], Julia Shelton[3], Sean Molloy[4], Margaret Paggiosi[1,2], Catherine Handforth[1], Janet Brown[1], Xinshan Li[1,2], Enrico Dall'Ara[1,2], Stefaan Verbruggen[3]

[1]The University of Sheffield, Sheffield, UK [2]Insigeno Institute for in silico medicine, Sheffield, UK [3]Queen Mary University of London, London, UK [4]Royal National Orthopaedic Hospital, London, UK

### Abstract

### Introduction

Vertebral lytic lesions are a common complication of multiple myeloma (MM), a cancer of the plasma cells. Patients with MM are most affected by spinal involvement (80-90%) [1]. The standard of care is invasive surgical intervention when patients present with spinal instability. However, the surgery is associated with increased morbidity and high infection risk [2]. A non-surgical strategy has been adopted through externally bracing the spine whereby bone apposition has been observed [3,4]. However, it is unclear whether this is mainly mechanobiologically driven or mainly due to other biological mechanisms.

### Method

A mechanobiological model was developed using control data (8 non-cancer volunteers) and tested on MM patients (7 all braced). 3D finite element (FE) models of the vertebra were generated from the quantitative computed tomography (CT) scans performed at baseline and follow up. CT images were densitometrically calibrated with a phantomless approach. Bone was modelled as heterogenous (based on bone mineral density, BMD), isotropic, and elastic-plastic. Uniaxial compression (0.15% strain, ANSYS) was simulated, and failure load (FL) was defined as the load at 1.9% strain. The mechanobiological model was constructed by looping the organ-level FE model to a cell-level model that updated the material properties depending on biological pathways described by differential equations (Figure 1) [5,6]. Remodelling parameters $\alpha_1$, $\alpha_{2,\beta}1$, $\beta_2$, $k_1$ and $k_2$ were optimised for each patient (Figure 1).

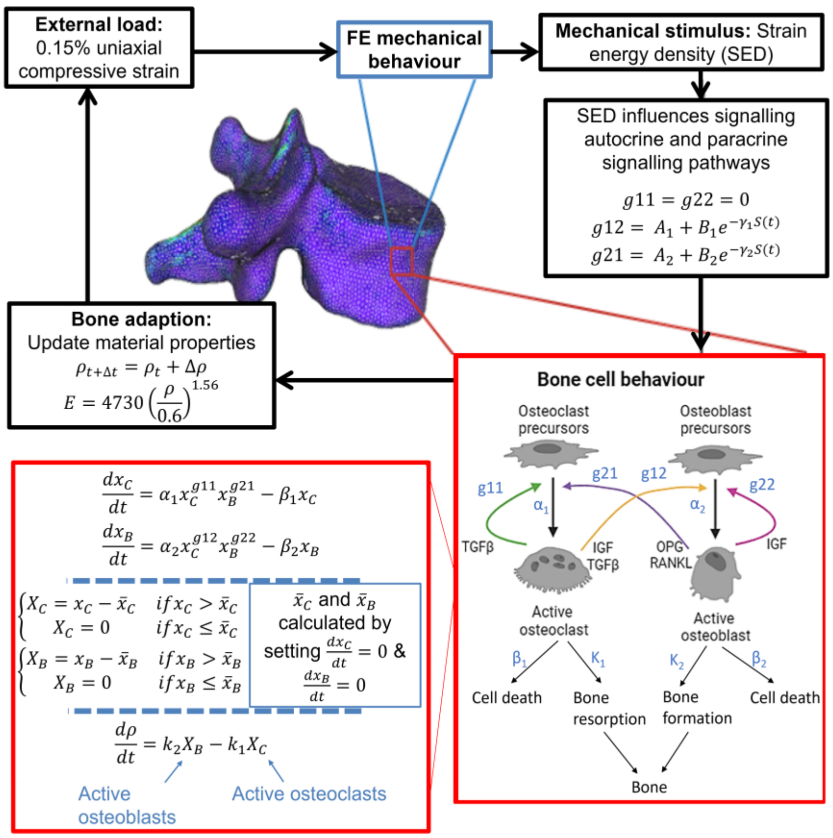

***Figure 1. Flow chart of mechanobiological model***

### Results

Figure 2 shows the model's predictive ability of average BMD and FL was higher in the control group than the MM group (control $R^2$=0.9753 for BMD and $R^2$=0.9885 for FL, MM $R^2$ = 0.8708 for BMD and $R^2$ = 0.7691 for FL).

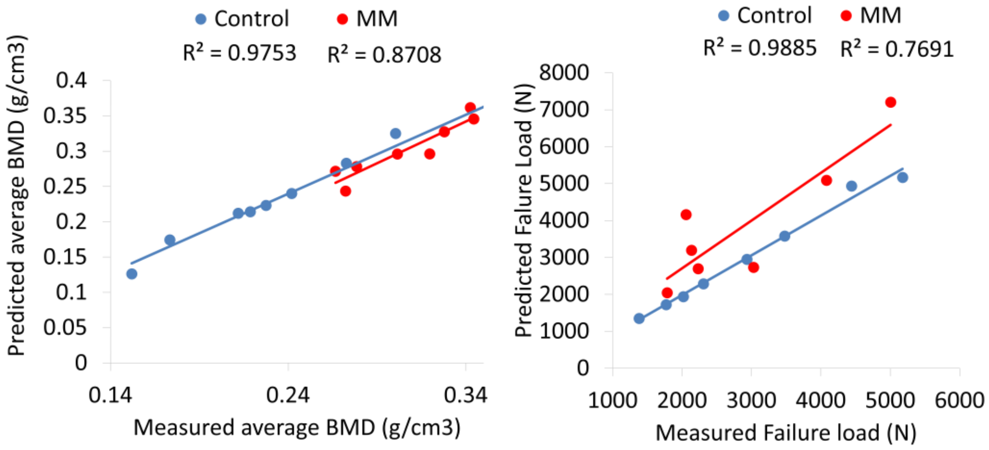

***Figure 2. Mechanobiological model predicted the average BMD and failure load better in the control group than the MM group.*** *Regression plots of measured vs predicted average BMD (left) and FL (right).*

### Discussion

Optimising the six parameters in the mechanobiological model was enough to predict well the average BMD and FL in the control group but not the MM group. This could be explained by the inclusion of lytic lesions that would alter strain distribution, lesions located at different levels in the spine that would alter transverse load distribution, and collapsed vertebra that would impact the local concentration of the loading. Further work to include a biological mechanism associated with MM will be carried out to improve the predictions of the model.

<u>References</u>

1. Bird et al., Br. J. Haematol., 2011
2. Nucci and Anaissie, Clin. Infect. Dis., 2009
3. Malhotra, et al., Spine J., 2016
4. Malhotra et al., BMC Cancer, 2016
5. Hambli, Frontiers in Bioeng and Biotech, 2014
6. Komarova, et al., Bone, 2003

### OC5.1

### CYCLIN-past-OA: CDK8/19 inhibition as an osteoarthritis therapeutic strategy

Leah M Wells, Jacob A C Keen, Andrew A Pitsillides, Scott J Roberts

The Royal Veterinary College, London, UK

### Abstract

Osteoarthritis (OA) is a whole joint disease characterised by cartilage mineralisation/degeneration, subchondral bone remodelling, osteophytogenesis and synovial inflammation. Chondrocyte hypertrophy is a key hallmark of OA that drives cartilage catabolism and promotes other aspects of joint pathology. There are, however, no approved disease-modifying therapeutics and pain management is suboptimal. This creates an urgent need for novel OA therapeutics.

To identify modulators of chondrocyte mineralisation/hypertrophy, we developed a novel *in vitro* screening platform using alcian blue and alizarin red staining applied to ATDC5 micromass cultures. Screening a library of epigenetic modulators using this platform pinpointed CDK8/19, and its inhibitor (CDK8/19i), neither of which had previously been linked with OA. Transcriptomic analysis via RNAseq of chondroprogenitor cells (N=3) revealed that CDK8/19i treatment increased levels of pro-anabolic/anti-catabolic markers (p=<0.0001), including ACAN and COL2A1 (>5-fold), CNMD and TIMP4 (>100-fold) and downregulated multiple hypertrophy markers (p=<0.0001) such as MEF2C (3-fold), IHH (5-fold) and IBSP (68-fold).

Notably, primary cells differentiated for 12 days in the presence of CDK8/19i displayed pro-anabolic behaviours and protection against IL-1β-induced ECM degradation in human (e.g. MATN3: -IL1β 100-fold, p=<0.05; +IL1β 12-fold, p=<0.05; N=1) and equine chondrocytes (e.g. MATN3: -ILβ 100-fold, p=0.28; +IL1β 16-fold, p=<0.05; N=6). CDK8/19i also modified cell metabolism in hypertrophic cells with a 1.4-fold reduction (p=<0.0001) in glycolytic energy production (MitoStress Test, Agilent), whilst simultaneously reducing inflammatory processes in THP-1-derived macrophages (expression; TNFα 2-fold, IL1β and IL6 10-fold; p=<0.01; IL1β production 1.5-fold, p=<0.05) and primary equine synoviocytes (IL6 expression 5-fold, p=<0.001) stimulated with pro-inflammatory mediators such as LPS (macrophages) and IL1β (synoviocytes).

Treatment of spontaneous OA STR/ort mice with CDK8/19i resulted in 2.2-fold decrease in weight gain (p=<0.01) following 12-week oral dosing (DMSO N=5, CDK8/19i N=6) and serum analysis revealed a trend to decreased malondialdehyde (MDA; 2.2-fold p=0.056), an OA-associated oxidative stress marker. Additionally, a trend for reduced IL-1β levels (~2-fold) was observed. Vitally, CDK8/19i displayed no anabolic effect following subchondral bone analysis, with a correction in gait symmetry (1:1 left to right ratio p=<0.05) and treadmill task completion (100% with CDK8/19i Vs 60% with DMSO) rates observed.

Collectively, these data disclose CDK8/19 inhibition as a promising strategy for OA therapy, and pinpoint inhibition of CDK8/19 as a disease-modifying strategy via its pivotal roles in controlling multiple cellular events crucial to joint pathology. Control of multiple cellular behaviours is likely essential in OA as targeting single disease-linked factors (e.g., IL1β and ADAMTS5) has failed.

### OC5.2

### Influence of resistance training on tibial cortical and trabecular bone in postmenopausal women: The REPROOF Study

Ogulcan Caliskan[1], Elisa Marques[1,2], Jonathan Folland[1,3,4], Katherine Brooke-Wavell[1]

[1]Loughborough University, Leicestershire, UK [2]University of Kalba, Kalba, UAE [3]Versus Arthritis Centre for Sport, Exercise and Osteoarthritis, Loughborough Univerisity, Leicestershire, UK [4]National Institute for Health and Care Research (NIHR) Leicester Biomedical Research Centre, Leicestershire, UK

### Abstract

**Background:** Conventional progressive high-load resistance training (CRT) is recommended for optimising bone strength. Ballistic resistance training (BRT) aims to maximise power production by attempting to launch a light/moderate load with an explosive intent throughout the concentric phase (i.e. jumping) and generating impact forces. Preliminary findings showed that BRT, but not CRT, increased proximal femur BMD. Effects of BRT on bone structural parameters, that also contribute to bone strength, have not been explored.

**Aim** This study aimed to investigate the effects of ballistic and conventional RT, relative to control, on tibial structural parameters in postmenopausal women.

**Methods** The Resistance Exercise Programme on Risk of Osteoporosis and Osteoarthritis in Females (REPROOF) study recruited healthy, postmenopausal (>4 years) women (50-70 years). They were randomised to BRT, CRT or control (CON) groups. BRT and CRT involved supervised hack squat and unilateral calf raise training sessions twice weekly for 8 months. Tibial bone properties were assessed using pQCT (Stratec XCT-2000L) scans at 4% and 66% of tibial length. Maximal muscle strength(1RM) was measured monthly to prescribe loads, using undulating periodisation such that they ranged between 20-50% 1RM in BRT and 60-80% 1RM in CRT. Within-group changes were tested with paired t-tests whilst ANCOVA (with baseline scans as a covariate) was used for between-group comparisons.

**Results** 109 women were randomised. 82 completed the study; aged 63.1±3.7 years; BMI 24.5±3.5 $kgm^{-2}$. Mean adherence to training sessions was ~98% in both RT groups. Muscular strength increased in both BRT and CRT (~42%, p<0.001). 76 women (27 BRT, 26 CRT and 23 CON) had good quality pQCT scans. The CRT group showed a significant increase in cortical density at 66% tibia by +0.24% (p=0.01) with greater increase in endosteal circumference (+1.68%, p=0.01) relative to periosteal circumference (+0.76%, p=0.03) resulting in reduced cortical thickness (-1.72%,p=0.002) and area (-0.63%,p=0.04). The changes in cortical density and thickness in CRT were significantly different from those in CON (p=0.05 and p=0.04, respectively; Figure-1). There were no significant changes in BRT. No differences between groups were observed at 4% tibia.

**Conclusion** CRT significantly increased cortical density but reduced cortical thickness relative to control. This seemed to be due to a greater increase in endosteal than periosteal circumference. This tibial shaft widening may be an adaptation to maintain strength in bending. Conventional high load resistance exercise produced structural adaptations at the tibia but ballistic resistance training with lower loads, higher velocity and impact did not.

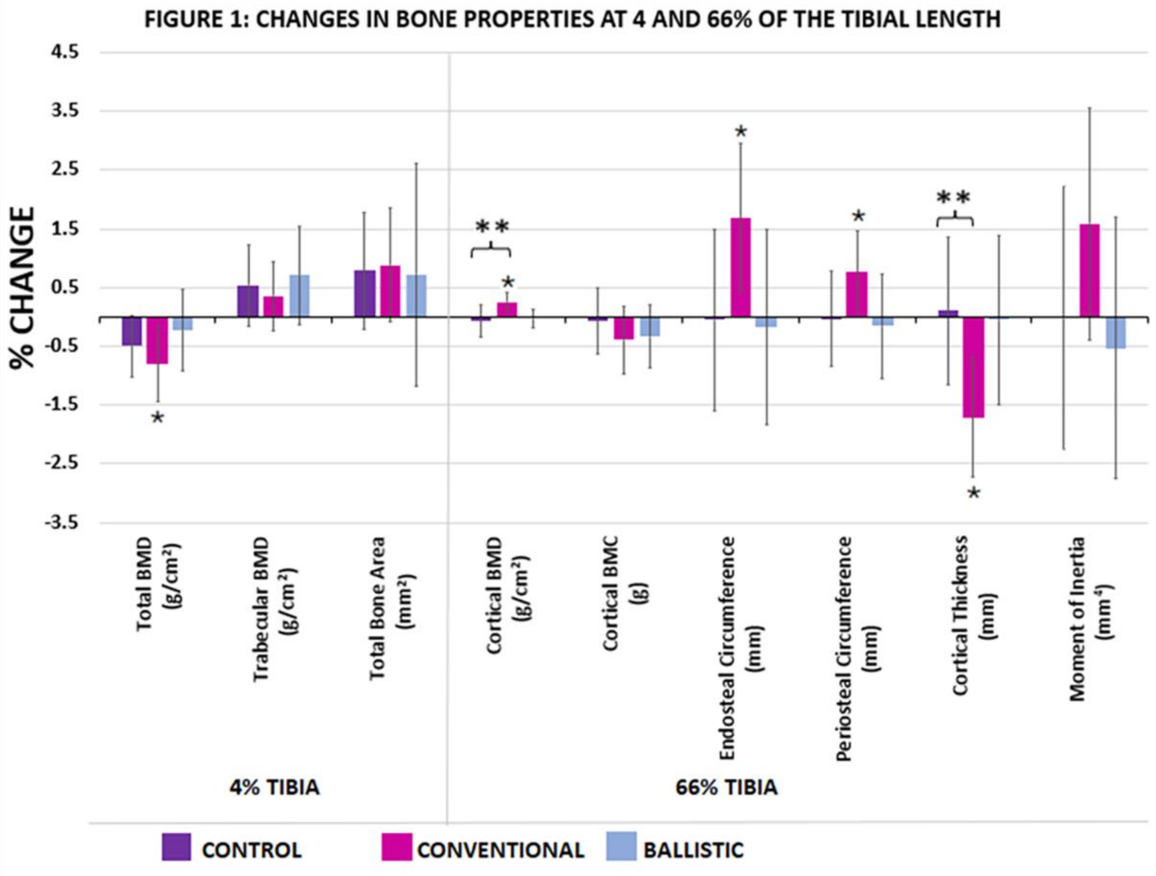

* Significant change from preintervention to postintervention

** Significant difference between groups

Error Bars: 95% CI

### OC6.1

### Integrated transcriptomic and proteomic profiling of responses to clinical and novel bisphosphonates reveal extraskeletal effects and protection against senescence

Jinsen Lu[1], Srinivasa Rao[1], Helen Knowles[1], Eleanor Platt[2], Lucy Frost[2], Tiffany Allen[2], Gayle Marshall[2], Killian Huber[1], Ludwig Bauer[1], Benedikt Kessler[1], Anne Horne[3], Ian Reid[3], James Kirkland[4], Sundeep Khosla[4], Hal Ebetino[5], Graham Russell[1], James Edwards[1]

[1]University of Oxford, Oxford, UK [2]Medicines Discovery Catapult, Manchester, UK [3]University of Auckland, Auckland, New Zealand [4]Mayo Clinic, Rochester, USA [5]BioVinc, Los Angeles, USA

### Abstract

Although bisphosphonates (BPs) are well established for treating bone disorders, many clinical and other studies suggest BPs also have beneficial extra-skeletal effects on cardiovascular disease, respiratory infections, some cancers, neurodegeneration and mortality. However, the mechanisms by which BPs induce such effects are unclear. Using multi-cell screening and studies of BP-treated aged mice and human patients, combined with genomic, transcriptomic, and proteomic analysis, we explored the hypothesis that BPs exert direct effects outside the skeleton to impact multiple aspects of aging.

In multiple cell types, established and novel BPs (incl ZOL, ALN, OX14) increased cell growth at low doses (live cell imaging analysis, 1nM-0.1uM, up to 36%, $p<0.01$). Using fluoro-tagged BPs (ROX-RIS, FAM-ZOL, 0.1uM) to study whether BPs gain access to non-skeletal cells, we showed internalization and co-localization with lysosomal and endosomal organelles (kidney, liver, epithelia, cardiovascular, lung, prostate cells) *in vitro*. *In vivo* spatial transcriptomic analysis revealed differentially expressed genes/pathways in multiple organs of aged (24mth) ZOL-treated (125ug/kg) animals *vs*. untreated, including senescence markers SESN3, CLEC2J and ASB9 ($p<0.05$) with a shift in cellular composition toward those of young (12wk) untreated mice. Similarly, a 5000-plex plasma proteome analysis from osteopenic patients before/after ZOL treatment (36mth) showed significant alterations in ~400 proteins including GTPase regulators, and markers of senescence, autophagy and inflammatory responses. Following this, a mass spectrometry-based cellular thermal shift assay confirmed multiple ZOL-protein interactions (TP-MAP analysis). Reassuringly FPPS was the most BP-bound protein, but we found many similarly stabilized proteins previously unassociated with BP action such as PHB2, FOSL1, CLTB ($p<0.001$). An integrated RNA- and ATAC-seq analysis of BP-treated cells (0.1uM, 96hrs) confirmed alteration of >80 genes including those linked to cell cycle regulation, and senescence (e.g. CCDC68, CDC14B, IGFBP5, PRKACB, $p<0.001$). Functional cellular analyses confirmed pre-treatment of cells with BPs protected against the onset of DNA damage-induced senescence (SA-β-gal, γ-H2AX, x2 fold, $p<0.01$). These multi-omic analyses reveal both novel BP-binding proteins and unidentified transcriptomic/proteomic alterations in non-skeletal cells via which BPs might protect against human aging-linked disorders.

### OC6.2

### Inhibition of epigenetic processes influences osteochondral potential of human mesenchymal progenitors *in vitro*

Isabelle K D Matthews[1], Leah M Wells[1], Jacob A C Keen[1], Ravenska Wagey[2], Isabel Orriss[1], Scott J Roberts[1]

[1]The Royal Veterinary College, London, UK [2]STEMCELL Technologies Inc., Vancouver, Canada

### Abstract

The mechanisms by which the epigenetic status of mesenchymal stromal cells (MSCs) impacts their osteogenic and chondrogenic (osteochondral) differentiation potential remain largely unknown. Gaining an understanding of which aspects of the epigenetic machinery regulate MSC differentiation provides an avenue to better manipulate their differentiation, with implications beyond *in vitro* study such as regenerative medicine, including applications to fracture repair.

A model was developed for osteogenic and chondrogenic differentiation of immortalised human MSCs (ihMSCs) to allow for high throughput drug screening. Chondrogenesis was induced in a high density micromass, osteogenesis in a confluent monolayer, both optimised for 96well-plate culture. A focused, small molecule library was used in a phenotypic screen (glycosaminoglycan deposition/chondrogenesis or mineral deposition/osteogenesis was measured by Alcian Blue or Alizarin Red staining respectively), revealing a number of targets whose inhibition improved the differentiation of ihMSCs. Dose response assays were carried out for compounds targeting these key molecules (as well as some additional targets of interest), measuring matrix parameters, and potential toxicity of the compounds (resazurin-based cell viability assay).

Modulators of GSK3β and methyltransferase PRMT5 were found to promote osteochondral differentiation of ihMSCs, as observed by an increase in both Alizarin Red (GSK3β inhibitor (GSK3βi):control, fold change (fc)=1.47, $p<0.001$; PRMT5i:control, fc=2.04, $p<0.05$) and Alcian Blue (GSK3βi:control, fc=1.98, $p<0.0001$; PRMT5i:control, fc=1.69, $p<0.05$) deposition in osteogenic and chondrogenic differentiation assays respectively. Preliminary investigation using primary human periosteum-derived cells (hPDCs) indicates that these compound hits are translatable to non-immortalised primary populations (GSK3βi:control, fc=1.31, $p<0.001$). Several other compounds were found to specifically promote chondrogenesis, including inhibitors of histone acetyltransferases (HAT) p300/CBP and KAT2A (fc=1.89, $p<0.05$; fc=1.35, $p<0.05$, respectively), as well as PRMT8 (fc=1.35, $p<0.001$), and the DNA-methyltransferase DNMT3β (fc=1.34 $p<0.001$). A dose response assay on all identified compounds found no negative effects on metabolic activity/viability.

These data indicate the importance of epigenetic enzymes in both the osteogenic and chondrogenic differentiation of ihMSCs. In particular, GSK3β and PRMT5 are promising targets for the manipulation of MSC differentiation, with early results indicating translation to primary human mesenchymal progenitors. These data provide new insights into the epigenetic regulation of MSC differentiation, and in turn the potential to further manipulate these processes to achieve optimal tissue formation *in vitro* and *in vivo*.

### Printed Poster Abstracts

### P1

### Participation of agrin expressed by osteocytes in the osseointegration of nanotopographic titanium implants

Maria Paula Oliveira Gomes[1], Letícia Faustino Adolpho[1], Alann Thaffarell Portilho Souza[1], Rayana Longo Bighetti-Trevisan[1], Adriana Luisa Gonçalves Almeida[1], Gustavo Pompermaier Garlet[2], Roland Baron[3], Francesca Gori[3], Adalberto Luiz Rosa[1], Marcio Mateus Beloti[1]

[1]Bone Research Lab, Ribeirão Preto School of Dentistry, University of São Paulo, Ribeirão Preto, Brazil [2]Department of Biological Sciences, School of Dentistry of Bauru, University of São Paulo, Bauru, Brazil [3]Department of Oral Medicine, Infection, and Immunity, Harvard School of Dental Medicine, Harvard University, Boston, USA

### Abstract

Osteocytes are the most abundant cells in bone tissue with key roles in the skeleton function regulating the activities of osteoblasts and osteoclasts, thereby contributing to bone homeostasis and the osseointegration of titanium (Ti) implants. Given the involvement of the extracellular matrix protein agrin in osteoblast differentiation and the osseoinductive potential of nanotopography, the aim of this study was to investigate the participation of agrin expressed by osteocytes in the osseointegration of titanium (Ti) implants with a nanostructured surface (Ti-Nano) generated by conditioning with $H_2SO_4/H_2O_2$. Twelve-week-old genetically modified transgenic mice with conditional deletion of agrin in osteocytes (OcyAgrin$^{-/-}$) and control mice (OcyAgrin$^{+/+}$) were submitted to implant surgery in the oral cavity. Ti-Nano and Ti-Control (without surface conditioning) implants with 0.6 mm diameter and 1.5 mm length were placed between the maxillary first molar to the mid-point on the alveolar crest until behind the incisor. Each mouse received one Ti-Control and one Ti-Nano implant randomly inserted in the right and left side (n = 6 per group). Twenty-one days post-implant surgery, the mice were euthanized, and the maxillae were collected for microcomputed tomography and histological analyses. Data were compared using two-way ANOVA, followed by the Tukey's test and the level of significance was set at $p \leq 0.05$. The conditional deletion of agrin in osteocytes did not affect the osseointegration of Ti-Nano but impaired the osseointegration of Ti-Control. This was evidenced by the reduced bone morphometric parameters in Ti-Control, total volume ($p = 0.013$), bone volume ($p = 0.021$), bone volume fraction ($p = 0.023$), bone surface ($p = 0.015$), bone surface density ($p = 0.018$), trabecular number ($p = 0.019$) and porosity ($p = 0.023$) in OcyAgrin$^{-/-}$ compared with OcyAgrin$^{+/+}$mice, which was not observed in Ti-Nano (p ³ 0.05 for all parameters). Non-decalcified histological sections stained with Stevenel's Blue and Alizarin red showed connective and bone tissue around the implants, without differences between either genotypes or implant surfaces in terms of tissue quality. In conclusion, these findings show that the deletion of agrin in osteocytes disrupts the osseointegration of Ti implants, which might be counterbalanced by the high osteogenic potential of nanostructured Ti surfaces.

**Financial support:** FAPESP (Grant # 2020/14950-4) and CNPq (Grant # 305033/2022-0)

### P2

### Effect of osteoporosis, hypertension and diabetes on bone repair induced by mesenchymal stem cell therapy

Alann Souza, Gileade Freitas, Helena Lopes, Leticia Adolpho, Maria Gomes, Fabiola Oliveira, Adriana Almeida, Márcio Beloti, Adalberto Rosa

Bone Research Lab, Ribeirão Preto School of Dentistry, University of São Paulo, Ribeirão Preto, Brazil

### Abstract

Cell therapy using mesenchymal stem cells (MSCs) has emerged as a promising strategy to repair bone defects when the damage surpasses the regenerative capacity of bone tissue. However, its efficacy has not been proved in concurrent systemic diseases that disturb bone metabolism. The aim of this study was to evaluate whether MSC therapy can repair rat calvarial defects in presence of osteoporosis, hypertension, and diabetes. In Wistar male rats, osteoporosis was induced by orchiectomy (ORX) and diabetes by intraperitoneal injection of streptozotocin (60 mg/kg), and spontaneously hypertensive rats (SHR) were used as a model for hypertension. To mimic pre-existent bone defects, MSCs harvested from healthy rats [$5 \times 10^6$ MSCs in 50 µL of phosphate-buffered saline (PBS)] were directly injected into 5-mm diameter rat calvarial defects, 2 weeks after their creation. Defects injected with 50 µL of PBS were used as control. To evaluate the permanence of MSCs in the calvarial defects, MSCs expressing luciferase were injected and tracked using the Lumina in vivo system. Bone formation was evaluated 4 weeks after injection by microtomography (µCT) and histological analysis. Data were compared by Student's t-test ($p \leq 0.05$). Cell tracking indicated that for all diseases MSCs showed the same pattern of permanence in the defects with the luminescent signal being higher on day 1 after cell injection and presenting a steady decline until day 5, and with a very low signal detected up to day 8. In osteoporosis, the µCT parameters bone volume (BV, $p = 0.003$), bone volume/total volume (BV/TV, $p = 0.002$) and bone surface (BS, $p = 0.005$) were all increased in defects treated with MSCs compared to control without any histological difference in the bone tissue. In hypertension, also BV ($p = 0.005$), BV/TV ($p = 0.005$) and BS ($p = 0.001$) were increased by MSCs without histological difference between MSCs and control defects. In diabetes, there was no difference in any µCT parameter between MSCs and control and the histological analysis either. These results indicate the great potential of MSCs to be used in cell therapy approaches to treat bone defects under osteoporotic and hypertensive conditions as they increased bone formation in these situations, but not in diabetic environment, suggesting that the latter represents a more challenging disorder that demands additional care.

Financial support: FAPESP (#2017/12622-7 and 2018/13290-0) and CNPq (#307698/2021-1)

**P3**

*Withdrawn*

### P4

### Early development of chronic kidney disease has no effect on osteoblastic and adipogenic precursor cell populations in a pre-clinical mouse model

Worachet Promruk[1,2], William Cawthorn[3], Katherine Staines[4], Louise Stephen[1], Colin Farquharson[1]

[1]The Roslin Institute and Royal (Dick) School of Veterinary Studies, University of Edinburgh, Edinburgh, UK [2]Chulabhorn Royal Academy, Bangkok, Thailand [3]Centre for Cardiovascular Science, The Queen's Medical Research Institute, University of Edinburgh, Edinburgh, UK [4]School of Pharmacy & Biomolecular Sciences, University of Brighton, Brighton, UK

### Abstract

Chronic kidney disease (CKD) is a progressive and irreversible disease resulting in the loss of kidney function. This leads to altered calcium and phosphorous homeostasis and bone loss, commonly referred to as renal osteodystrophy (ROD). Bone marrow adipose tissue (BMAT) accumulates in clinical CKD and animal models of this disease, but the mechanisms responsible are unclear. This study evaluated BMAT accumulation, bone phenotypes and mesenchymal stem cells (MSC), osteoblastic (OPC) and adipogenic (APC) precursor cell populations in an early stage of experimental ROD. Eight-week-old male C57BL/6J mice received a diet supplemented with 0.2% adenine for up to 5-weeks to induce CKD. Control mice received the same diet without adenine. Bone phenotypes were evaluated by µCT. The same bones were decalcified and stained with 1% osmium tetroxide and BMAT quantified using µCT. Precursor cell populations in bone marrow were quantified by flow cytometry. CKD development in mice was confirmed by elevated serum levels of blood urea nitrogen and creatinine from 2 weeks of induction. The proximal BMAT located in the metaphysis and distal BMAT started to increase from 3 weeks in CKD mice leading to an increase in total BMAT ($p<0.01$) from 3 week of induction. The trabecular bone phenotype was negatively impacted at 5 weeks of induction where it presented with decreased bone mineral density ($p<0.001$), bone volume fraction ($p<0.01$) and trabecular thickness ($p=0.06$) and number ($p<0.05$). During the early stages (after 1 and 2 weeks of induction) of CKD progression, the MSC population as a percentage of total cells was reduced ($p=0.005$), whereas the OPC and APC populations as a percentage of total cells was unchanged. In conclusion, these results reveal that the expansion of BMAT is not a consequence of altered differentiation of precursor cells in early stages of CKD, but the trans-differentiation of OPCs to the adipocyte lineage cannot yet be ruled out. Ongoing studies will determine the differentiation potential of adipocytes and osteoblasts from their common precursors during CKD progression and investigate the ability of systemic factors that are increased in CKD *e.g.,* PTH, phosphate, FGF23, uremic toxin (indoxyl sulphate) to alter the progression of osteoblast and adipocyte differentiation *in vitro*.

### P5

### Identification of potential drivers of feline head and neck cancer involved in bone invasion

Qaisar Tanveer, Lisa Pang, Colin Farquharson, Gurå Therese Bergkvist

The Roslin Institute, The Royal (Dick) School of Veterinary Studies, The University of Edinburgh, Edinburgh, UK

### Abstract

Feline oral squamous cell carcinomas (FOSCC) are commonly diagnosed tumours and have been proposed as a good model of head and neck tumours in humans. FOSCC are aggressive local tumours with an unfavourable prognosis that invade bone leading to excessive bone resorption. Therefore, the identification of novel biomarkers for bone invasion represents an unmet need to improve clinical outcome. The tumour secretome is a potential source of biomarkers secreted by cancer cells that plays a crucial role in cell proliferation, metastasis, and invasion. Here, we used a single-shot label-free quantification (LFQ) approach based on high performance nanoflow liquid chromatography-mass spectrometry (timsTOF-HT) system to identify protein expression changes in the secretome of bone invasive FOSCC cell lines (SMG and SCCF2) versus bone non-invasive FOSCC cell line (SCCF3). We found that 74 proteins were uniquely secreted by SMG and SCCF2 cell lines compared to 1,120 proteins commonly detected among all FOSCC cell lines. Of these 74 proteins, some proteins including macrophage migration inhibitory factor (MIF), cathepsin-D (CTSD), heterochromatin protein-1 binding protein-3 (HP1BP3), transforming growth factor beta receptor-3 (TGFβR3) and fibrinogen beta-chain (FGB) have established roles in proliferation, migration, and invasion of various cancers while others DSG-1, ECM-1, CLPTM-1, TGM-1, UAF-1 and NAPRT represent putatively novel secreted proteins warranting further investigation. Using Ingenuity Pathway Analysis (IPA) for 1,120 proteins, the most enriched canonical pathways in FOSCC cells included neutrophil degranulation and eukaryotic translation pathways which could promote tumorigenesis and regulate protein processing. IPA also predicted the upstream regulators such as MYC, KRAS, TP53, TGFβ-1, LARP-1, YAP-1, EGFR and HRAS which are implicated in several cancers and bone invasion, demonstrating the validity of our approach. Together, this proteomic analysis demonstrates a robust approach to highlight protein changes in FOSCC that may be further investigated as potential drivers of bone invasion.

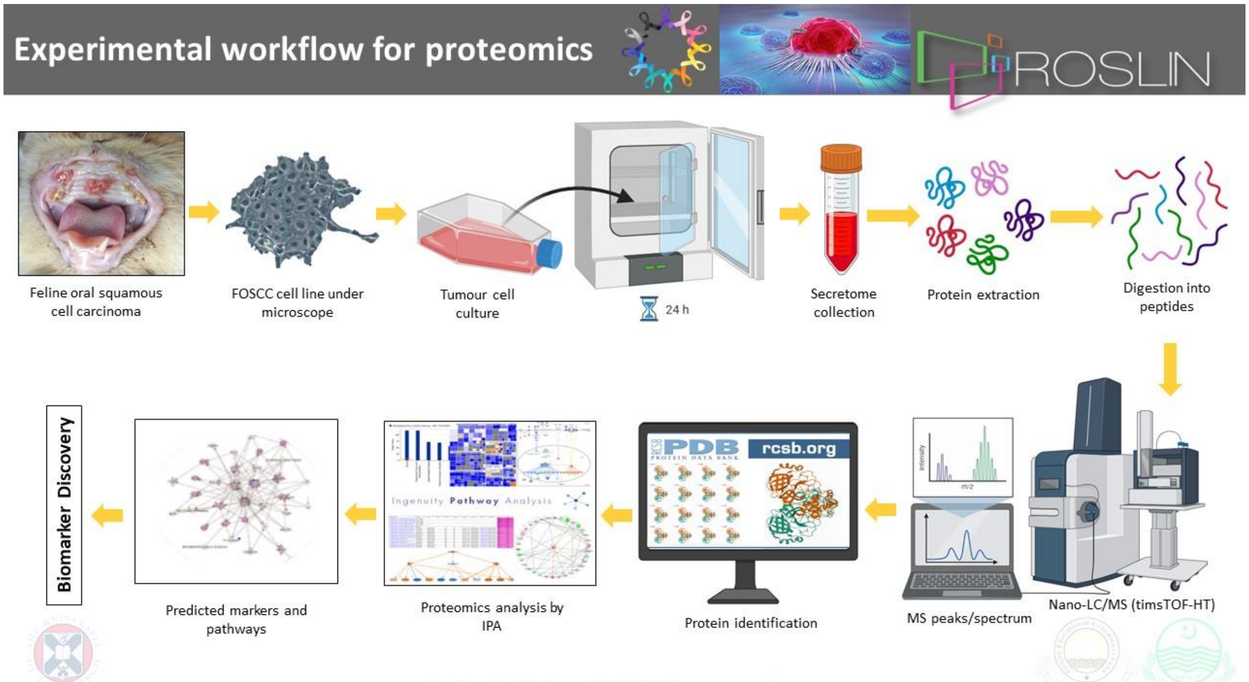

Demichev *et al., Nat Comm.* 2022, 13(1):3944.

**P6**

*Withdrawn*

### P7

### Osteoporosis-induced bone loss is attenuated by jaboticaba peel extract through protection of osteoblastic activity

Letícia Faustino Adolpho[1], Maria Paula Oliveira Gomes[1], Gileade Pereira Freitas[2], Rayana Longo Bighetti-Trevisan[1], Jaqueline Isadora Reis Ramos[1], Gabriela Hernandes Campeoti[1], Guilherme Crepi Zatta[1], Adriana Luisa Gonçalves Almeida[1], Adriana Gadioli Tarone[3], Mario Roberto Marostica-Junior[3], Adalberto Luiz Rosa[1], Marcio Mateus Beloti[1]

[1]Bone Research Lab, Ribeirão Preto School of Dentistry, University of São Paulo, Ribeirão Preto, Brazil [2]Department of Oral and Maxillofacial Surgery, School of Dentistry, Federal University of Goiás, Goiânia, Brazil [3]School of Food Engineering, University of Campinas, Campinas, Brazil

### Abstract

Therapies to prevent osteoporosis is of relevance since it is one of the most common non-communicable human diseases in the world and the most frequent adult bone disorder. The peel of jaboticaba concentrates phenolic acids and anthocyanins, and its extract exhibits potential health benefits such as inhibition of hepatic lipid accumulation and antioxidant and anti-inflammatory properties. As jaboticaba peel extract (JPE) added to the culture medium enhanced the osteogenic potential of mesenchymal stem cells (MSCs) derived from osteoporotic rats, we hypothesized that JPE prevents the development of ovariectomy-induced osteoporosis. Ovariectomized Sprague Dawley rats weighting 250 gwere treated with either JPE (30 mg/kg of body weight) or vehicle/control ( n = 10 per group) for 90 days starting 7 days post-ovariectomy. Then, the femurs were submitted to microcomputed tomography and histological analyses, and the osteoblast and adipocyte differentiation of MSCs derived from bone marrow of these animals were evaluated. The comparisons between JPE and control were done using Student's t-test (p-value ≤ 0.05). JPE attenuated ovariectomy-induced bone loss as evidenced by higher bone volume/total volume (p = 0.050) and trabecular number (p = 0.031), and lower trabecular separation (p = 0.035) and bone marrow adiposity (p = 0.020). The number of osteocytes (p = 0.488) and the osteoclast activity (p = 0.238) were not affected by JPE treatment. Under osteogenic condition, the gene expression of runt-related transcription factor 2 (*Runx2*) (p = 0.035), alkaline phosphatase (*Alp*) (p = 0.001), and osteocalcin (p = 0.001), the protein expression of RUNX2 (p = 0.001) and ALP (p = 0.001), and the extracellular matrix mineralization (p = 0.001) were all higher in MSCs derived from rats treated with JPE compared with MSCs derived from control rats. Under adipogenic condition, the gene expression of peroxisome proliferator-activated receptor-gamma (p = 0.005), adiponectin (p = 0.001), and resistin (p = 0.001) was lower in MSCs derived from rats treated with JPE compared with MSCs derived from control rats, while lipid accumulation was not affected (p = 0.783) by JPE treatment. The protective effect of JPE attenuating the ovariectomy-induced bone loss is due to its ability to prevent the imbalance between osteoblast and adipocyte differentiation of MSCs. These data pave the way for future clinical investigations on the use of JPE as a promising new therapy to prevent osteoporosis.

**Financial support:** FAPESP (Grant # 2017/12622-7) and CNPq (Grant # 305033/2022-0)

### P8

### Three-dimensional changes in tibia cortical geometry that explain variance in strength in a murine osteoporotic bone model

Stamatina Moraiti[1,2], Vee San Cheong[1,2], Enrico Dall'Ara[3,2], Visakan Kadirkamanathan[4,2], Pinaki Bhattacharya[1,2]

[1]Department of Mechanical Engineering, University of Sheffield, Sheffield, UK [2]Insigneo Institute for in silico Medicine, Sheffield, UK [3]Department of Oncology and Metabolism, Sheffield, UK [4]Department of Automatic Control and Systems Engineering, University of Sheffield, Sheffield, UK

### Abstract

### Introduction

Micro-Computed Tomography (µCT) images of rodent long bones are typically analysed using standard morphometric parameters (1) that do not fully capture three-dimensional (3D) variations in geometry (2). This study identifies 3D geometric modes that explain the variation in bone strength in an osteoporotic bone model using the Partial Least Square (PLS) method.

### Methods

Eleven 14-week-old C57BL/6 mice underwent ovariectomy; six received mechanical loading and Parathyroid hormone (PTH 1-34) injections from weeks 18 to 22, the rest remained untreated. In vivo µCT scans (10.4 µm/voxel) were taken every two weeks (3). Images from weeks 18 and 24 were selected, including slices spanning 80% of tibial length L (4). These were used to perform micro-Finite Element analysis that predicted tibia strength under compression (5). In the present study, the images were further cropped to solely include the tibia cortex (65%L) and exclude non-topological equivalent ends, rigidly aligned to a reference image, binarized and geometry corrected, and lastly mapped using elastic registration to the reference. This produced a displacement field capturing surface differences between mice. PLS determined modes of displacement maximally correlated with tibia strength. Tests determined whether the median change in the scores in a group of a PLS mode between weeks 18 and 24 was statistically significantly different from zero ($p<0.05$). A positive result in both groups implied that the mode described growth; a positive result in the treatment group implied a treatment-related mode.

### Results

The reference surfaces were discretised using ~500k nodes. Figure 1 plots the mode vectors scaled by the median score change considering both groups together (mode 1, growth-related) and treated group (modes 2–4, treatment-related). Mode 1 captures a global change in size, mode 2 a global change in curvature and area and modes 3 and 4 local changes in shape. Altogether, these modes describe 92% and 98% of the total variance in geometry and strength, respectively.

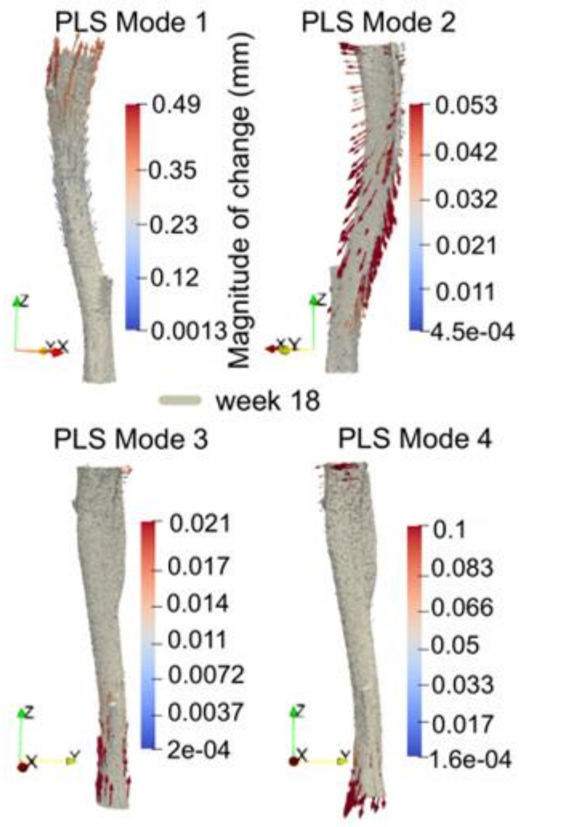

*Figure 1: 3D geometric changes between week 18 and 24. Scaled and coloured by the magnitude of change, shown on top of the mean mode-specific bone profile at week 18.*

**Conclusions**

This study identified new 3D features that capture mouse tibia adaptation to treatment and concurrently explain more than 92% of variance in geometry and strength.

**Acknowledgments**

Funded by the UKRI grants EP/S032940/1 and EP/V050346/1 and the NC3Rs grant.

**References**

1. Bouxsein, M.L. et al, J Bone Miner Res, 25:1468-1486, 2010
2. Moraiti, S. et al, 28th ESB Congress, Maastricht, 2023
3. Roberts, B.C. et al, Sci Rep, 10:1-16, 2020
4. Cheong, V.S. et al, Acta Biomater, 116:302-317, 2020
5. Roberts, B.C. et al, J. Orthop. Res, 2023

### P9

### The pre-osteoarthritic tibial subchondral plate is characterised by excessive articular calcified cartilage in the STR/Ort mouse model of osteoarthritis

Lucinda Evans[1,2], Aikta Sharma[3], Alissa Parmenter[3], Sebastian Marussi[3], Joseph Brunet[3], Catherine Disney[4], Alisha Bhatt[3], Kamel Madi[5], Paul Tafforeau[6], Andrew Bodey[4], Peter Lee[3], Andrew Pitsillides[1], Katherine Staines[2]

[1]Royal Veterinary College, London, UK [2]University of Brighton, London, UK [3]University College London, London, UK [4]Diamond Light Source, Oxford, UK [5]3Dmagination, Oxford, UK [6]European Synchrotron Radiation Facility, Grenoble, France

### Abstract

**Objectives:**

Condylar microarchitecture likely contributes to articular region fatigue vulnerability, and future osteoarthritis initiation. We aimed to anatomically characterise and compare the tibial subchondral plates (SCP) of osteoarthritic (OA, STR/Ort) and healthy-ageing (CBA) mouse models, in high-resolution, at a pre-OA timepoint.

**Methods:**

Fresh-frozen, intact 10-week-old mouse knees (n=3 per strain) were synchrotron-CT scanned at Diamond Light Source (1.625µm voxel, MG30897-1) or the European Synchrotron Facility (1.450µm, LS-3124). Sample motion was prevented by 2N load application. Scans were reconstructed in-house, regions of interest segmented, and thickness analysed in Avizo. Statistics were performed in SPSS.

**Results:**

Linear mixed model analysis identified significant strain/tissue interaction ($p<0.001$), confirming that thickness of components within SCP (medial or lateral articular calcified cartilage (ACC) or subchondral bone (SCB)) vary between mouse strains.

STR/Ort and CBA SCPs (ACC and SCB combined) did not differ significantly in overall thickness ($p=0.611$) at this 10-week 'pre-OA' stage, in which no bone sclerosis is yet apparent. Pronounced differences were observed, however, in SCP composition. Medial and lateral tibial ACC are both thicker overall in STR/Ort mice ($p=0.014$ and 0.050 respectively).

CBA mice were confirmed by post-hoc pairwise testing to have no significant difference between ACC and SCB thickness on either medial or lateral tibial condyle (all $p>0.1$), indicating that these layers contribute equally to healthy SCP. Likewise, CBA medial ACC was similar to lateral ACC (116µm±10, $p=0.151$) and CBA medial SCB was similar to lateral SCB (130µm±13, $p=0.138$), signifying condylar symmetry within healthy knees.

In stark contrast, STR/Ort ACC is thicker than SCB in both medial ($p=0.004$) and lateral ($p<0.001$) condyles; ACC contributes disproportionately to the pre-osteoarthritic subchondral plate. STR/Ort tibial condyles are also asymmetrical: medial ACC is thicker than lateral ACC (152µm±9 vs 130µm±13, p=0.046).

**Conclusions:**

Our findings show that bony sclerosis of the tibia, well-documented in late-stage OA in both humans and our STR/Ort model, is preceded by pathological ACC overgrowth. This is particularly exaggerated on the medial condyle, where STR/Ort OA vulnerability is greatest.

It is plausible that this excessive ACC formation is the earliest manifestation of an endochondral ossification defect that will later result in osteoarthritic sclerosis, osteophytes – and indeed, articular lesions.

As calcified cartilage is not resolvable in standard desktop micro-CT, these findings additionally highlight the value of our high-resolution sCT scanning method to facilitate step-changes in our understanding of osteoarthritis pathobiology.

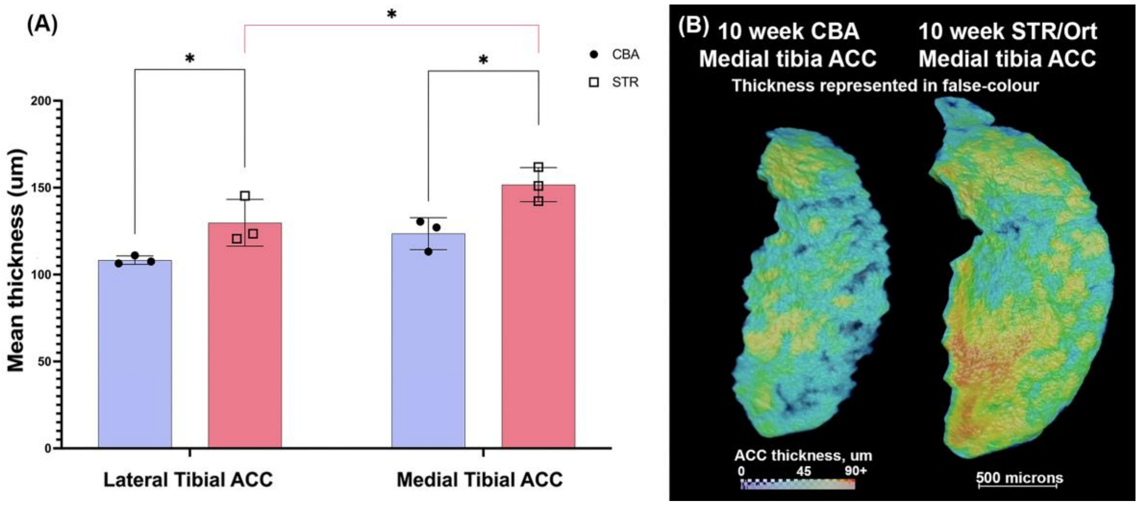

### P10

### Rapid assessment of osteogenesis by using a Murine Tibial Periosteal Ossification model

Emma Steijvers[1], Weixin Zhang[2], Yitian Zhang[1], James Triffitt[3], Graham Russell[3,4], David Deganello[1], Xu Cao[2], Zhidao Xia[1]

[1]Swansea University, Swansea, UK [2]Department of Orthopaedics, Johns Hopkins Medical School, Baltimore, USA [3]Oxford University, Oxford, UK [4]4. Mellanby Centre, Sheffield University, Sheffield, UK

### Abstract

Biomaterials have been widely used as orthopaedic implants and bone graft substitutes. Extensive assessments of their osteogenic potential are required before regulatory approval; however, it takes months using current fracture or bone defect healing models. This study aims to develop a rapid osteogenic assessment using a murine tibial periosteal ossification model to evaluate biomaterial bone formation/remodelling potential within 2-4 weeks. A novel hydroxyapatite/aragonite (HAA) biomaterial was implanted into C57BL/6 mice juxtaskeletally between the tibia and tibialis anterior muscle. Rapid intramembranous bone formation was observed at 14 days, followed by bone remodelling and a new layer of cortical bone formation at 28 days after implantation. The osteogenic potential of hydroxyapatite/aragonite was further confirmed by implanting into rat and minipig femoral cancellous and cortical bone defect models. We conclude that murine tibial periosteal ossification is a reliable method for rapid assessment of osteogenic potential of biomaterials, and the novel hydroxyapatite/aragonite composition is an osteogenic and biodegradable material.

### P11

### Characterisation of nanoscale strain patterns in physiologically-loaded osteoarthritis-prone joints reveals articular foci of high compression

Aikta Sharma[1,2], Lucinda Evans[3,4], Alissa Parmenter[1,2], Joseph Brunet[1], Sebastian Marussi[1,2], Kamel Madi[5], Paul Tafforeau[6], Andrew Pitsillides[4], Peter Lee[1,2], Katherine Staines[3]

[1]University College London, London, UK [2]Research Complex at Harwell, Oxford, UK [3]University of Brighton, Brighton, UK [4]Royal Veterinary College, London, UK [5]3Dmagination, Oxford, UK [6]European Synchrotron Radiation Facility, Grenoble, France

### Abstract

Osteoarthritis (OA) is a progressive, age-related degenerative joint disease with known mechanical aetiological contribution. OA – hallmarked by articular cartilage loss, subchondral bone thickening and osteophytogenesis – emerges in healthy joints where mechanical loads are essential for maintaining biomechanical competence. The distinct mechanics that preserve healthy joint ageing, versus those that drive OA onset and progression, however, remain ill-defined. Herein, we describe an analytical pipeline for hierarchical characterisation and measurement of 3D nanoscale biomechanical strains across the whole tibial epiphysis in a murine OA model and normal intact joints, under physiological load.

Whole hindlimbs from male OA-prone STR/Ort and healthy parental strain CBA mice (N=3) at ages prior to OA onset (10-weeks) and advanced OA (40-weeks) were mounted on a loading rig (Deben CT500) in bespoke holders for in-situ physiological compression. A series of high-resolution synchrotron computed tomography (sCT) images (1.45 µm/voxel) of each joint were acquired at the European Synchrotron Radiation Facility, Grenoble (BM05-beamline) following step-wise displacement-controlled loading. Tomographic datasets were reconstructed using in-house software, followed by 3D analyses of strain magnitudes and patterns by global digital volume correlation (DVC, involving segmentation and tetrahedral mesh generation) across all anatomical zones in reconstructed datasets in Avizo 3D (XDVC module).

3D displacements and strain fields were quantified across whole tibial epiphyses with a nanoscale displacement accuracy of <90nm (<0.06voxels) and a strain precision (<350µstrain, 0.035%) across articular calcified cartilage, subchondral and trabecular bone, and growth plate bridges. Intriguingly, load-induced compressive strains were effectively transferred between articular-to-metaphyseal regions in all joints. In STR/Orts, however, additional foci of high magnitude compression were observed in articular zones, which were completely absent in all healthy CBA joints. Global average strain quantification revealed higher tensile (P=0.0001) and compressive strain (P=0.0089) in STR/Ort versus CBA at 10-weeks. At 40-weeks, tensile strains were higher in STR/Ort versus CBA (P=0.0107) while compressive strains were comparable.

sCT imaging of in-situ loaded joints with DVC has enabled visualisation and quantification of nanoscale strains across the intact murine tibial epiphysis. Our data indicate that joint microarchitecture differs in OA-prone joints, leading to strain localisation within articular regions. In healthy joints however, the strain is efficiently transferred to remote epiphyseal regions. Thus, we speculate that these strain patterns at pre-OA stages may predetermine pathological architectural changes both before and with advancing OA to ultimately induce joint failure. Further quantification of localised, region-specific strains will aid in deciphering these pathological mechanisms.

### P12

### Bone formation strategies using hydrogel encapsulation of lineage-specified pluripotent stem cells

Jiadong Liu[1], Hongqiang Yu[1], Ewa Kania[1], Suzanne Eldridge[2], Francesco Dell'Accio[2], Eileen Gentleman[1], Agamemnon Grigoriadis[1]

[1]King's College London, London, UK [2]Queen Mary University of London, London, UK

### Abstract

Efficient repair of bone defects following injury, dental procedures or cancer treatment is an important clinical problem. Tissue engineering is regarded as an optimal approach to regenerate tissue defects by combining three key factors for tissue repair, including cells, biomaterials and stimulatory factors. In this study, we have used a pluripotent stem cell (PSC) differentiation approach combined with 3D hydrogel encapsulation to investigate the osteogenic potential of Growth Differentiation Factor (GDF)5.

We have derived a population of precursors with chondro/osteo potential from mouse embryonic stem cells (mESCs) cultured in defined media through a step-wise protocol involving primitive streak/mesoderm specification and FGF2-dependent mesoderm enrichment. Addition of GDF5 to the mESC-derived mesodermal precursors induced a chondro/osteogenic population that can produce hypertrophic chondrocytes and osteoblasts as confirmed by histology and increased expression of chondro/osteo gene markers (*Sox9*, *Col2*, *Col10*, *Runx2*, *Osx*). This GDF5-induced population can be produced either in long-term monolayer culture or as spheroids that display self-forming 3D structures with osteoblasts surrounding chondrocytes in the multilayered nodules/spheroids. Interestingly, replating differentiated chondro/osteo nodules can enrich the osteogenic potential at the expense of the chondrocyte phenotype.

For 3D hydrogel encapsulation studies, GDF5-treated dissociated cells showed good survival and proliferated into colonies with cartilage/bone matrix deposition in a hyaluronic acid-based hydrogel formed via a Michael addition. qPCR analysis confirmed that encapsulated cells are undergoing further hypertrophy and osteogenic differentiation with increased expression of hypertrophic chondrocyte (*Col10*) and osteoblastic (*Ocn*) markers. The influence of mechanical cues on 3D-encapsulated cell differentiation was investigated by tuning hydrogel stiffness changed by varying polymer concentration, and preliminary histological analysis suggested that a softer gel (~1kPa) could provide a better environment for osteogenic differentiation of GDF5-induced chondro/osteo population, with enhanced alkaline phosphatase and osteocalcin staining.

To investigate the *in vivo* potential of GDF5-induced cells, we first performed xenograft studies using the chick chorioallantoic membrane (CAM) assay, which showed that bone-like tissues developed from grafted GDF5-treated cells. Finally, *in vivo* mouse intramuscular transplantation of dissociated GDF5-induced chondro/osteo nodules encapsulated in a hyaluronic acid hydrogel showed formation of mineralised bone-like tissues as demonstrated by microCT and histological analyses. Overall, these results show that GDF5-induced PSCs can produce a chondro/osteogenic population that can undergo an endochondral-like ossification process *in vitro* and *in vivo*, and we are currently optimising the 2D/3D induction protocol to investigate the potential to repair critical size defects *in vivo*.

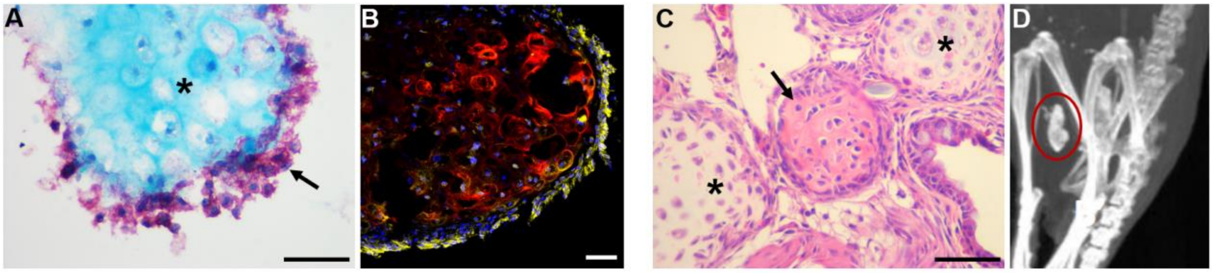

(A) Alkaline phosphatase (arrow) and Alcian blue (*) co-staining of GDF5-induced cells *in vitro*. (B) Collagen X (red) and osteocalcin (yellow) immunofluorescence co-staining of GDF5-induced chondro/osteo nodules. (C) H&E staining of GDF5-induced nodules after grafting onto a CAM showing hypertrophic chondrocyte (*) and bone (arrow) tissue formation. (D) MicroCT image of mineralised bone-like tissue formed from i/m grafting of GDF5-induced PSCs encapsulated in a hyaluronic acid hydrogel. Scale bar: 50μm.

### P13

### Translating high resolution bone scans to 3D bio-printable models

Lucy Dascombe[1], Ronak Janani[2], Chris Sammon[2], Tim Nichol[1], Christine Le Maitre[3], Nicola Aberdein[1]

[1]Biomolecular Science Research Centre, Sheffield Hallam University, Sheffield, UK [2]Materials and Engineering Research Institute, Sheffield Hallam University, Sheffield, UK [3]Division of clinical medicine, School of medicine and population health, University of Sheffield, Sheffield, UK

### Abstract

Objective: This study aims to translate high resolution micro-computational tomography (Micro-CT) imaging of *ex-vivo* murine bone samples to 3D bio-printable rendered standard tessellation language (STL) files through computed assisted design (CAD) software. Micro-CT allows non-invasive visualsation of inorganic dense material, which can be quantitatively analysed for morphometry, density, and material distribution. By characterising the micro-architecture of bone, a physiologically relevant 3D model of trabecular (Tb) and cortical (Cb) morphometry can be produced. Thereby generating a biomimic 3D model in an appropriate resolution for 3D bioprinting with polymer-based bio-inks for use of *in vitro* biomedical research.

Methodology: *Ex-vivo* murine tibia, femur and vertebrae were imaged using Micro-CT with a voxel size of 9 µm and reconstructed to determine the morphometry of Tb and Cb. A volume of interest was processed and exported as a binary STL file. Utilising Autodesk® CAD software, Meshmixer and Fusion 360, the STL model was rendered *in-silico* to a translatable printable resolution in the mm range. Models were exported as STL files prior to extrusion based bioprinting (BioX, Cellink) with B-gel (A. Thorpe, 2016) encapsulating pre-osteoblast cells (MC3T3-E1) and utilising a thermo-reversible microparticle support slurry. A variety of printing parameters were used to determine optimal printing fidelity.

Results: Multi-species analysis of Cb and Tb highlighted significant differences in morphometric outputs for both mouse and rat, including Tb. Thickness (mm) of the femur (0.06 mm and 0.09mm, respectively). Extrusion-based bioprinting has a limited resolution capability due to the nozzle inner diameter of 0.403 mm. Morphometric output ratios were informed for the 3D model with *in-silico* rendering required to translate the *ex-vivo* bone in the µm range, to a 3D printable model in the mm range. 3D CAD models underwent rendering in XYZ orientation in relation to morphometric outputs, followed by reduction and repair of tessellation via smoothing to ensure a manifold structure and reduced file size. Removal of architecture that would not allow a stable 3D construct were removed. Future work is to compare the fidelity of the bio-printed CAD rendered bone model to the *ex-vivo* bone via Micro-CT to assess accuracy in reproducibility.

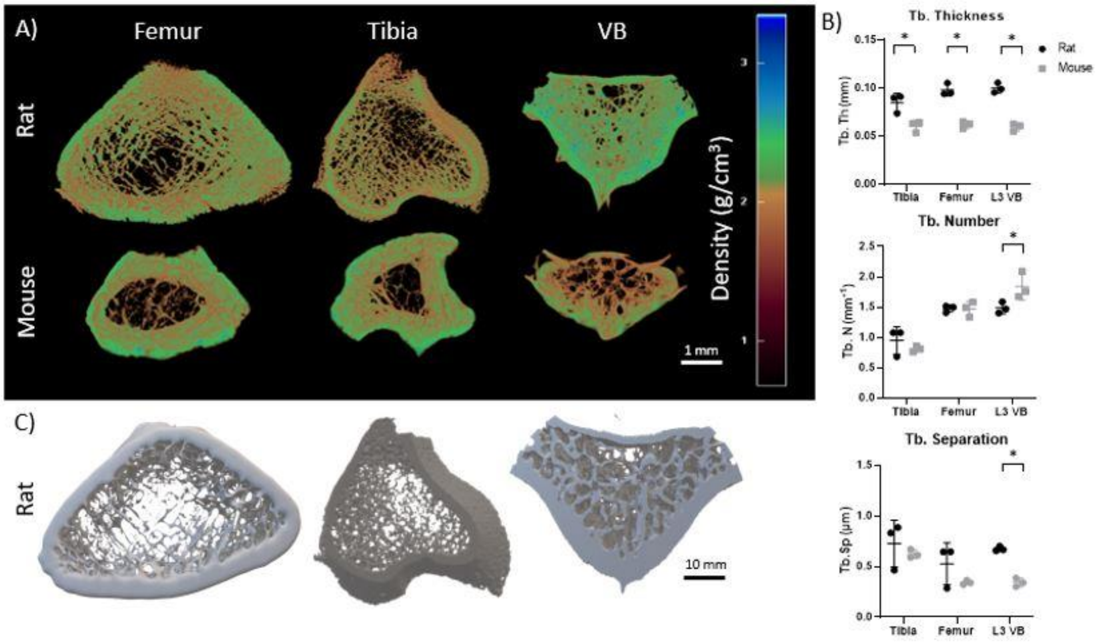

*Figure 1. Translation of ex-vivo male murine bones into 3D printable models. A) Micro-CT VOI of rat and mouse femur, tibia and VB VOI used for morphometry analysis. B) Tb Thickness (mm), number and separation. C) CAD rendered rat femur, tibia, and VB for 3D bioprinting. Representative images shown, N=3.*

Significance: *In vitro* 3D bone models typically do not encompass the complex architecture of bone, including both cortical and trabecular morphology. Using micro-CT as a method of 3-dimensional quantification will allow for *ex-vivo* bone models to be rendered and translated for comprehensive biomedical testing in line with NC3Rs.

Thorpe, A., Creasey, S., Sammon, C., Le Maitre, C. (2016)

### P14

### Gas-delivery perfluorocarbon nanodroplets affect the growth and differentiation of osteoblasts and osteoclasts

Kirsten O'Brien[1], Robin Rumney[2], Christopher Campbell[3], Helen Knowles[3], Dario Carugo[3], Eleanor Stride[3], Nicholas Evans[1]

[1]University of Southampton, Southampton, UK [2]Portsmouth University, Portsmouth, UK [3]University of Oxford, Oxford, UK

### Abstract

Hypoxia is associated with common bone diseases and complications. Current clinical interventions may be invasive or suboptimal. Perfluorocarbons are capable of dissolving large amounts of oxygen but are insoluble in blood; they must be stabilised by a monolayer of amphipathic molecules (such as phospholipids) to form a nanodroplet. This work tests the hypothesis that nanodroplets do not affect osteoblast or osteoclast behaviour at low therapeutically relevant concentrations.

Nanodroplets were made with DSPC and PEG(40)s in PBS with perfluoropentane using sonication. To determine oxygen delivery to cells, the relative concentration of HIF-1α in lysates of SAOS-2 cells grown in hypoxia were compared to those grown at normoxia in the presence and absence of nanodroplets was measured by western blot. Bone marrow stromal cells (BMSCs) and peripheral blood mononuclear cells (PBMCs) from human donors were seeded at 2.5 × 104 and 7.6 × 105 cells/cm2 respectively. The cells were then treated with a dilution of nanodroplets (0, 0.01, 0.1 and 1% v/v) and placed in either normoxia or hypoxia. Bone cell activity was quantified using alkaline phosphatase (ALP) expression, alizarin red staining and manual cell counting.

The HIF-1α concentration in the presence of air-saturated nanodroplets was found to be 71.8 % ± 10.3 compared to the absence of droplets in hypoxia (n = 3, p = 0.0414). This was not observed in the oxygen- (113.2% ± 18.6, n=3, P > 0.05) and nitrogen-saturated (95.8% ± 21.1, n=2) groups. ALP was found to decrease significantly after 14 days of treatment (87.6% ± 3.1). However, alizarin red staining for mineralisation was found to significantly increase when treated with nanodroplets and lipid only under normoxic osteogenic conditions (8-fold and 3-fold increase respectively). Osteoclast number was found to decrease in normoxia with treatment (p = 0.0002). In hypoxia, average osteoclast size decreased (p = 0.0001).

This work indicates that nanodroplets may be suitable for promoting osteogenic differentiation and may be suitable as a gas-delivery vehicle in bone repair. Future work will assess the effect of nanodroplets on osteoclastogenesis and osteoclast activity.

### P15

### Magnetic hydrogels for bone tissue engineering

Emma Kelly, Catherine Berry, Manuel Salmeron-Sanchez

University of Glasgow, Glasgow, UK

### Abstract

**Introduction:** Bone injuries pose a significant healthcare burden due to their prevalence and the necessity for effective regenerative therapies. With over 4 million bone grafts performed annually, innovative solutions are imperative to address the diverse range of issues affecting bone regeneration. Magnetic fields have been applied to stimulate bone healing, through the mechanism of mechanotransduction, however the effects of magnetic fields on the cellular and molecular mechanisms require better understanding This study introduces a magnetic hydrogel model as a potential therapeutic approach for bone tissue engineering and a model to allow investigation of magnetic fields on mesenchymal stem cells (MSCs).

**Materials and Methods:** Our magnetic hydrogel comprises a gelatin matrix functionalized with a photocrosslinkable methacryloyl group and incorporated iron oxide magnetic nanoparticles. Previous characterisation confirmed its biocompatibility with MSCs. The mechanical properties, pore size, cell viability, and water contact angle were optimized to create an ideal microenvironment for cell encapsulation. MSCs were encapsulated within the magnetic hydrogel and exposed to a static magnetic field (370mT) to promote osteogenesis.

**Results and Discussion:** Analysis at day 14 revealed significant upregulation of early osteogenic markers, ALP and Runx2, indicating accelerated MSC differentiation towards osteogenic lineages. Additionally, the late osteogenic marker Osteopontin was upregulated, suggesting continued progression of osteogenic differentiation. Surprisingly, stemness markers Alcam and Nestin were also elevated at day 14, hinting at potential self-renewal of stem cells within the hydrogel. The magnetic hydrogel demonstrated the ability to promote osteogenesis in bone tissue engineering. Given the significant number of bone grafts performed annually, this approach offers a promising alternative to traditional methods. Further research is needed to examine cellular and molecular changes to the cells in response to a magnetic field.

### P16

### Floating on fat - How joints work and why they develop osteoarthritis

Michael Beverly[1], David Murray[2]

[1]Botnar Research Centre, NOC, Oxford, UK [2]OOEC, Botnar Research Centre, Oxford, UK

### Abstract

#### Background

Joints experience massive forces but subchondral circulation was never studied during activity.

Intraosseous pressure (IOP) poorly understood. Bone fat is semi-liquid at body temperature. Subchondral adipocytes, capillaries and trabeculae are delicate and flexible yet tolerate huge loads.

T2 MRI scans show subchondral vascular marks, lost in osteoarthritis (OA) Fig1. Anatomy and histology support hydraulic pressure load-bearing.

#### Objectives

To demonstrate:
IOP varies and is not a constant for the whole bone but reflects systemic blood pressure. Individual IOPs have a proportionate pulse volume (PV). Subtracting proximal arterial occlusion (IOPa) from proximal venous occlusion (IOPv), gives needle tip perfusion 'bandwidth'. Subchondral IOP is increased by loading, far above perfusion pressures. Steroid manipulation of intraosseous fat volume alters perfusion. Sub-cortical choke valves contain raised hydraulic pressure during load transfer. MRI scans have water bright marks, lost in early osteoarthritis Fig2. OA is a vasculo-mechanical disease.

#### Methods

IOP perfusion and loading studies in vitro with a perfused calf foot model (x188), in vivo in the rabbit (x42) and in man (x7), with and without vascular occlusion. Steroid-treated model (x12) with IOP and angiograms compared to untreated control. Sixty pairs of upper tibial MRI scan vascular marks compared with Kellgren-Lawrence (K-L) plain x-ray score. Histology - calf, rabbit and man, normal and OA.

#### Results

IOP is proportional to BP $p<0.0005$
IOP is proportional to pulse volume $p<0.001$, correlation $r^2=0.9089$
IOP rises with proximal venous occlusion $p<0.012$
IOP falls with proximal arterial occlusion $p<0.0001$
Subchondral IOP rise with one body weight is 53% $p<0.0002$
Steroid model loses bone fat giving secondary hyperdynamic flow on IOP, micro-angiograms confirm.
Loss in vascular MRI marks with increasing K-L grade of OA $p<0.002$.
Histology demonstrates same MRI vessels in subchondral plane and sub-cortical choke valves.

**Conclusions**

Combinations of IOP measurement with proximal vascular occlusion and loading allow exploration of subchondral weight-bearing physiology. Intraosseous fat volume manipulation with steroids affects IOP. Specialised bone lipids and vascular anatomy allow hydraulic pressure load transmission. A loss in MRI subchondral vascularity is associated with increasing OA. This new field of subchondral physiology holds the key to an understanding of osteoarthritis and other bone pathology and the hope of better treatment.

Fig1 Vascular marks on MRI

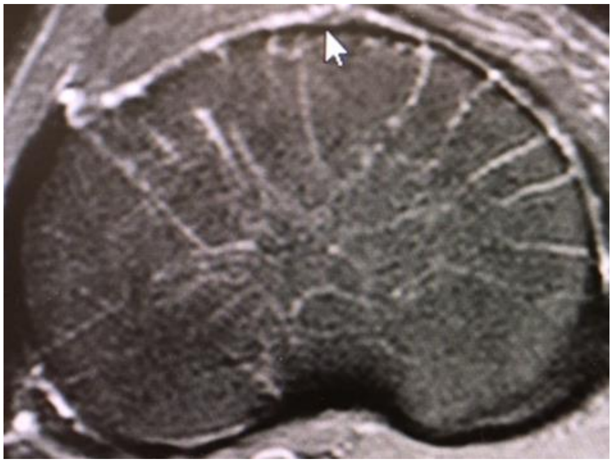

Fig2 Loss of marks with increasing OA

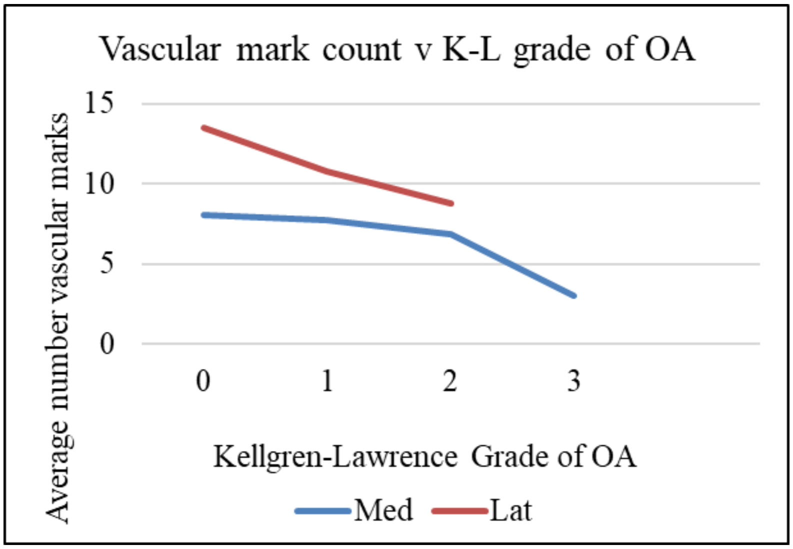

**P17**

*Withdrawn*

### P18

### Investigating the association of Galectin-1 and Kizuna expression on fracture risk in thoroughbred racehorses

Amy Ross, Esther Palomino-Lago, Ellison Lumsden, Debbie Guest

The Royal Veterinary College, Hatfield, UK

**Abstract**

Bone overloading fractures are the primary cause of euthanasia in racehorses. A polygenic risk scoring system using fibroblasts from 44 Thoroughbreds, deemed high and low genetic risk of fracture, was utilized to generate induced pluripotent stem cells (iPSCs), which were then differentiated into osteoblasts. RNA sequencing was subsequently performed revealing 112 differentially expressed genes (DEGs), 42 of which had not been associated with bone. Of these 42 genes, 37 are expressed in equine bone tissue and 22 in human osteosarcoma (Saos2) cells. Four genes with previously published roles in bone, were also expressed in Soas2. One of which was *LGALS1* which has been associated with enhanced osteogenesis, modulating osteoblastic proliferation and differentiation. It was expressed more highly (2.8 fold) in low-risk equine osteoblasts and selected for further study alongside Kizuna Centrosomal Protein (*KIZ*). KIZ provides stability prior to spindle formation but does not have a published role in bone. However, it is expressed more highly (2.3 fold) in high-risk equine osteoblasts.

*LGALS1* and *KIZ* were overexpressed via stable transfection and knocked down by viral integration of a short hairpin RNA. ELISA assays and qPCR confirmed successful modulation of *LGALS1*. Overexpression resulted in minimal differences in alkaline phosphatase (ALP) activity, osteoblast, and osteocyte gene expression under both osteogenic and basal conditions. It did, however, result in a 17% decrease in cell viability in basal conditions ($p<0.05$) and decreased mineralisation by 90% under osteogenic conditions ($p<0.01$).

Knocking-down *LGALS1* in Saos2 cells reduced cell viability by 53% in basal conditions ($p<0.001$), but increased matrix mineralization by 95% ($p<0.001$) and increased ALP activity by 97% after 7 days of osteogenic culture ($p<0.01$). There were significant changes in the expression of osteoblast and osteocyte genes with significant increases in *MEPE*, *SPP1*, *SP7*, *PHEX* and *SOST* under basal conditions, whereas after 21 days of osteogenic culture there were significant increases in *MEPE* and *RUNX2* and significant decreases in *COL1A1*, *DMP1*, *SP7* ($p<0.05$).

These changes in gene expression, cell proliferation, and osteogenic differentiation associated with *LGALS1* modulation suggest that decreased *LGALS1* expression in osteoblasts derived high-risk horses may contribute to an increased risk of bone overloading fractures in Thoroughbred racehorses.

KIZ could not be successfully modulated in Soas2 cells, supporting predictions that it may be essential in osteogenic cancer cell types. Differential expression in high and low risk osteoblasts could contribute to osteoblast viability and fracture risk. Proof of concept assays are under way to confirm this.

### P19

### The effect of the secretome of mesenchymal stem cells primed by a microtopographic surface on osteoblast proliferation, migration and differentiation

Lucas Arruda[1], Robson Calixto[1], Fabíola Oliveira[1], Jessica Frith[2], Márcio Beloti[1], Adalberto Rosa[1]

[1]Bone Research Lab, Ribeirão Preto School of Dentistry, University of São Paulo, Ribeirão Preto, Brazil [2]Materials Science and Engineering, Monash University, Clayton, Australia

### Abstract

Mesenchymal stem cells (MSCs) are the first choice in cell therapy for bone regeneration where their paracrine effects on osteoblasts are thought to drive regenerative processes. Currently, there is substantial interest in improving this effect by priming of the MSCs with technologies using bioengineered substrates. The aim of this study was to evaluate the effect of the secretome of MSCs primed by culturing on a microtopographic surface on osteoblast proliferation, migration, and differentiation. MSCs were cultured on a tissue culture plastic surface of micropillars with 5 µm width/spacing and 5 µm height in growth medium that is alpha-MEM supplemented with 10% foetal bovine serum (FBS) and antibiotics. Upon reaching confluence, they were cultured for a further 4 hours in FBS-free medium to obtain conditioned medium containing their secretome (pCM). MSCs cultured on standard culture surfaces were used as control (CM). Osteoblasts harvested from newborn rat calvaria were cultured in osteogenic medium (growth medium supplemented with 5 mg/mL ascorbic acid and 7 mmol/L of beta-glycerophosphate) with either pCM or CM in the ratio of 1:1 on the last 48 hours before assays were carried out. Cell counting (n = 5) was performed at days 3, 7 and 10. Cell migration (n = 4) was evaluated after scratching confluent cultures with a 100 µL pipette tip and measuring the healing area at 0, 6, 12 and 24 hours. Osteoblast differentiation was evaluated at day 7 by gene expression (n = 4) of the osteoblast markers runt-related transcription factor 2, osterix, alkaline phosphatase, bone sialoprotein, osteopontin, and osteocalcin, and alkaline phosphatase activity (n = 5). Data were compared by either Student's t-test or ANOVA followed by Tukey's test ($p \leq 0.05$). Osteoblast proliferation increased from 3 to 10 days ($p = 0.001$) without difference between cells cultured with either pCM or CM in all evaluated time points. Osteoblast migration was higher in cultures treated with pCM ($p = 0.002$) and the scratches were fully closed at 24 hours. Osteoblast differentiation was increased by pCM as revealed by the higher gene expression of all osteoblast markers ($p = 0.001$ for all of them), despite ALP activity not being affected. As the paracrine effects of the secretome of MSCs primed by microtopography enhanced osteoblast activity, this approach may be considered in cell therapy to regenerate bone tissue.

Financial support: FAPESP (#2022/03820-8) and CNPq (#307698/2021-1)

### P20

### Preclinical evaluation of hits from high throughput screening in osteosarcoma mouse models

Luke Tattersall, Victoria Tippett, Adrian Higginbottom, Alison Gartland

The University of Sheffield, Sheffield, UK

### Abstract

Osteosarcoma (OS) is the most common type of primary bone cancer affecting children and young adults. Treatments include standard high dose polychemotherapy with either limb salvage or amputation. Patients are often diagnosed late already presenting with metastasis and have a poor 5-year survival rate of around 30%. These survival statistics have remained constant for around 50 years with no advances in treatment options. We used high throughput screening (HTS) to identify potential candidates effective against OS. After multiple screening rounds to narrow down hits, we performed *in vitro* studies determining the $IC_{50}$, effect on cell proliferation and migration across multiple OS cell lines and carried these forward into *in vivo* models.

For *In vivo* analysis of two hits, 6-7-week old female BALB/C nude mice (n=8/group) were injected paratibially with 250,000 143B-GFP+LUC cells and paired based on IVIS signal. They then began treatment receiving 10mg/kg compound or vehicle by IP injection daily for 2 weeks. All mice developed palpable tumours which reduced in size when treated, both in IVIS signal and by calliper measurements. Analysis of the total bone volume by micro-CT analysis demonstrated only one hit reduced the presence of OS ectopic bone (P=0.0002 and P=0.3400 respectively). Finally, mouse lungs were collected at the study end point fixed, embedded, serial sectioned, H&E stained and visualised where the presence of metastasis was determined. The results showed one treatment group had reduced metastasis by 25% compared with control mice, the second compound had no effect.

In conclusion we used HTS to identify hits in OS which we validated *in vitro* and advanced into *in vivo* OS models. We examined the primary tumour, the bone and the lungs demonstrating promising results. Our ongoing work is to now test these on patient derived models of OS to ensure further translatability for patient benefit.

### P21

### Repurposing neuropeptide Y modulators to decrease viability of Ewing's sarcoma cells

Robin Rumney[1], Nancy Alnassar[1,2], Darek Gorecki[1]

[1]University of Portsmouth, Portsmouth, UK [2]Barts Cancer Institute, London, UK

### Abstract

**Introduction:** Ewing's sarcoma (EwS) is a mainly paediatric tumour with five-year survival rates of ~70% for patients with localised disease, and ~20% if metastatic. Previous reports suggest that EwS cells are affected by neuropeptide Y (NPY) acting upon receptors (NPYR), influenced by dipeptidyl-peptidase 4 (DPP4) cleavage of NPY. Availability of NPY and DPP4 may be influenced by hypoxia. We postulated that EwS could be treated by repurposing BMS-19388, an NPY1R antagonist developed for treatment of eating disorders and linagliptin, a DPP4 inhibitor developed to treat diabetes.

**Methods:** A673 and SK-ES-1 EwS cells were maintained under normoxia (atmospheric $O_2$) or hypoxia (1% $O_2$). Treatments included rhNPY (1ng/mL), rhDPP4 (20U/mL), BMS-193885 (IC50 5.9nM), and linagliptin (IC50 1nM). Expressions of *DPP4*, *NPY* and *NPYR1* were assessed by qPCR. DPP4 and NPY levels were measured by ELISA. DPP4 activity was quantified by enzymatic assay. Conditioned media (CM) was harvested and extracellular vesicles (EVs) isolated with the exoEasy Maxi Kit (Qiagen). Cell metabolism was measured with Presto blue. Viability was evaluated by live/dead labelling and crystal violet staining. Cell migration was measured using a Boyden chamber assay.

**Results:** The viability of both A673 and SK-ES-1 cells was decreased in hypoxia. Analysis of CM and EVs showed that in SK-ES-1 cells hypoxia decreased NPY protein secretion. In A673 cells hypoxia increased *DPP4* expression and the amount of DPP4 protein in secreted EVs. Interestingly, in SK-ES-1 cells, hypoxia decreased DPP4 activity in CM.

*NPY1R* was consistently expressed in normoxic and hypoxic conditions in both A673 and SK-ES-1 cultures. rhNPY increased cell viability in A673 cells in both normoxia and hypoxia. However, its effects of migration differ: NPY increased migration of A673 cells in normoxia and SK-ES-1 cells in hypoxia. BMS-193885 decreased viability of both A673 and SK-ES-1 cells.

However, rhDPP4, had differential effects, increasing viability of A673 cells in normoxia and SK-ES-1 cells in hypoxia. Treatment with linagliptin increased SK-ES-1 cell death in hypoxia, and decreased A673 viability in both normoxia and hypoxia.

**Discussion:** We investigated extracellular NPY signalling for the suitability of repurposing drugs affecting this axis for the treatment of EwS. We found that EwS cells are a source of NPY and DPP4, which can both be released via EVs. Moreover, hypoxia differentially affects this signalling axis. Blockade of the NPY1 receptor with BMS-193885 and inhibition of DPP4 with linagliptin may both represent viable therapeutic approaches. However, their effects can be tumour cell-specific and hypoxia-dependent.

### P22

### An in vitro model of secondary dystroglycanopathy reveals atypical cell behaviour during chondrogenesis

Mark Hopkinson, Leah Wells, Scott Roberts, Andrew Pitsillides

Royal Veterinary College, London, UK

### Abstract

The dystrophin-associated glycoprotein complex (DGC), relies upon a-dystroglycan-mediated actin cytoskeletal linkage to laminin globular domain (LGD)-containing ECM proteins. Its heavily glycosylated mucin-like region, is one mediator of the cell-ECM interactions that are critical in development and homeostasis. Defective a-dystroglycan glycosylation interrupts DGC function and characterises the secondary dystroglycanopathy subset of muscular dystrophies exhibiting profound muscle pathology. Poor bone health is common in these patients, where it is historically attributed to reduced muscle-induced bone loading. Our studies in mouse secondary dystroglycanopathy models have unveiled a low bone mass and modified architecture that is evident prior to any muscle damage. With data showing that LGD proteins also serve crucial skeletal functions, we therefore hypothesise that DGC-mediated cell-ECM interactions serve direct roles in controlling cell behaviour during endochondral ossification.

Accordingly, we examined DGC roles in ATDC5 chondroprogenitor cells, using the highly-selective DGC-blocking antibody, IIH6, in micromass cultures, to effectively mimic defective a-dystroglycan glycosylation. ATDC5 maintained for 0-7/7-14 days were used to replicate chondrogenic/hypertrophic phases of endochondral ossification, respectively.

Alcian blue staining revealed that IIH6 significantly restricts ATDC5 micromass size (Percentage difference 38%, $p<0.05$) during chondrogenic and hypertrophic phases, without affecting proteoglycan deposition. Alizarin red staining showed that IIH6 significantly suppressed later ATDC5 mineralisation (Percentage difference 111%. $p<0.001$), which aligned with reduced ALP activity at all stages ($p<0.05$/$p<0.01$ at days 7/14, respectively). ATDC5 migration was also diminished by IIH6 in micromass scratch assays, with lower expansion ($p<0.05$) and 'wound area' change ($p<0.0001$). DNA quantification (cell number proxy) found that IIH6 reduced ATDC5 number (7 days, $p<0.005$) and qRT-PCR for Sox9, aggrecan, collagen X and integrin-binding sialoprotein (IBSP) suggest that IIH6 prolongs ATDC5 chondrogenesis, with significantly higher Sox9 and aggrecan expression at day 14 (vs. untreated, $p<0.05$). IIH6-mediated DGC blockade, likewise, reduced levels of mineralisation by delaying hypertrophic progression, as is evidenced by significantly lower IBSP mRNA levels on day 7 ($p<0.005$) yet, intriguingly, switched this mRNA expression profile to significantly raised IBSP ($p<0.0005$) and collagen X ($p<0.0001$) mRNA levels by day 14 in the IIH6-treated ATDC5 cells.

We find that blocking DGC-ECM interactions in a chondrogenic cell line markedly limits chondrogenic and hypertrophic/mineralising behaviours in a way that may account for the low bone mass phenotypes in patients and mouse models of secondary dystroglycanopathy which we have previously described. These data indicate that DGC-mediated cell-ECM interactions may indeed serve direct roles in controlling skeletal cell behaviour during endochondral ossification.

### P23

### The effect of denosumab on osteoclast precursors in postmenopausal women

Fatma Gossiel, Marian Schini, Tanya Saini, Peter Banda, Zoe Thorton, Richard Eastell, Adreas Fontalis

University of Sheffield, Sheffield, UK

### Abstract

**BACKGROUND:** Upon denosumab discontinuation, an observed 'rebound' phenomenon in bone turnover may occur, potentially leading to a reduction in bone mineral density and the occurrence of vertebral fractures. Several theories have been proposed to explain this phenomenon, one of which is that osteoclast precursors might be accumulating during treatment. Our aim was to study the effects of denosumab on osteoclast precursors in postmenopausal women.

**METHODS:** 15 patients receiving denosumab and 15 age-matched (by decade) controls were recruited. The control patients were postmenopausal women >65years with osteoporosis or osteopenia. Exclusion criteria encompassed fracture (within the year), bisphosphonates, steroids, and bone diseases such as Paget's. 69 historic controls were used from the TRIO study with similar inclusion criteria. Blood samples were collected from each participant. Peripheral blood mononuclear cells (PBMCs) were isolated from whole blood and were stained for CD14, MCSFR, CD11b and TNFRII. Osteoclast precursors (CD14+/MCSFR+, CD14+/CD11b+ OR CD14+/TNFRII+) were identified with fluorescent activated cell sorting (FACS). The proportion of osteoclasts were determined by calculating their percentage of the total cell population in each whole blood sample. Statistical analysis was conducted using SPSS software.

**RESULTS:** Patients receiving denosumab, had a significantly higher count of CD14/CD11b osteoclast precursors compared to the TRIO controls and concurrent controls ($p<0.05$), table.

**CONCLUSION:** Our findings indicate an increase in osteoclast precursors, which could potentially explain the rebound phenomenon observed after the discontinuation of denosumab.

Table: % of osteoclast precursors in each group, Mann Whitney statistical test.

| | Treatments (N=15) | | Concurrent Controls (N=15) | | Historic controls (TRIO, N=69) | | P value* | |
|---|---|---|---|---|---|---|---|---|
| | Median | Interquartile range | Median | Interquartile range | Median | Interquartile range | Treatments vs Controls | Treatments vs TRIO Controls |
| %CD14+/ MCSFR+ | 1.5 | 3.1 - 0.7 | 0.9 | 0.4 - 2 | 1.7 | 2.6 - 1 | 0.567 | 0.661 |
| %CD14+/ CD11b+ | 4.0 | 19.1 - 2.2 | 0.75 | 0.28 – 2.29 | 2.76 | 4.2 – 1.7 | 0.011 | 0.004 |

| | | | | | | | | |
|---|---|---|---|---|---|---|---|---|
| %CD14+/ TNFRII+ | 1.3 | 3.3 - 0.3 | 1.1 | 0.3 – 2.1 | 2.39 | 3.7 – 1.4 | 0.595 | 0.136 |

### P24

### Dual inhibitory role of miR-24-2-5p in early stages of breast cancer bone metastasis formation

Margherita Puppo[1,2,3], Martine Croset[1], Davide Ceresa[4], Manoj Kumar Valluru[3], Victor Gabriel Canuas Landero[3], Michele Iuliani[5], Francesco Pantano[5], Penelope Dawn Ottewell[3], Philippe Clezardin[1,2,3]

[1]INSERM, Research Unit UMR_S1033, LyOS, Faculty of Medicine Lyon-Est, Lyon, France [2]Université Claude Bernard Lyon 1, Villeurbanne, France [3]Division of Clinical Medicine, University of Sheffield, Sheffield, UK [4]IRCCS Ospedale Policlinico San Martino, Genova, Italy [5]Medical Oncology, Fondazione Policlinico, Roma, Italy

### Abstract

### Background

Bone is the most frequent site of metastasis for breast cancer (BC). In bone, disseminated BC cells create a cancer niche by interacting with bone cells, including osteoclasts and osteoblasts, that allow cancer cell seeding and sustain their proliferation from micro- to macro- bone metastasis. MicroRNAs (miRNAs) are small RNAs that regulate the gene expression in cells and, by being released, they can function as intercellular mediators. MiRNAs are involved in the dissemination of BC in bone, and BC cell-derived miRNAs can affect the differentiation and activity of bone cells. We have previous identified that low levels of miR-24-2-5p in serum of early-stage BC patients at baseline (time of surgery) are associated with a high risk of bone metastasis. However, miR-24-2-5p role in early stages of BC bone metastasis remains ill-defined.

### Methods

MiR-24-2-5p overexpression in human BC cell line (NW1, a *luc2*-positive subpopulation of MDA-MB-231) was obtained by MIMIC-miRNA transfection. Cell proliferation, migration and invasion assays were performed to assess BC cell functions after miR-24-2-5p overexpression. An animal model was used to assess the effect of miR-24-2-5p overexpression on BC metastasis progression, as judged by bioluminescence imaging, and on bone remodelling, following serum measurement of bone resorption (CTX-I) and bone formation (P1NP) markers. The effect of conditioned medium from miR-24-2-5p-overexpressing BC cells on human osteoclast differentiation was investigated. RNA-sequencing and quantitative PCR analyses of NW1 cells were performed to evaluate transcriptomic changes associated with miR-24-2-5p overexpression.

### Results

MiR-24-2-5p overexpression in NW1 cells reduced their malignant traits (migration, invasion, proliferation) compared to controls *in vitro*. When injected intracardiacally in mice, miR-24-2-5p-overexpressing NW1 cells reduced the metastatic dissemination of BC cells, particularly in bone, compared to controls (**Fig. A**). Accordingly, the experimental group injected with miR-24-2-5p-overexpressing NW1 cells presented circulating levels of CTX-I and P1NP marker similar to naïve mice, suggesting a reduced presence of disseminated cells compared to controls. RNA-sequencing analysis and quantitative PCR on miR-24-2-5p-overexpressing NW1 cells *versus* controls revealed a number of downregulated mRNA-targets involved in tumour cell invasion or osteoclastogenesis. Interestingly, the conditioned medium from BC cells overexpressing miR-24-2-5p decreased the differentiation of human bone marrow-derived monocytes into osteoclasts *in vitro*, suggesting a dual function of miR-24-2-5p on both BC and bone cells.

### Conclusion

MiR-24-2-5p has a protective role in the first events of BC bone metastasis formation, reducing malignant BC cell traits, tumour cell dissemination in bone, and the differentiation of monocytes into mature osteoclasts.

### Figure

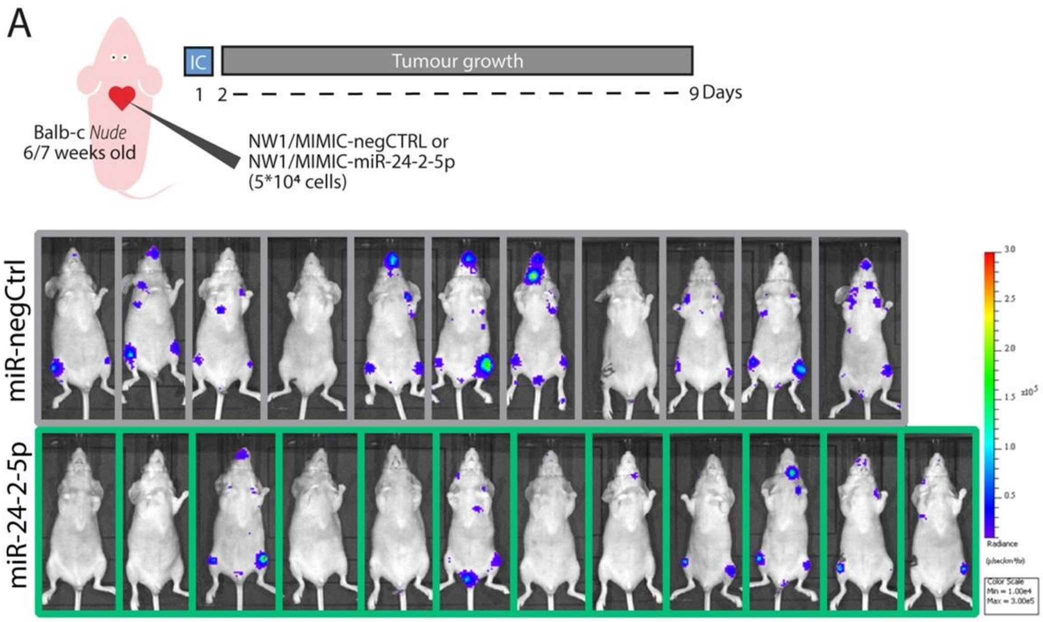

**P25**

### PHOSPHO1-mNeonGreen reporter cells are a robust model to study matrix vesicle biogenesis during live osteoblast-driven mineralisation

Charlotte Clews[1], Scott Dillon[2], Fabio Nudelman[3], Colin Farquharson[1], Louise Stephen[1]

[1]The Roslin Institute, Edinburgh, UK [2]The University of Cambridge, Cambridge, UK [3]The University of Edinburgh, Edinburgh, UK

### Abstract

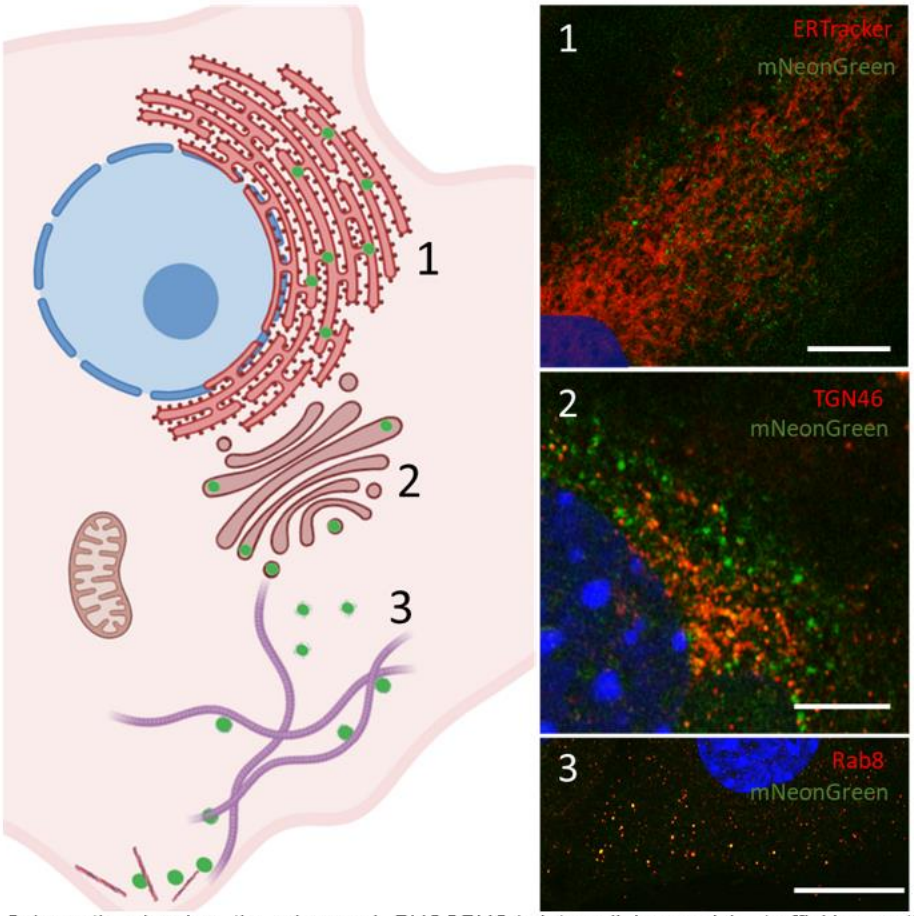

*Schematic showing the observed PHOSPHO1 intracellular vesicle trafficking pathway alongside relevant confocal imaging, including initial ER localisation (1), trans-Golgi network packaging into vesicles for secretion (2), and RAB8 mediated vesicle transport to the cell membrane (3).*

Biomineralisation is an essential process in which osteoblasts initiate the laying down of hydroxyapatite within the extracellular matrix (ECM) to form an organic-inorganic composite material that is both tough and stiff to support the physiological demands of bone. The delicate balance of inorganic phosphate and pyrophosphate concentrations is a major determinant of the rate of ECM mineralisation, and is under the control of tissue non-specific alkaline phosphatase (TNAP) and PHOSPHO1, key phosphatases involved in matrix vesicle (MV) driven mineralisation. The biogenesis of MVs is however unclear and their formation and mineral deposition within the ECM is the focus of this study.

Mineralisation time courses were performed for the osteoblast cell line MC3T3, with Alizarin red confirming mineral deposition, and qPCR and western blotting performed to ascertain gene and protein expression during the time course. Fluorescent plasmid constructs containing PHOSPHO1 fused to small fluorescent protein mNeonGreen were transiently transfected, and then stably integrated, into both fast and slow mineralising clones of MC3T3 via the PiggyBac transposase system. Live imaging of the respective intracellular localisation of PHOSPHO1-mNeonGreen was performed in set timepoints during mineralisation, and co-immunofluorescence was used to establish PHOSPHO1 localisation throughout the cell.

PHOSPHO1-mNeonGreen expressing cells are a robust model for imaging the intracellular stages of MV production and biogenesis by osteoblasts. Using fluorescent PHOSPHO1 as a marker, we have followed the trafficking pathway of MV precursors in the endoplasmic reticulum to the trans-Golgi network; a process involving RAB8 vesicles and driven by microtubules. This suggests a RAB GTPase-dependant method of intracellular vesicle packaging and subsequent MV secretion by the osteoblast. Furthermore, confocal imaging shows PHOSPHO1 positive MVs appear to be released at actin-rich sites at the osteoblast cell membrane, possibly linked to focal adhesion sites. Timelapse confocal imaging of cells surrounding mineralised nodules produced *in vitro* shows small PHOSPHO1-mNeonGreen objects present and interacting at the mineral-cell membrane boundary, suggesting evidence of MV activity. Preliminary evidence with a TNAP-mKate2+PHOSPHO1-mNeonGreen double reporter line shows co-localisation of both MV markers on these intracellular objects, with object size and localisation as supporting evidence for an MVB driven method of MV release.

### P26

### A study to investigate the influence of microporous scaffold functionalisation with hydroxyapatite on bone cell mineralisation for effective bone regeneration

Meghna Ray[1,2], Meghna Suvarna[2], Nicola Green[2]

[1]Sheffield Teaching Hospitals, Sheffield, UK [2]University of Sheffield, Sheffield, UK

### Abstract

Hydroxyapatite (HA) is the main mineral component of bone. It functions by providing bone strength and aids in bone regeneration. HA has been historically incorporated into scaffolds used for regeneration of bone tissue to encourage mineralisation. This study aims to determine whether HA integration into poly (glycerol sebacate) methacrylate (PGSM) based polyHIPE scaffolds improves bone mineralisation by optimisation of pore size and porosity to encourage cell infiltration and differentiation.

High internal phase emulsion (HIPE) templating techniques were used in production of microporous scaffolds for bone regeneration. PGSM at 80% methacrylation (PGSM-80) was the material of choice. PGSM-80 was selected due to the suitable materials properties that it imparts once cured and its known biocompatibility and biodegradability.

Two scaffolds have been compared in this study; PGSM-80-HA and PGSM-80 made at 50℃. PGSM-80-HA scaffolds were produced with HA solution as the aqueous phase of emulsion templating, in place of deionised water used in PGSM-80 scaffolds. Cured scaffolds of both polymers have a calculated theoretical porosity of 74%, allowing for a direct comparison of the effects of HA upon the osseous tissue regenerated. Theoretical porosity has been calculated following helium pycnometry.

Visualisation of polyHIPE scaffolds via scanning electron microscopy (SEM) allowed for determination of pore size and structure. Materials properties of the scaffolds were evaluated via compression testing, and calculation of stiffness from the linear elastic region of the stress-strain curve.

Murine osteoblasts (MLO-A5) were seeded upon sterilised scaffolds and cultured to assess cell viability, mineralisation, and infiltration.

A resazurin assay was conducted on both scaffolds and compared to a positive control of tissue culture plastic to assess cell viability. Results from this assay are demonstrated in the following figure.

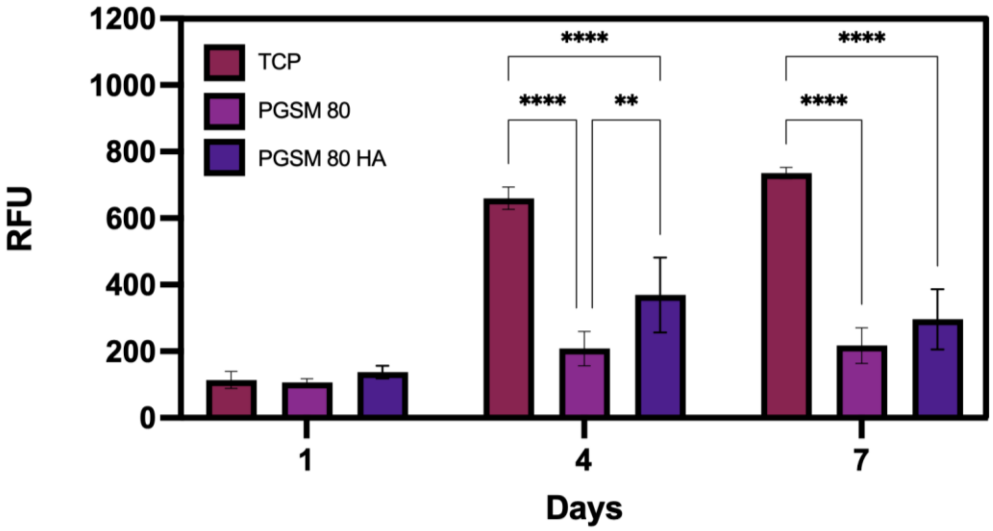

Alazarin Red is the assay of choice for mineralisation, giving qualitative analysis of the extent of mineralisation with and without the influence of HA by comparison of both scaffolds.

Hematoxylin and eosin staining allowed for assessment of the extent of cell infiltration of the scaffolds. This data gives information regarding suitability of pore size and interconnectivity throughout the scaffold structure.

In summary, this work aims to assess the influence of HA upon scaffold structure and suitability for regeneration of osseous tissue by direct comparison of PGSM-80 and PGSM-80-HA. A range of methods have been used to examine the material properties of both materials and their biocompatibility and appropriateness for effective regeneration of bone tissue.

### P27

### Potential role of MicroRNA-155 in type 2 diabetic osteopathy

Mouza Mohammed Alaleeli, Suneesh Kaimala, Sahar Mohsin

Department of Anatomy, College of Medicine and Health Sciences, United Arab Emirates University, Al Ain, UAE

### Abstract

Type 2 diabetes mellitus (T2DM) is known to increase the risk of fragility fractures, however; the underlying mechanism is still elusive. Novel microRNAs, such as microRNA-155 have been suggested to play vital roles in the regulation of bone remodeling. Deficiency in miR-155 expression and increased expression of RhoA is known to promote bone resorption. The aim of the study was to investigate any imbalance in the remodeling cycle in type 2 diabetes by measuring the changes in the expression profile of miR-155 and RhoA in T2DM. The study was carried out on the bones stored at -80 degrees from an earlier study. Briefly, three-month-old female Wistar rats were fed with a high-calorie diet for 3 weeks followed by intraperitoneal injection of two lower doses of streptozotocin at weekly intervals to induce T2DM. Analysis of tibial bones from Wistar rats that were diabetic for 10 weeks were tested for the expression of miR-155 and RhoA using qRT-PCR which showed reduced levels of miR-155 and raised levels of RhoA. CTx-1 level was increased by 20.84% and both OPG and RANKL levels were decreased in the diabetic rats. The OPG/RANKL ratio was low in diabetic rats, which favors bone resorption. Proinflammatory cytokines IL-1β and TNF-α were increased in the diabetic rats. Despite the low expression of RANKL, bone resorption is more likely to occur in diabetic rats as both IL-1β and TNF-α work synergistically together to promote osteoclastogenesis. MiR-155 could be an important modulator of bone remodeling in T2DM and a potential diagnostic biomarker or therapeutic target for diabetic osteopathy.

### P28

### Priming periosteal cells for skeletal regeneration following a One Health approach

Neil Marr, Leah Wells, Isabelle Matthews, Deborah Guest, Richard Meeson, Scott Roberts

Royal Veterinary College, London, UK

### Abstract

The periosteum is skeletal stem/progenitor cell niche that is critical to skeletal homeostasis. Periosteum-derived cells (PDCs) have enhanced osteochondrogenic potential that can be harnessed for skeletal regeneration. Given the conserved aetiological factors across species with predisposition to bone/cartilage degeneration, our work seeks to exploit PDCs for cross-species skeletal repair through in vitro phenotype modification and tissue engineering. Here, we aim to (a) establish novel procedures for in vitro drug screening of both horse and human PDCs, and (b) to identify PDC-priming compounds which could potentiate the regeneration of the osteochondral tissues.

First, optimal in vitro expansion and osteogenic differentiation conditions were established for equine PDCs (ePDCs; n=2, age=5 &14 years) using both human biomimetic (growth factor cocktail) and standard in-house osteogenic cell culture conditions. Osteogenic potential was assessed by alizarin red staining at day 7 and day 21. Initial testing of chondrogenic differentiation using ePDC monolayer cultures was also assessed at day 7 by alcian blue staining. To identify novel candidates for PDC-mediated skeletal regeneration, undifferentiated ePDCs and human PDCs (hPDCs; n=6 donors pooled) were treated with a small molecules containing predicted PDC-activating compounds (selected using the L1000 Connectivity Map) for 24 h to screen for acute effects on osteochondral potential. Cell viability and proliferation was assessed using a resazurin assay following treatment, and gene expression (qPCR) of PDC and osteochondrogenic markers were analysed to screen for pro-regenerative phenotypes. We also compared effects alongside species-specific MSC cells lines.

At day 7, osteogenic potential of ePDCs was enhanced through supplementation with horse serum when compared to cultures treated with foetal bovine serum (1.2- fold). This is analogous to our previous studies showing human PDCs have enhanced in vitro mineralisation capacity when cultured in xeno-matched serum compared to FBS (1.5 fold; $p<0.01$). Additionally, preliminary chondrogenic testing of ePDC suggests horse serum enhanced proteoglycan deposition. In undifferentiated PDCs, acute treatment with L1000 small molecules identified compounds capable of modifying periosteal cell behaviour with particular reference to early markers of stem cell activation.

Our work thus far demonstrates the highly transferrable conditions and translational potential for periosteal cell biology across species. This current body of work will act as a foundation for future research exploring small molecule treatment aimed at enhancing skeletal tissue formation, both in vitro and in vivo. In the long term, our research aims to establish molecular targets for PDC-mediated skeletal regeneration in both clinical and veterinary practice.

**P29**

*Withdrawn*

**P30**

*Withdrawn*

**P31**

*Withdrawn*

**P32**

*Withdrawn*

### P33

### Employment type and risk of osteoarthritis: A cross-sectional study of 285,947 UK biobank participants

Asad Hashmi[1], Sophie Scott[1], Mijin Jung[1], Qing-Jun Meng[2], Jon Tobias[1], Rhona Beynon[1], Ben Faber[1]

[1]University of Bristol, Bristol, UK [2]University of Manchester, Manchester, UK

### Abstract

### Background

Osteoarthritis (OA) is a globally leading cause of disability, imposing a considerable socioeconomic burden. Identifying occupational risk factors could inform interventions to reduce this burden. Shift work, known to disrupt circadian rhythms, has emerged a risk factor in other diseases. Chondrocyte activity exhibits diurnal variation, and genetic deletion of clock genes has been associated with cartilage degeneration, suggesting a possible connection between shift work and OA. Additionally, heavy manual work is a recognised risk factor for OA and might confound the associations between shift work and OA. We aimed to investigate the independent associations of shift work and physical work with large joint OA.

### Methods

Self-reported employment data were collected through questionnaires from UK Biobank (UKB) participants. Participants reported their engagement with shift work, night shifts, heavy manual work or non-sedentary work. Responses were categorised into never/sometimes or usually/always binary variables. Knee OA (KOA) and hip OA (HOA) diagnoses were extracted from hospital episode statistics. Logistic regression models examined associations between employment factors and OA outcomes. Adjustments included age, sex, BMI, Townsend Deprivation Index (TDI) and other employment factors.

### Results

This study comprised 285,947 participants (mean age: 52.7, SD: 7.1, range: 38-71). KOA and HOA were diagnosed in 18,578 (6.5%) and 10,698 (3.7%) participants, respectively. Additionally, 16,407 (5.7%) reported non-site-specific OA. There was strong evidence of associations between both shift work and night shift work, with KOA (fully adjusted model: OR 1.12 [95% CI 1.07-1.17] and 1.12 [1.04-1.20] respectively). Similar associations were seen for self-reported OA. After adjustment, there was little evidence for associations between shift work and night shifts with HOA (1.01 [0.95-1.08] & 1.03 [0.93-1.14] respectively). Heavy manual work and non-sedentary work were associated with increased risk of all OA outcomes (Table 1).

### Conclusions

Independent associations were seen between shift work and both KOA and self-reported OA but not HOA. Previously, in mouse experiments loss of cartilage-specific circadian regulatory elements was associated with KOA, but not HOA. Taken together these findings suggest a role for circadian rhythm dysfunction in KOA but not HOA pathogenesis, warranting further investigation. Heavy manual work and non-sedentary work were associated with all OA outcomes, with stronger associations observed in KOA, compared to HOA, possibly reflecting the knee's higher susceptibility to biomechanical stress. Further investigation is needed to understand if workplace interventions can decrease these risks.

**Table 1.** Logistic regression results of employment exposures and osteoarthritis outcomes

| | Unadjusted | | Adjusted age & sex | | Adjusted age, sex, BMI & TDI | | Adjusted age, sex, BMI, TDI & other work variables | |
|---|---|---|---|---|---|---|---|---|
| **Knee OA** | OR [95% CI] | P | OR [95% CI] | P | OR [95% CI] | P | OR [95% CI] | P |
| Shift work | 1.31 [1.25-1.37] | $8.87 \times 10^{-32}$ | 1.42 [1.36-1.49] | $6.30 \times 10^{-52}$ | 1.29 [1.23-1.35] | $3.73 \times 10^{-27}$ | 1.12 [1.07-1.17] | $3.87 \times 10^{-6}$ |
| Night shifts | 1.32 [1.23-1.42] | $1.26 \times 10^{-15}$ | 1.46 [1.36-1.57] | $6.61 \times 10^{-27}$ | 1.30 [1.21-1.39] | $3.39 \times 10^{-13}$ | 1.12 [1.04-1.20] | $1.75 \times 10^{-3}$ |
| Heavy manual work | 1.55 [1.49-1.61] | $2.00 \times 10^{-113}$ | 1.59 [1.53-1.65] | $3.00 \times 10^{-122}$ | 1.58 [1.52-1.64] | $4.00 \times 10^{-114}$ | 1.30 [1.24-1.36] | $5.76 \times 10^{-30}$ |
| Non-sedentary work | 1.48 [1.44-1.53] | $2.00 \times 10^{-146}$ | 1.46 [1.41-1.50] | $7.00 \times 10^{-131}$ | 1.48 [1.43-1.52] | $3.00 \times 10^{-135}$ | 1.32 [1.28-1.37] | $5.30 \times 10^{-54}$ |
| **Hip OA** | OR [95% CI] | P | OR [95% CI] | P | OR [95% CI] | P | OR [95% CI] | P |
| Shift work | 0.99 [0.93-1.05] | 0.74 | 1.12 [1.05-1.20] | $5.60 \times 10^{-4}$ | 1.08 [1.01-1.15] | 0.03 | 1.01 [0.95-1.08] | 0.74 |
| Night shifts | 0.97 [0.88-1.07] | 0.54 | 1.16 [1.04-1.28] | $5.24 \times 10^{-3}$ | 1.10 [0.99-1.21] | 0.08 | 1.03 [0.93-1.14] | 0.59 |
| Heavy manual Work | 1.13 [1.07-1.19] | $1.00 \times 10^{-5}$ | 1.22 [1.15-1.29] | $1.55 \times 10^{-12}$ | 1.22 [1.15-1.28] | $2.87 \times 10^{-12}$ | 1.13 [1.06-1.20] | $1.32 \times 10^{-4}$ |
| Non-sedentary Work | 1.19 [1.14-1.24] | $5.10 \times 10^{-18}$ | 1.16 [1.11-1.21] | $3.53 \times 10^{-13}$ | 1.17 [1.12-1.21] | $5.17 \times 10^{-14}$ | 1.12 [1.07-1.17] | $1.39 \times 10^{-6}$ |
| **Self-reported OA** | OR [95% CI] | P | OR [95% CI] | P | OR [95% CI] | P | OR [95% CI] | P |
| Shift work | 1.15 [1.09-1.21] | $6.83 \times 10^{-8}$ | 1.32 [1.25-1.39] | $1.16 \times 10^{-26}$ | 1.23 [1.17-1.30] | $1.51 \times 10^{-15}$ | 1.13 [1.07-1.19] | $5.54 \times 10^{-6}$ |
| Night shifts | 1.11 [1.03-1.20] | $6.20 \times 10^{-3}$ | 1.37 [1.27-1.48] | $3.56 \times 10^{-15}$ | 1.26 [1.16-1.36] | $1.22 \times 10^{-8}$ | 1.15 [1.06-1.24] | $7.00 \times 10^{-4}$ |
| Heavy manual work | 1.21 [1.16-1.26] | $1.21 \times 10^{-17}$ | 1.36 [1.30-1.42] | $7.30 \times 10^{-43}$ | 1.34 [1.28-1.40] | $2.95 \times 10^{-38}$ | 1.20 [1.14-1.27] | $5.54 \times 10^{-13}$ |
| Non-sedentary work | 1.26 [1.22-1.30] | $5.30 \times 10^{-46}$ | 1.24 [1.20-1.28] | $4.96 \times 10^{-38}$ | 1.24 [1.20-1.28] | $2.87 \times 10^{-37}$ | 1.15 [1.10-1.19] | $1.27 \times 10^{-12}$ |

### P34

### DXA-derived hip shape predicts hip fractures: A longitudinal study from 38,123 participants in UK biobank

Sophie Scott[1], Asad Hashmi[1], Raja Ebsim[2], Fiona R Saunders[3], Mijin Jung[4], Jennifer S Gregory[3], Richard M Aspden[3], Claudia Lindner[2], Timothy Cootes[2], Nicholas C Harvey[5], Jonathan H Tobias[4], Benjamin G Faber[4], Rhona Beynon[4]

[1]University of Bristol, Bristol, UK [2]Division of Informatics, Imaging and Data Science, University of Manchester, Manchester, UK [3]Centre for Arthritis and Musculoskeletal Health, University of Aberdeen, Aberdeen, UK [4]Musculoskeletal Research Unit, University of Bristol, Bristol, UK [5]Medical Research Council Lifecourse Epidemiology Unit, University of Southampton, Southampton, UK

### Abstract

**Objective**: Hip fractures are a severe consequence of bone fragility, with a 20% one-year mortality rate. Measures of hip geometry have previously been associated with hip fracture but are highly correlated thus pose challenges in determining which measurements are needed for optimal fracture prediction. Statistical shape modelling (SSM) is a method developed to derive hip shape more holistically than individual geometric parameters (GPs). The aim of this study was to investigate whether global hip shape derived using SSM applied to dual-energy X-ray absorptiometry (DXA) can predict hip fracture, independently of corresponding GPs.

**Methods**: SSM was applied to automatically placed outline points on left hip DXA scans obtained from UK Biobank and produced ten orthogonal hip shape modes (HSM) that captured 86% of shape variance. Femoral neck width (FNW) was also derived from the outline points using a custom Python script. Cox proportional hazard models were used to examine the longitudinal associations between each HSM and hospital diagnosed hip fractures. Adjustments were made for model 1: age, sex, height, weight, total hip bone mineral density (BMD), and model 2: model 1 plus FNW. A correlation coefficient was used between HSM2 and FNW. A Bonferroni adjusted p-value threshold ($p<0.005$) was used to account for the ten HSMs tested.

**Results:** Our study included 38,123 participants (mean age 63.7 years; 52% female; mean follow-up time 5 years). 133 individuals (0.35%) had a hospital diagnosed hip fracture. HSM2 is characterised by a narrower femoral neck, higher neck shaft angle (NSA) and greater acetabular coverage (Figure 1). Although, there is no correlation between HSM2 and FNW ($r^2 = 0.0004$). HSM2 showed strong evidence of an association to hip fracture (model 1 - HR: 1.31, 95% confidence interval (CI): 1.11-1.55, p-value 0.002). This association held despite further adjustment for FNW (model 2 – HR: 1.33, 95% CI: 1.12-1.57, p-value 0.001). There was no evidence for other HSMs being associated with hip fracture.

**Conclusion**: In this longitudinal study, we identified an association between DXA-derived HSM2 and hip fracture that was independent of total hip BMD and FNW. Unfortunately, NSA could not be derived due to limited views of the femoral shaft. Further work is justified to understand if hip shape measures, automatically derived from DXA scans already undertaken for BMD measurements, could be incorporated into FRAX to improve fracture prediction.

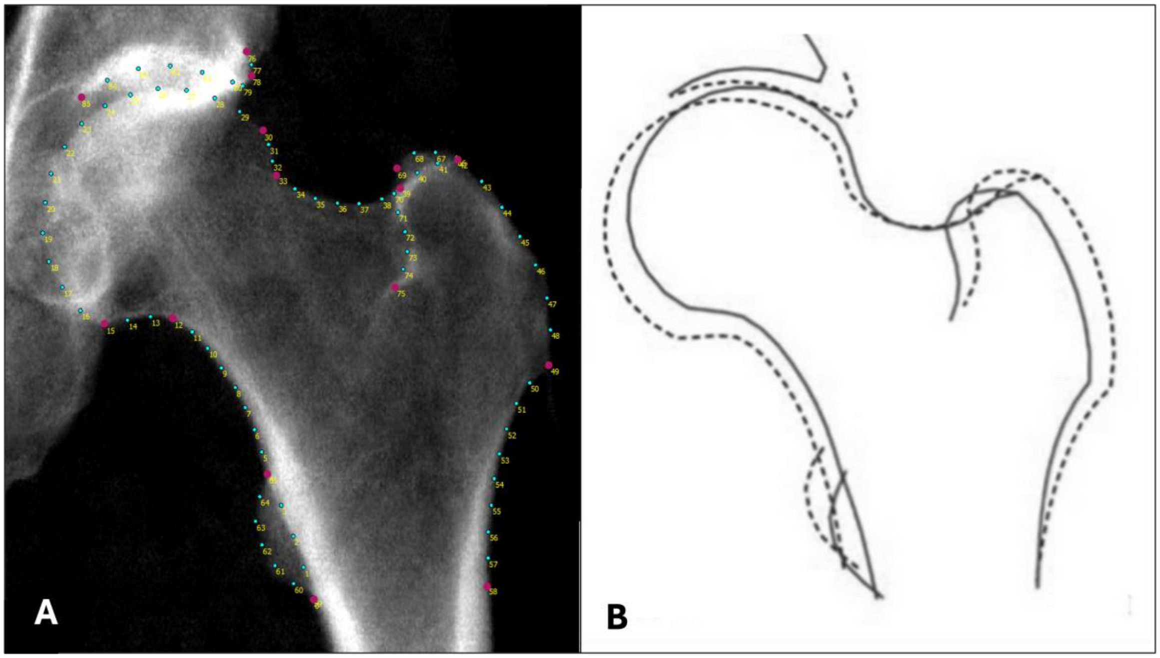

**Figure 1:** A) Example DXA image with points around the hip, B) HSM2 solid line +2 SDs, dotted line -2 SDs.

### P35

### Greater MRI thigh muscle mass is linked to lower incident fracture risk in women, but not men: A UK Biobank study

Elizabeth Curtis[1], Stefania D'Angelo[1], Rebecca Moon[1,2], Julien Paccou[3], Nicholas Fuggle[1], Elaine Dennison[1], Cyrus Cooper[1], Kate Ward[1], Nicholas Harvey[1]

[1]MRC Lifecourse Epidemiology Centre, University of Southampton, Southampton, UK [2]Department of Paediatric Endocrinology, University Hospital Southampton NHS Foundation Trust, Southampton, UK [3]Université Lille Nord-de-France, Lille, France

### Abstract

Objectives

Dual-energy x-ray absorptiometry (DXA) measures of appendicular lean mass (DXA-ALM) have been shown to have modest predictive value for incident fractures, with some studies showing that the predictive value of DXA-ALM for future fractures is attenuated (and potentially inverted) after adjustment for femoral neck BMD. We therefore investigated associations between a gold-standard measure of muscle mass, MRI thigh muscle volume (MRI-TMV), and incident fractures.

Methods

Participants in the UK Biobank Imaging Study (a subset of the UK Biobank Study) underwent MRI examination, from neck to knees. Automated analysis was performed using the AMRA Profiler™ system, to segment total thigh muscle volume, visceral adipose tissue (VAT) and abdominal subcutaneous adipose tissue (ASAT). Cox regression was used to calculate hazard ratios for incident fractures, per standard deviation greater MRI-TMV/height$^2$, in men and women separately, adjusting for age, Townsend deprivation score, ethnicity, prior fracture, alcohol consumption and smoking in all models, and then additionally for VAT, ASAT, body mass index (BMI) or heel quantitative ultrasound estimated (e)BMD. Participants were followed until first fracture, death, loss to follow-up or 30/9/2021.

Results

There were 25,117 participants (mean age 62.3 in women, 63.8 years in men) with an MRI-TMV measure, undertaken between 2014 and 2018. The outcome was first fracture of any type occurring after the MRI; there were 308 incident fractures in women (2.4%) and 200 in men (1.7%).

Greater TMV/height$^2$ was associated with a lower risk of any fracture in women even after adjustment for prior fracture [HR 0.82, 95% CI (0.70,0.97)], but with a trend towards an increased risk of fracture in men [HR 1.17 (0.94,1.46)]. Adjustment for measures of adiposity, such as VAT, ASAT or BMI did not change the observed associations in women but strengthened them in men [in men, with adjustment for BMI, HR 1.39 (1.07,1.81)]. Adjustment for BMD measured by heel ultrasound (eBMD) did not alter the findings.

Conclusions

The finding that that greater thigh muscle volume had a protective effect on fractures in women, but was associated with increased fracture risk in men, is in keeping with some DXA-ALM studies. This supports further investigation into a potential causal, rather than measurement-specific, association between greater muscle mass and higher fracture risk in men. Further work is required to understand the influence of intramuscular fat and whether such effects are seen in typical osteoporotic fracture sites. This work was undertaken using the UK Biobank resource under approved application 3593.

### P36

### Smoking history and fracture risk; a meta-analysis

Marian Schini[1], Helena Johansson[2], Nicholas Harvey[3], John Kanis[1], Eugene McCloskey[1]

[1]University of Sheffield, Sheffield, UK [2]University of Gothenburg, Gothenburg, Sweden [3]University of Southampton, Southampton, UK

### Abstract

### Objectives

Smoking is associated with an increased risk of fractures, with current smoking as a risk factor in the FRAX® tool. In this meta-analysis of international cohorts, we examined the relationship of smoking (current and past) with fracture risk.

### Materials and methods

The risk of fracture associated with smoking was estimated by an extended Poisson model applied separately to each of 57 prospective cohort studies. Covariates included time since start of follow up, current age, and in an additional model, BMD at the femoral neck. The results were merged by using the inverse-variance weighted β-coefficients.

### Results

Analysis included a total of 1 634 449 participants (60% female, mean age 60 years). Current smoking (12% of participants, 15.3% men, 9.8% in women), was associated with a significantly increased risk of any fracture in both sexes (Table). It was associated with a greater increase in fracture risk in men than in women. While the hazard ratio (HR) was attenuated with the inclusion of BMD, it remained statistically significant in all categories, suggesting the risk is largely independent from BMD. When compared to non-smokers, there was little evidence for increased fracture risk amongst women who were past smokers for most fracture categories. In men, past smoking was significantly associated with fracture risk [1.11, 95% CI 1.03, 1.18]; however, the magnitude of the effect was lower than observed for current smokers [1.43, 1.26, 1.62)].

### Conclusions

We confirm an association between current smoking and increased fracture risk that is largely independent of BMD. The attenuation of the smoking associated fracture risk in past vs current smokers supports skeletal benefits of smoking cessation.

Table: Hazard ratio (HR) and 95% confidence intervals (CI) for fractures in current smokers in men and women. Abbreviations: BMD= bone mineral density; HR= hazard ratio; CI= confidence intervals; MOF= major osteoporotic fracture; OST= osteoporotic fracture

| | Adjusted for age and time since baseline | | Adjusted for age and time since baseline – for **those with BMD** | | Adjusted for age, time since baseline **and BMD** | |
|---|---|---|---|---|---|---|
| | Cohorts (N) | HR (95% CI) | Cohorts (N) | HR (95% CI) | Cohorts (N) | HR (95% CI) |
| ***Female*** | | | | | | |
| Hip | 39 | 1.60 (1.48-1.74) | 33 | 1.60 (1.46, 1.76) | 33 | 1.48 (1.34, 1.64) |
| MOF | 46 | 1.21 (1.15-1.27) | 39 | 1.22 (1.16, 1.29) | 39 | 1.16 (1.10, 1.22) |
| ***Male*** | | | | | | |
| Hip | 24 | 1.75 (1.55, 1.97) | 19 | 1.83 (1.59, 2.10) | 18 | 1.57 (1.30, 1.89) |
| MOF | 30 | 1.37 (1.26, 1.50) | 27 | 1.37 (1.24-1.51) | 27 | 1.19 (1.08, 1.31) |

### P37

### The influence of ethnicity and deprivation on the occurrence of Paget's disease in the UK

Adrian Heald[1,2], Wenqi Lu[3], Richard Williams[4], John Warner-Levy[2], Kevin McKay[5], Asri Maharani[6], Michael Cook[7], Terence O'Neill[7]

[1]The School of Medicine and Manchester Academic Health Sciences Centre, Manchester University, Manchester, UK [2]Department of Endocrinology and Diabetes, Salford Royal Hospital, Salford, UK [3]Department of Computing & Mathematics, Faculty of Science and Engineering, Manchester Metropolitan University, Manchester, UK [4]UK NIHR Manchester Biomedical Research Centre, Manchester University, Manchester, UK [5]Faculty of Science and Engineering, Manchester Metropolitan University, Manchester, UK [6]Department of Nursing, Faculty of Health and Education, Manchester Metropolitan University, Manchester, UK [7]Centre for Epidemiology Versus Arthritis, Division of Musculoskeletal and Dermatological Sciences, Faculty of Biology Medicine and Health, University of Manchester, Manchester, UK

### Abstract

Introduction:

Significant variation exists in the occurrence of Paget's disease in different regions and populations. There is little data concerning the occurrence of clinically apparent disease in black and ethnic minority groups in the UK. We looked at the occurrence of the disease in a large urban conurbation in the UK and explored the influence of age, gender, and ethnicity on occurrence. We also looked at the impact of Paget's disease in influencing the severity of health outcomes after acute COVID-19 infection.

Methods:

We undertook an anonymised search using an integrated primary and secondary care-based database in Greater Manchester, with a population of over 3 million people. We looked at the occurrence of clinically diagnosed Paget's disease in January 2020 in men and women over 60 and assessed gender, deprivation level (using the Townsend Index and expressed in quintiles), and ethnicity (based on self-report). We also looked among those with a first positive COVID-19 test and assessed the influence of Paget's disease on subsequent admission to the hospital within 28 days.

Results:

534,571 people aged 60 years and over were alive on 1 January 2020. The majority were white (84%) with 4.7% describing themselves as Asian or Asian British, and 1.27% Black or Black British. There were 931 with clinically diagnosed Paget's disease. The overall prevalence in the greater Manchester area was 0.174%. Prevalence was higher in men than women (0.195 vs 0.155%). Compared to the prevalence of Paget's in whites (0.179%) the prevalence was lower in those of Asian or South Asian descent (0.048%) and higher in those of Black / Black British descent (0.344%). Prevalence increased with increasing social deprivation. After adjustment for age, gender, and deprivation the risk of disease remained lower in Asians (OR=0.36) and higher in black British (OR=2.13). Among those with a positive COVID-19 test, those with Paget's were more likely to require admission to the hospital within 28 days though the confidence intervals embraced unity (OR 2.43; 95% CI (0.94, 1.95).

Conclusion:

Clinically apparent Paget's disease is uncommon affecting less than 2 per thousand men and women. Within Greater Manchester, it appears to be more common in those of black or black British descent and less common in those of Asian or South Asian descent. Further research is required to determine whether these differences are due to disease occurrence or presentation variations.

### P38

### Family history of fracture in the FRAX tool: An updated meta-analysis

Eugene McCloskey[1], Helena Johansson[2], Marian Schini[1], Nicholas Harvey[3], Mattias Lorentzon[2], John Kanis[1]

[1]University of Sheffield, Sheffield, UK [2]University of Gothenburg, Gothenburg, Sweden [3]University of Southampton, Southampton, UK

### Abstract

### Objectives

To undertake a meta-analysis of international prospective cohorts to quantify the relationship between a family history of fracture and future fracture incidence, when adjusted for age, sex, time since baseline and femoral neck bone mineral density (BMD).

### Materials and Methods

We investigated the relationship between family hip fracture or any fracture history and the risk of any clinical fracture, any osteoporotic fracture, major osteoporotic fracture (MOF) and hip fracture alone using an extended Poisson model in each cohort. Models were adjusted for current age, sex, BMD, and follow up time. The results of the different studies were merged using inverse weighted β-coefficients. We evaluated if the association between family fracture history differed age, follow-up time, and parental fracture origin (maternal vs. paternal) using traditional interaction terms.

### Results

The interim analysis dataset comprised up to 281,893 men and women from up to 40 cohorts in 28 countries followed for a total of 2.42 million person-years. After adjustment for age and time since baseline, parental hip fracture was associated with higher risk of incident fracture across all fracture outcome categories in both men and women. The association was strongest for incident hip fractures alone [Hazard Ratio (95% CI): 1.40 (1.26-1.56) and 1.37 (1.05-1.80) in women and men respectively], and the increased fracture risk was similar by sex across all incident fracture categories. Associations were largely unchanged when additionally adjusted for BMD, did not vary by age or follow-up time, and were similar if the history of hip fracture was maternal or paternal. In a limited analysis of sibling history of hip fracture or any fracture, both demonstrated similar associations to those observed with parental history.

### Conclusions

In this large international cohort meta-analysis, a family history of fracture is confirmed as a significant independent predictor of future fracture risk. While parental hip fracture appears the strongest factor for future hip fracture, the findings also suggest that a family history of other fractures might be appropriate for inclusion in future iterations of the FRAX tool.

### P39

### Epidemiology of metatarsal fractures in Shropshire 2021-2024

Mark Garton[1], Jenni Rowlands[1], Richard Roach[2]

[1]Royal Shrewsbury Hospital, Shrewsbury, UK [2]Shrewsbury and Telford Hospitals NHS Trust, Shrewsbury, UK

### Abstract

Background: Metatarsal fractures are common and disabling fractures, frequently triggered by low-energy injuries, or less often by repetitive loading. 'Atypical' metatarsal fractures have also been reported during prolonged anti-resorptive therapy, but a causal relationship remains speculative. Despite their clinical and economic importance, the epidemiology of metatarsal fractures remains poorly understood. We present a detailed service evaluation of radiologically-confirmed metatarsal fractures identified within Shropshire, over a three-year period.

Methods: Radiology reports for all adults (≥18 years) attending hospital or community radiology departments between May 2020 and April 2023, were searched electronically using the terms 'metatarsal' AND 'fracture'. All distinct patient episodes with ≥1 confirmed metatarsal fracture were identified for further analysis, and all duplicates were excluded. Age at fracture, sex, fracture type/distribution and mechanism of injury (where known) was recorded. Age- and sex-specific fracture rates were estimated by decade of life, using contemporaneous demographic data.

Results: 1121 (758 female) patient-episodes of metatarsal fracture were identified, comprising 312, 397 and 412 individuals in years 1-3 respectively. Mean (SD) age at fracture was 50.7 (18.9) years, with 961 (85.7%) metatarsal fractures occurring in isolation, most (n= 736) affecting the fifth metatarsal. Smaller numbers fractured two (n=72), three (n=63), or four (n=15) metatarsal bones. Most fractures (59%) affected the metatarsal base, and less often the shaft (26%), neck (12%) and head (3%). Fractures were most often oblique (n=410), transverse (n=359) or comminuted (n=120), and only 70 patients had stress fractures. Estimated trauma levels were low (n=632), moderate (n=109), high (n=43) and unknown (n=246), and inversion and falls-related injuries the most commonly reported mechanism. Estimated annual fracture rates per 100,000, increased from 105 among women aged 18-29 years to 153 aged 50-59 years, with a second peak of 142 aged 80-89 years. For men at the same time points, rates were 102, 40 and 31. Although numerically less frequent, metatarsal neck fractures showed the greatest sex difference, becoming exponentially more common in women with age, compared to a gradual age-related decline in men.

Discussion: Metatarsal fractures are common, usually affecting the fifth metatarsal in isolation, and often caused by low energy injuries, as shown by previous authors. Lower incidence during Covid lockdown may be genuine or reflect reduced ascertainment. Our estimates of age- and sex-specific fracture rates suggest pronounced sex differences in metatarsal fracture rates with age, which requires further study and linkage to known risk factors and relevant medication exposure

### P40

### Metaphyseal trabecular bone separation is bimodal: A methodological update

Carmen Huesa[1], John Lockhart[2], Carl Goodyear[1], Jonathan Williams[3]

[1]School of Infection and Immunity, University of Glasgow, Glasgow, UK [2]Institute of Biomedical and Environmental Health Research, University of the West of Scotland, Glasgow, UK [3]Department of Biomedical Engineering, University of Strathclyde, Glasgow, UK

### Abstract

Micro-computed tomography with morphometric analysis is the gold standard methodology for skeletal phenotyping of small animal models of bone disease. Metaphyseal trabecular bone is the most common site of assessment. The 2010 guidelines for the assessment of bone microstructure recommends a minimal set of parameters to be reported; bone volume fraction (BV/TV), and trabecular number (Tb.N), thickness and separation (Tb.Sp). Utilising three different models of osteoporosis (rat spinal cord injury, mouse ovariectomy and mouse ageing), we demonstrate that metaphyseal Tb.Sp is bimodal (Figure 1A) both in the femur of tibia. We propose that metaphyseal Tb.Sp should be reported as two distinct values - preliminarily named $Tb.Sp_1$ (representing trabecular thickening/thinning) and $Tb.Sp_2$ (representing marrow cavity expansion).

BV/TV, Tb.N and Tb.Sp analyses were performed on metaphyseal trabecular bone VOIs. The outputs from Tb.Sp analysis are the volume-weighted average Tb.Sp, Tb.Sp histogram, and Tb.Sp dataset (Figure 1). Metaphyseal Tb,Sp histograms appeared bimodal (or multimodal), which is particularly clear in osteoporotic datasets (Figure 1A). A global threshold (350µm) was applied, separating the two peaks in the distribution, enabling acquisition of $Tb.Sp_1$ and $Tb.Sp_2$ VOIs. The $Tb.Sp_1$ (or $Tb.Sp_2$) VOI was then applied to the original dataset and the Tb.Sp analysis was rerun, generating results for $Tb.Sp_1$ (or $Tb.Sp_2$) (Figure 1C).

For the age-induced osteoporosis datasets (14-, 36- and 62-weeks-old), a monotonic decrease in BV/TV and Tb.N, and increase in Tb.Sp was observed ($p < 0.01$) confirming age-induced osteoporosis. Tb.Sp histograms were multimodal (Figure 1A). Segmenting the Tb.Sp dataset based on sphere diameter enabled the distinction of effects due to intra-trabecular thinning (first peak) captured by $Tb.Sp_1$, from those due to complete resorption of centrally-located trabeculae, which widens the metaphyseal marrow cavity, captured by $Tb.Sp_2$ (Figure 1C). All 3 types of Tb.Sp were different ($p<0.01$), indicating that both trabecular thinning/thickening and marrow cavity expansion contribute to Tb.Sp. In the 62-weeks-old dataset $Tb.Sp_2$ became dominant (Figure 1B). Similar results were observed for ovariectomy and spinal cord injury models. We envisage that this methodological tweak should enable a more sensitive distinction of skeletal phenotypes.

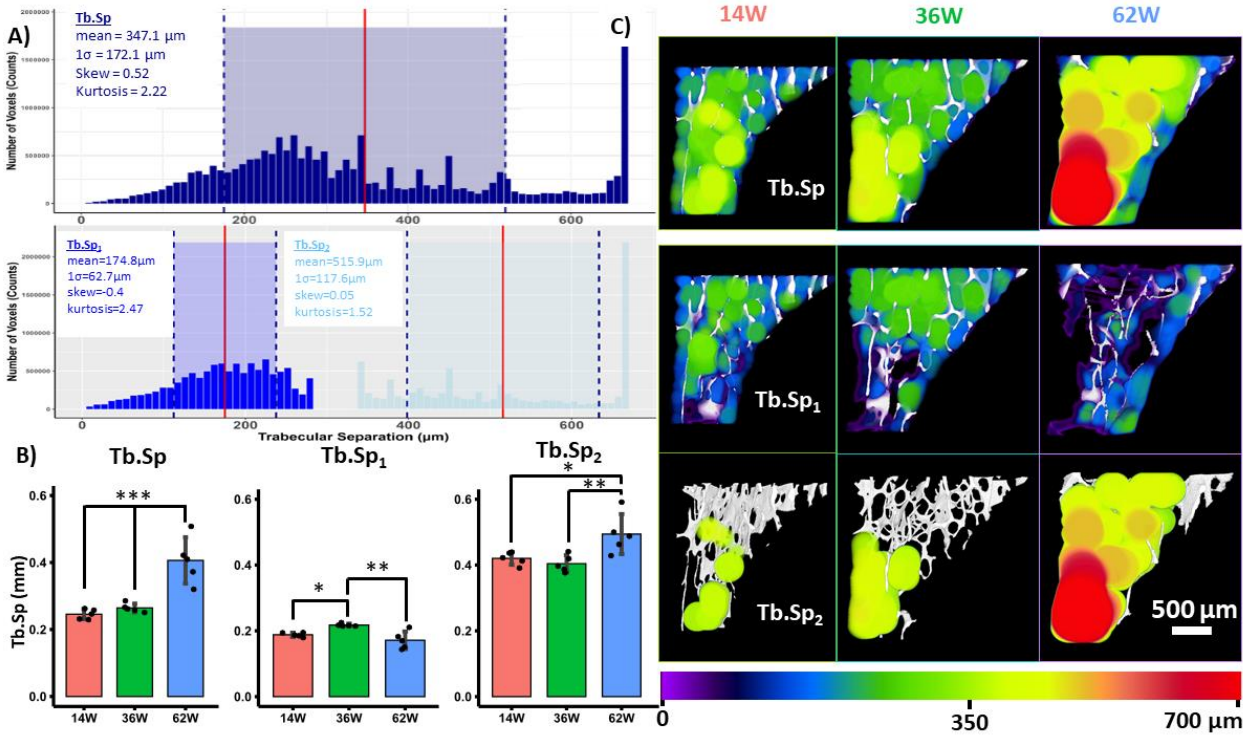

Figure 1. Trabecular separation (Tb.Sp) results for proximal tibial metaphyseal trabecular bone in the mouse ageing model. A) Representative osteoporotic Tb.Sp histogram with statistics, followed by $Tb.Sp_1$ and $Tb.Sp_2$ which provide information on intra-trabecular thinning and metaphyseal marrow cavity expansion. B) Tb.Sp morphometric analyses. C) Tb.Sp colour thickness maps, illustrating the 3 types of Tb.Sp.

### P41

### Radiographic image improvement for a better understanding of bone structure

Hassan Alshamrani[1], Khalaf Alshamrani[1,2]

[1]Najran University, Najran, Saudi Arabia [2]University of Sheffield, Sheffield, UK

### Abstract

Radiographic imaging using X-rays is one of the most important and readily available imaging types for bone. However, the quality of the radiographic images may not be as good as advanced imaging technology such as Magnetic Imaging Resonance (MRI) or Computed Tomography (CT). Nevertheless, by improving the contrast of the radiographic image, more clinical information can be obtained from these radiographic images, which makes them more useful for evaluating different bone diseases.

the histogram equalization approach was applied to a dataset of bone radiographs in order to improve the contrast of the image while the gamma correction approach was used to improve the brightness of the image. The sharpness of the image was improved using the Gaussian derivatives approach. In addition, different AI techniques were applied to boost the image quality. It is hoped that all these improvements will resolve the bone microstructure.

The morphological techniques preserve image edges, improve the bone boundaries, and better visualize image edges. A very high average of projected foreground pixels (92%) was found, which means that it is quite excellent at foreground segmentation The overall image quality was boosted. Resolving the bone microstructure and macrostructural was possible with the aid of neural network and deep learning techniques after improving the image quality.

In conclusion, applying different methods for improving bone radiographs and using AI can help greatly in gaining insightful information of the radiographic image and may reduce the need for advanced imaging techniques.

### P42

### Cardiovascular risk assessment before prescribing Romosozumab: An evaluation of the European Society of Cardiology (ESC) Systematic Coronary Risk Evaluation 2 (SCORE2) versus QRISK3

Fiona Macrae[1], Emma M Clarke[2], Matthew Roy[3], Clare Cockill[4], Katherine Walsh[2], Sarah-Jane Bailey[2], Sarah Hardcastle[5], Jon Tobias[2], Benjamin Faber[2]

[1]Gloucestershire Royal Hospitals NHS Foundation Trust, Gloucester, UK [2]North Bristol Trust, Bristol, UK [3]University Hospitals Bristol and Weston, Bristol, UK [4]Somerset Foundation Trust, Yeovil, UK. [5]Royal United Hospitals Bath, Bath, UK

### Abstract

**Objective**: Romosozumab is a promising new drug for women with severe osteoporosis. Concern exists that Romosozumab increases the risk of cerebrovascular and cardiovascular events. It is possible to assess cardiovascular risk using both the QRISK3 score and ESC SCORE2. Both scores give clinicians a 10-year risk (%) of developing a cardiovascular event. In addition, QRISK3 gives a relative risk whereas the ESC SCORE2 gives a colour grading (Green = low risk, Amber = moderate risk, or Red = high risk) based on the 10-year risk compared to a healthy age matched group. To improve clinician confidence in prescribing this drug, the Southwest Bone Group have developed a guideline for prescribing Romosozumab. Due to uncertainty around which cardiovascular risk scoring tool to use, we aimed to analyse how both scores compared in a clinical population.

**Methods**: Four osteoporosis centres across the Southwest of England prospectively collected the QRISK3 score and the ESC SCORE2 on patients they were considering for Romosozumab over a 6-month period in 2023. The difference between 10-year risk predicted by both scores was compared using a paired t-test.

**Results**: We collected data from 51 patients of which 41 had both ESC SCORE2 and QRISK3 scores assigned. All patients were female. The average age was 71y/o, youngest patient 54y/o, oldest 87y/o. Of the 41, 39 went on to be prescribed Romosozumab. The average 10-year risk (%) for QRISK3 was higher than for ESC SCORE2 (15.87 vs 8.20, $p<0.0001$). Using SCORE2, 18 were classed as 'low risk', 18 were 'moderate risk' and 5 were 'high risk.' By applying the same rules to convert a QRISK3 10-year risk (%) to equivalent groups, 4 patients would be deemed 'low risk' and 25 'high risk.'

**Conclusions**: Our study demonstrates that when assessing cardiovascular risk in the patient cohort being considered for Romosozumab, QRISK3 consistently predicts a higher risk of a cardiovascular event than ESC SCORE2. Applying risk criteria designed in ESC SCORE2 to QRISK3 scores leads to different risk labels being applied to patients. It is thought that ESC SCORE2 is more applicable to UK populations than QRISK3 based on how the score was derived. This may lead to QRISK3 inappropriately labelling patients are high risk. Guidance on the cardiovascular risk assessment of osteoporosis patients should be linked to a specific scoring system as they are not interchangeable.

### P43

### Archaeometabolomics of osteoarchaeological material and the analysis of tobacco use behaviour in pre-modern populations

Sarah Inskip[1], Diego Badillo Sanchez[2], Anna Davies-Barrett[1], Maria Serrano Ruber[3], Donald Jones[1]

[1]University of Leicester, Leicester, UK [2]Independent, Bogata, Colombia [3]UCD, Dublin, Ireland

### Abstract

Metabolomics is a rapidly expanding discipline that has had a significant impact on medical, environmental and pharmaceuticals fields by providing the opportunity to assess phenotype in dynamic systems. However, there has been almost no research in bioarchaeology and very little research on human bone in general. This research aims to assess the potential of metabolomics to reveal information about past behavioural practices and health through the analysis of archaeological human skeletal remains. Here we assess whether it is possible to obtain metabolites from archaeological human bone and whether significant metabolome difference exists between individuals with a known behaviour variable, tobacco use, which is a significant risk factor for many diseases that affect bone. In archaeological skeletons it is possible to see some evidence for tobacco use from characteristic staining on the dentition coupled with damage to the teeth from clay pipe use. We sampled 40mg of cortical bone from the femora of 302 British archaeological skeletons dating between 1500 to 1850 for an untargeted metabolomic assay. These individuals were split into three groups based on tobacco use status (pre-tobacco control group, tobacco using individuals and unknown tobacco use). Metabolites were extracted using a multistep liquid–solid process and analysed using high-flow-UPLC-IM-TOF-HRMS. Comparison of the metabolomes of pre-tobacco and known tobacco users using PCA and PLS-DA show clear separation between the groups, with 45 molecular features having a variable influence on projection (VIP) greater than 1 demonstrating them to be potential markers for tobacco use discrimination. These features were used in a linear support vector (ROC-SVM) to classify unknown tobacco users. Training models indicated a minimal misclassification rate and high sensitivity. A c.a. 9% error was found according to the pre-tobacco validation group samples which may be explained by issues of archaeological dating. Overall, this research demonstrates that it is possible to retrieve insightful metabolites from archaeological human bone, and these can be informative about past behavioural practices. The retrieval of such information potentially expands our ability to assess the impact of certain behaviours in non-modern populations, as well as assess the long-term trajectories of health and disease. Future work will focus on biomarker validation.

### P44

### Lysyl oxidase-mediated intermolecular crosslinks fine-tune collagen molecular structure in bone

Scott Dillon, Melinda Duer

University of Cambridge, Cambridge, UK

### Abstract

Bone mechanical properties are dependent on the molecular structure of collagen and the collagen-mineral interface. Lysyl oxidase (LOX) mediates intermolecular crosslinks between triple helices in collagen fibrils. However, the regulation of fibril molecular structure, its mechanical dynamics and mineral-matrix interface by crosslinks remains unexplained.

LOX overexpression in MC3T3-E1 osteoblast-like cells (LOX-OX) was achieved by insertion of full-length *Lox* cDNA and blasticidin resistance downstream of the CMV promoter to piggyBac transposon sites, mediated by co-transfection of donor plasmid and a plasmid encoding hyperactive piggyBac transposase. Successfully transfected cells were selected under blasticidin for 14 days. LOX-OX cells demonstrated a 1.8-fold mean upregulation ($p < 0.01$) in LOX activity using a fluorometric enzyme activity assay (Abcam). Immunofluorescence did not demonstrate any aberrant localisation of LOX in OX cells compared to controls. For production of *in vitro* matrices, confluent LOX-OX and wild-type control cells were cultured in αMEM supplemented with 10% fetal bovine serum, 1% penicillin/streptomycin and 50µg/ml L-ascorbic acid for 21 days.

The effect of LOX-mediated crosslinks on collagen structure was investigated by confocal and transmission electron microscopy, revealing increased density of collagen fibrils in LOX-OX cells, with large compact fibres. The extent of non-fibrillar tropocollagen monomers between fibrils was also reduced in LOX-OX cells.

Collagen molecular structure and dynamics were investigated by solid state nuclear magnetic resonance spectroscopy (ssNMR). Culture media were supplemented with 0.05mg/ml U-$^{13}C^{15}N$ L-glycine and 0.04mg/ml U-$^{13}C^{15}N$ L-proline to produce heavy isotope-labelled *in vitro* matrices and enable selection of collagen signals. Proton-driven spin diffusion $^{13}C$-$^{13}C$ correlation spectra demonstrated a restricted range of molecular orientations in LOX-OX matrices across all amino acid correlation signals. In addition, $Gly_{\alpha}$-$Hyp_{\gamma}$ correlations were present in control, but not LOX-OX spectra, indicating increased internuclear distances between amino acid residues in the triple helix backbone. In $Pro_{\gamma}$-$Pro_{\delta}$ correlations, both endo and exo proline ring conformations were present, while LOX-OX matrices exhibited only exo conformations, suggesting the triple helix is locked in an extended configuration. $^{1}H$-$^{13}C$ frequency-switched Lee-Goldburg heteronuclear correlation spectra revealed sharpening of sidechain correlations, indicating an increased $T_2$ relaxation time and therefore increased molecular motion in the triple helix.

Collectively these data indicate that the density of intermolecular crosslinks between tropocollagen molecules regulates the fine molecular structure of collagen fibrils and increased crosslinking induces larger fibres with a more extended triple helix conformation. Future work will investigate the biomienralisation of over-crosslinked collagen and establish the influence of collagen molecular structure on mineral interfaces.

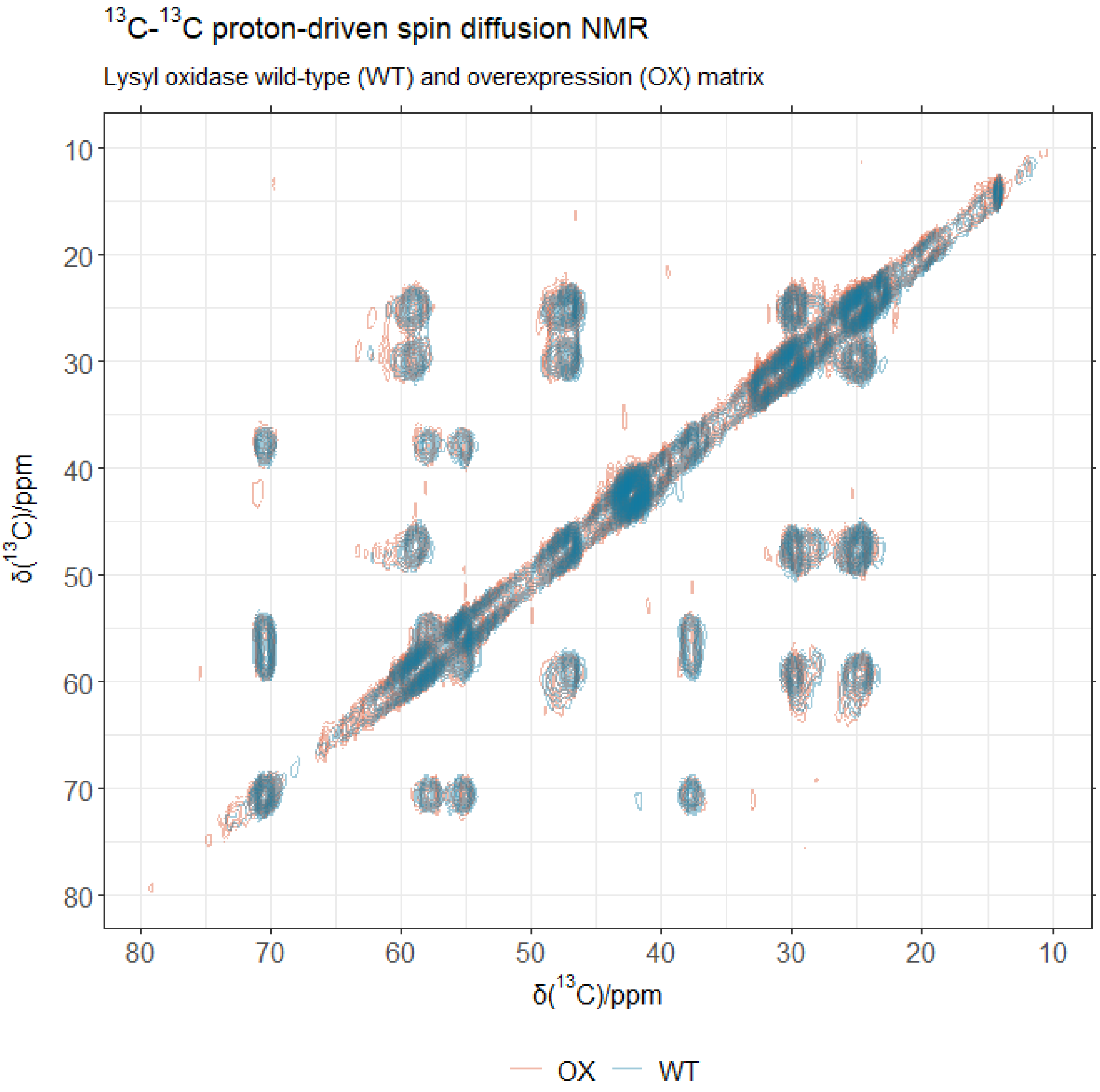

13C-13C proton-driven spin diffusion NMR
Lysyl oxidase wild-type (WT) and overexpression (OX) matrix
δ(13C)/ppm
δ(13C)/ppm
10
20
30
40
50
60
70
80
80
70
60
50
40
30
20
10
OX
WT

### P45

### An 1865 case of Phossy Jaw revisited with X-ray micro tomography and scanning electron microscopy in 2024: Growth of a new mandible after its removal

David Mills[1], Graham Davis[1], Thomas Kelly[2], Martyn Cooke[3], Alan Boyde[1]

[1]DPSU, Dental Institute, Queen Mary University of London, London, UK [2]School of Geography, Queen Mary University of London, London, UK [3]Barts Patholoy Museum, Queen Mary University of London, London, UK

### Abstract

Smith, St. Bartholomew's Hospital Reports, vol. i (1865), p. 101, reported the case of a 35-year-old male phosphorus-match-factory worker with necrosis of the mandible. The jaw was removed under chloroform by 'dividing it at the symphysis and dragging the two halves out separately. The dead bone came away completely denuded of soft parts and without the slightest remnant of periosteum'. 'The patient made a good recovery' and was discharged after six weeks, when he 'indulged rather freely in stimulants', and died.

At post-mortem, Smith reported the discovery of a newly formed jaw that lay lower in the neck than the original bone: he provided a beautiful illustration of the presumed relationships of the old and new tissues. He stated that the new bone bore much more resemblance in external outline to its predecessor before the soft parts were removed and it had dried out.

The two specimens (A.141 and A.142) were preserved in the Barts's Pathology Museum and we obtained permission to image them with x-ray micro-tomography - for which we used A Nikon HMX225 system in the Geography Dept., QMUL, 150kV, no filters, 80µm voxel resolution: and by SEM – using a Zeiss EVO10, 20kV, backscattered electrons (BSE), uncoated, 50Pa chamber pressure.

The half-mandibles removed at operation felt remarkably dense. A substantial flange of 'new bone' external to the left angle of the old jaw was not commented by Smith. XMT showed generally that all the old dead bone was more highly mineralised than healthy modern bone. New bone in the new mandible occurred in three density classes, the third - a substantial, continuous phase - apparently having no mineral content. This corresponds to Smith's 'part of the jaw that is wholly deficient in bony structure'.

BSE-SEM showed that parts of the bone surface were clean and interpretable: we have no information regarding any cleaning treatment that may have been undertaken in making the museum specimens: they might have been boiled, but there are no signs of scraping or scratching from dissection. There are substantial regions of calcified cartilage as well as woven bone callus which are probably the parts that Smith described as like pumice, as well as highly vascular compact bone with blood vessel canals penetrating from the surface.

This study has shown that valuable information can be retrieved from museum specimens with minimal intervention using modern imaging techniques.

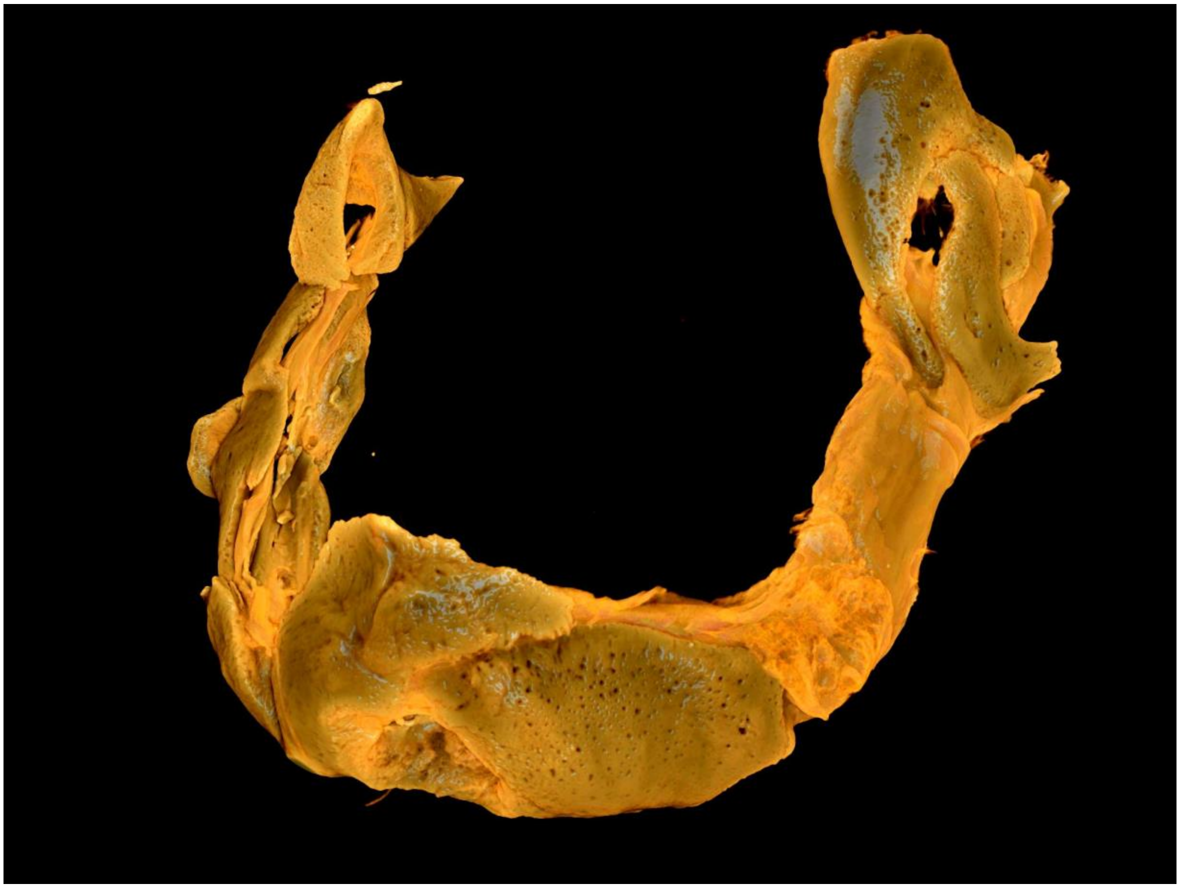

3D render of the regrown jaw ct-scan data

### P46

### Development of an electrochemical sensor to understand ROS/RNS signalling in osteoarthritis

Mohamed Habbat, Chloe Miller, Victoria Jarvis, Ana Tendero Canadas, Lucie Bourne, Bhavik Patel, Katherine Staines

University of Brighton, Brighton, UK

### Abstract

Increased reactive oxygen and nitrogen species (ROS/RNS) have been well-established to disrupt articular cartilage homeostasis and drive its degradation in osteoarthritis. However, monitoring ROS/RNS is a difficult analytical task due to the rapid turnover and low concentrations of these species. Various methods, biomarkers, and ROS/RNS-sensing optical probes have been devised and tested to evaluate the role of ROS/ RNS in biomedical science. However, these techniques are restricted by their lack of selectivity for specific ROS/RNS. Therefore, our aim was to develop a electrochemical microelectrode that offers the ability to simultaneously monitor the production of four ROS/RNS species ($H_2O_2$, $ONOO^-$, $NO^\bullet$, and $NO_2^-$) in chondrocytes. This will enable a better understanding of the contribution of ROS/RNS species in cartilage health and in diseases such as osteoarthritis. Microelectrodes were fabricated using multiwall carbon nanotube (MWCNT) and platinum black and characterised using $H_2O_2$ and $NO_2^-$ . These electrodes were tailored to directly detect specific ROS/RNS species in: (i) individual murine *ex vivo* articular cartilage hip cap explants from 4-week-old male C57/BL6 mice, and (ii) individual embryonic (E)17 murine metatarsal bones. Amperometric detection of increasing concentrations of $H_2O_2$ and $NO_2^-$ indicated successful production of electrodes with reproducible kinetics. These electrodes can be held at various potentials for selective detection of various ROS/RNS (namely 300 mV for $H_2O_2$, 450 mV for $ONOO^-$, 650 mV for $NO^\bullet$, 850 mV and $NO_2^-$). ROS/RNS detection in articular cartilage hip cap explants and metatarsal bones revealed a significant decrease hydrogen peroxide in the articular cartilage explants ($P<0.05$), whereas peroxynitrite ($P<0.05$), nitric oxide ($P<0.001$), and nitrogen dioxide ($P<0.001$) were all significantly increased. We also showed that nitric oxide synthase inhibitor (L-NAME) can reduce the responses in nitric oxide and nitrogen dioxide ($P<0.001$) observed from a single E17 metatarsal bone, highlighting our sensor's specificity in monitoring these RNS markers. Overall, our findings highlight that MWCNT electrodes provide a sensitive detection of ROS/RNS species in *ex vivo* cartilage explants. This will enable future investigation into the role of specific ROS/RNS in chondrocyte behaviours in health and disease.

### P47

### Effect of a bout of cricket fast bowling on bone resorption

Zhuoyue Zhang[1], Peter Alway[1,2], Faye Harper[1], Beca Morgan[1], Buzz Kemp[1], Ciaran Beaumont[1], Ogulcan Caliskan[1], Katherine Brooke-Wavell[1]

[1]School of Sport, Exercise and Health Sciences, Loughborough University, Loughborough, UK
[2]Department of Science and Medicine, England and Wales Cricket Board, Loughborough, UK

### Abstract

**Background:** Cricket fast bowling subjects the lumbar vertebrae to extremely high forces and when coupled with high relative short term bowling volumes, puts fast bowlers at risk of lumbar spine bone stress injuries. The bone strains experienced by fast bowlers are likely above the modelling threshold, and coupled with high bowling volumes, results in accumulation and propagation of microdamage. This microdamage may promote an increase in bone resorption that may contribute to stress fracture aetiology.

**Purpose:** To determine whether an acute bout of cricket fast bowling affects serum carboxy-terminal crosslinking telopeptides of type I collagen (CTX), a marker of bone resorption.

**Methods:** A controlled trial was conducted in 12 male University Centre of Cricket Excellence fast bowlers. Participants completed the bowling and control trials separately, with a one-week interval between the two trials. In the bowling trial, participants completed 8 sets of 6 deliveries (bowl at match intensity throughout), followed by 2-hour rest. Each set was interspersed by a 3-minute randomised fielding simulation. In the control trial, participants rested throughout instead of bowling. Time of day and prior diet and exercise were matched between trials. Time-matched blood samples were collected three times in each visit: pre-, immediately post and 2-hour post bowling/rest and analysed by ELISA (serum crosslaps, IDS Ltd). Paired t-tests detected the difference in means. Repeated measures analysis of variance (ANOVA) examined the effect of time, trial, and time×trial interaction.

**Results:** Twelve male participants (mean±SD; age 19.6±1.3 years; height 189.5±7.1 cm; body fat 14.8±4.2%) completed the study. There was no difference in body mass between trials. Baseline CTX didn't differ between the bowling trial (0.53±0.24 ng/mL) and control trial (0.56±0.31 ng/mL, P=0.772). There was a significant effect of time (P=0.009), but no effects of trial (P=0.886) or time×trial interaction (P=0.249). In the control trial there was a significant effect of time (P=0.026): CTX decreased by 24.2% during the initial rest, and then increased by 59.7% over the subsequent 2 hours. There were no significant changes over time in the bowling trial (P=0.506).

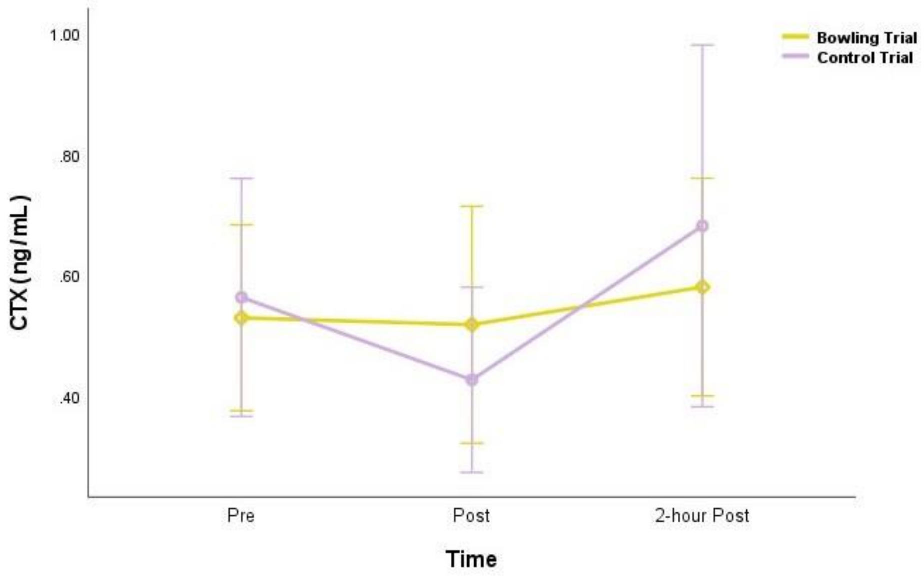

**Conclusion:** Significant diurnal variation in bone resorption, with a decline and subsequent increase, was observed in the control but not bowling trial. The study may be underpowered to detect a difference between trials but the lack of diurnal decline in CTX in the bowling trial may represent a transient increase in bone resorption following the bout of fast bowling.

### P48

### The interplay between haematological disorders and osteoporosis: Evaluating the risks

Marwa Mohareb, Kanishk Jain, Anupama Nandagudi, Anurag Bharadwaj

Mid and south Essex hospitals, Basildon, UK

### Abstract

Background:

Secondary osteoporosis (OP) is a frequent clinical concern, sometimes attributed to underlying haematological diseases. These conditions can either be previously known or identified through routine evaluations. Notably, multiple myeloma and monoclonal gammopathy of undetermined significance (MGUS) are amongst the secondary causes of OP.

Objectives:

To determine the prevalence of monoclonal gammopathies in an inception cohort of patients diagnosed with osteoporosis and osteopenia.

Methods:

It is a retrospective study of 117 patients with low bone density (2 were excluded) between January and June 2022. We gathered data encompassing age, gender, DXA scan referral reasons, any history of fragility fractures and secondary risk factors linked to osteoporosis.

As part of our evaluation, we conducted tests to detect monoclonal gammopathy. This involved performing a serum protein electrophoresis (SPEP), a serum free light chain (sFLC) assay, and a urine Bence Jones protein (uBJP) test, alongside standard blood tests designed for bone density assessment.

Results:

Our cohort consisted of 102 females (88.7%), with ages ranging from 47 to 90 years (median age: 69 years). Among this cohort, 47% were diagnosed with osteopenia, 53% with osteoporosis, and 59% had a history of fragility fractures.

The primary reason for referrals to DXA scans was low-trauma fractures (54.8%), radiological evidence of osteopenia (22.6%), hyperparathyroidism (7.8%), breast cancer (6.1%), and premature menopause (5.2%).

Significant risk factors for osteoporosis were categorized based on their prevalence percentages. The most notable factors were female gender (88.7%) history of low-trauma fractures (55.7%) premature menopause (25.2%), parental hip fracture (24.3%), proton pump inhibitor use (16.5%) .

Table: classification according to laboratory results.

| | No. (%) |
|---|---|
| **sFLC ratio** | Normal 106 (92.2%) |
| | Abnormal 2 (1.7%) |
| | ND 7(6.1%) |
| **sPEP** | Normal 108 (93.9%) |
| | Abnormal 1 (0.9%) |
| | ND 6 (5.2%) |
| **uBJP** | Normal 95 (92.2%) |
| | Abnormal 1 (0.9%) |
| | ND 19 (16.5%) |

ND: not done

Notably, we identified two patients with abnormal test results. Patient 1, an 80-year-old female with osteopenia, exhibited an elevated Kappa/Lambda ratio and a positive uBJP, resulting in a diagnosis of multiple myeloma after presenting with fractures. Patient 2, a 60-year-old female with osteoporosis, displayed IgM paraproteinemia and a low Kappa/Lambda ratio, leading to a diagnosis of low-grade B cell non-Hodgkin's lymphoma following a humeral fracture and hyperparathyroidism.

Conclusion:

Our screening revealed the absence of MGUS, with only isolated instances of multiple myeloma and B cell lymphoma among patients with osteoporotic fractures. The study highlights the significance of recognizing haematological abnormalities in patients with osteoporotic fractures and the importance of early referral for appropriate diagnosis and management. The investigation of monoclonal gammopathy should be pursued further to better understand its potential connection to osteoporosis.

### P49

### Effectiveness and safety of bone-protective interventions to mitigate bone loss and skeletal-related events experienced by patients with non-metastatic breast cancer: A systematic review protocol

Micaela Quinn, Tania Crotti, Bonnie Williams, Joanne Bowen

The University of Adelaide, Adelaide, Australia

### Abstract

**Background:** Patients receiving antineoplastic treatment for breast cancer are well-known to experience accelerated bone loss, a phenomenon termed cancer treatment-induced bone loss (CTIBL) and associated skeletal-related events. There are a range of bone-protective interventions clinically approved for use in this patient population that aim to mitigate bone loss and subsequent skeletal-related events, such as bisphosphonates and denosumab. However, the effectiveness and safety of these interventions in the setting of CTIBL in non-metastatic breast cancer remains inconclusive.

**Purpose:** This research aims to evaluate the effectiveness and safety of bone-protective interventions used to mitigate bone loss and skeletal-related events experienced by patients receiving antineoplastic treatment for non-metastatic breast cancer.

**Inclusion Criteria:** This systematic review will consider studies investigating the use of bone-protective interventions, specifically bisphosphonates (alendronate, clodronate, ibandronate, pamidronate, risedronate and zoledronate), denosumab, and calcium and vitamin D supplementation, to mitigate bone loss and skeletal-related events. Studies featuring patients aged ≥18 years undergoing mono- or combination antineoplastic therapy for non-metastatic breast cancer will be considered. Pre- and post-menopausal patients within both the hospital and community setting are to be considered in this review. Both experimental and quasi-experimental studies, as well as analytical observational studies will be included, whilst preclinical studies and reviews will not be included.

**Methods:** A comprehensive search will be undertaken using MEDLINE/PubMed, Embase, Emcare, CINAHL, Cochrane Central Register of Controlled Trials and Google Scholar, with gray literature also systematically searched. In accordance with Joanna Briggs Institute methodology, studies will be screened by title and abstract, and full-text and then critically appraised by two independent reviewers, with any disagreements to be resolved by the inclusion of an additional reviewer. Data will then be extracted and synthesised, with findings presented in a meta-analysis or narrative review depending on study heterogeneity. The protocol for this systematic review is registered in PROSPERO (CRD 42022379983).

**Expected outcomes:** The skeletal morbidity experienced by patients with breast cancer will continue to rise with increasing survivorship rates, hence, understanding the effectiveness and safety of bone-protective interventions used in this patient population is critical. This research will provide an assessment of the level of evidence for the effectiveness and safety of the bone-protective interventions used in the non-metastatic breast cancer setting to mitigate CTIBL and associated skeletal-related events. Further, it will contribute to the growing body of knowledge regarding the importance of bone health in the supportive cancer care setting, thereby enhancing clinical decision making.

### P50

### Osteoblasts sense systemic levels of phosphate to control the local expression of phosphatases during matrix mineralisation

Soher Jayash, Thomas Duff, Qaisar Tanveer, Colin Farquharson

The Roslin Institute and Royal (Dick) School of Veterinary Studies, University of Edinburgh, Edinburgh, UK

### Abstract

The provision of inorganic phosphate for biomineralisation of the skeleton is under both systemic and local control. Circulating levels of vitamin D and FGF23 control phosphate levels and a deficiency of the former results in bones that are poorly mineralized and easily fractured. Locally, osteoblast production of phosphatases such as tissue-nonspecific alkaline phosphatase (TNAP) and PHOSPHO1 is required for physiological mineralisation whereas matrix proteins such as osteopontin and matrix Gla protein are recognised inhibitors of mineralisation. It is possible that osteoblasts may sense phosphate concentrations in their extracellular environment to control local phosphatase activity and “fine-tune” phosphate delivery to the collagenous matrix. This however has been poorly explored and this project examined the ability of osteoblasts to sense extracellular Pi concentrations and control the local production of TNAP and PHOSPHO1.

Human primary osteoblasts were cultured with varying phosphate concentrations (0, 1mM, 3mM & 5mM), and matrix mineralisation (alizarin red staining) and PHOSPHO1, *ALPL*, *SLC20A1*/PiT1, and *SLC20A2/*PiT2 expression (RT-qPCR and immunoblotting) were examined. Immunoblotting with antibodies to total and phosphorylated forms of ERK1/2, AKT, p38, and JNK1/2 was employed to detect intracellular signalling pathways. Additionally, specific inhibitors of MEK 1/2 (U0126), FGFR1 (PD173074) and Pit1/Pit2 (phosphonoformic acid) were introduced to the cultures to help explore the intracellular signalling pathways activated by extracellular phosphate.

Increasing extracellular phosphate promoted osteoblast matrix mineralisation whereas the expression of PHOSPHO1 and TNAP was down-regulated in response to increasing phosphate concentrations at both the mRNA and protein levels ($p<0.05$). Extracellular phosphate also downregulated PiT-1 and PiT-2 expression at the mRNA level ($p<0.05$), but enhanced the phosphorylation of ERK1/2 but not AKT, JNK1/2, or p38. The phosphorylation of ERK 1/2 by extracellular phosphate was suppressed by phosphonoformic acid. Co-treatment with U0126 or phosphonoformic acid abolished the decrease in *PHOSPHO1 and ALPL* expression by elevated extracellular phosphate. Inhibition of FGFR1 also reduced the ability of extracellular phosphate to phosphorylate ERK1/2

These results demonstrate that extracellular phosphate is sensed by osteoblasts and promotes matrix mineralisation but down-regulates both TNAP and PHOSPHO1 expression. The inhibition of ERK1/2 activation by extracellular phosphate by either phosphonoformic acid or PD173074, infers a potential cooperation of the FGFR1 receptor and the Pit1/Pit2 co-transporters in phosphate sensing by osteoblasts. These findings contribute to a better understanding of the molecular events associated with the control of osteoblast matrix mineralisation and suggest that the local production of phosphate by bone forming osteoblasts may “fine-tune” physiological bone mineralisation.

### P51

### Tumour associated macrophages increase metastasis of osteosarcoma cells

Daniel Doro, Marco Krstic, Michael Dack, Agamemnon E Grigoriadis

King's College London, London, UK

### Abstract

Osteosarcoma (OS), the most common malignancy of the bone, remains a burdensome health issue, affecting mostly children and young adults. The 5-year relative survival rate is 60-70% for the primary disease, but only 20-30% in cases of advanced metastasis. Recently, the role of tumour associated macrophages (TAMs) has gained significant attention: Heme Oxygenase-1 (HO-1)-expressing M2 macrophages have been correlated with increased tumour progression and metastasis in breast cancer. Here we investigate the role of TAMs in osteosarcoma as well as the correlation between HO-1 expression and tumour progression.

We have used a mouse model of osteosarcoma overexpressing the proto-oncogene c-Fos to investigate the presence of M2 (CD206+) macrophages and the expression of HO-1 in different stages of tumour progression. Immunostaining analysis showed that both early and late-stage tumours are rich in CD206+ M2 macrophages and HO-1 is largely expressed in the marrow of tumour-bearing long bones. To investigate whether transformed osteoblasts could induce an M2 profile in bone marrow-derived macrophages (BMMs), we co-cultured mouse BMMs with either mouse (131-OS2/P1.15) or human (MG63/MNNG-HOS) OS lines and showed that OS cells induced expression of the M2 marker Arg1 at the expense of iNOS (M1 marker) expression, while macrophages cultured with non-transformed osteoblasts display very low expression of both M1 and M2 markers.

To investigate the effect of TAMs in OS tumour growth and metastasis we co-cultured GFP+ MG63 cells and Tomato+ BMMs in three-dimensional spheroids encapsulated in a type 1-collagen gel. Only in the presence of macrophages, the OS cells showed greater invasive and migration activity within the collagenous matrix compared to OS cells cultured alone. Moreover, grafting these spheroids onto the chorioallantoic membrane (CAM) of a 4-day chick embryo showed enhanced vascularisation of the tumour cell explants when co-cultured with mouse BMMs. Strikingly, GFP+ MG63 cells were present in the chick lung and liver tissues to a significantly greater extent when MG63 were co-cultured with mouse BMMs when compared to MG63 cells alone. Overall, these results suggest a proposed model whereby OS cells induce an M2 profile in macrophages, and tumour associated macrophages are then able to increase osteosarcoma tumour growth and metastasis. Our current efforts are to investigate potential mechanisms by which macrophages confer increased invasiveness and intravasation capabilities to OS cells.

### P52

### Potential biomarkers of bone and adipose inter-tissue crosstalk in obese bariatric patients

Simona Holotova[1], Pavel Hruska[2,3], Jan Kucera[4], Daniela Kuruczova[1], Marek Buzga[5,6], Matej Pekar[7,8], Pavol Holeczy[9,10], David Potesil[3], Zbynek Zdrahal[3], Marketa Makarova[4], Julie Bienertova-Vasku[4]

[1]RECETOX, Faculty of Science, Masaryk University, Brno, Czechia [2]Dept of Pathological Physiology, Faculty of Medicine, Masaryk University, Brno, Czechia [3]Central European Institute of Technology, Masaryk University, Brno, Czechia [4]Dept of Physical Activities and Health Sciences, Faculty of Sports Studies, Masaryk University, Brno, Czechia [5]Dept of Laboratory Medicine, University hospital Ostrava, Ostrava, Czechia [6]Dept of Physiology and Pathophysiology, Faculty of Medicine, University of Ostrava, Ostrava, Czechia [7]Vascular and Miniinvasive Surgery Center, Hospital AGEL Trinec-Podlesi, Trinec, Czechia [8]Dept of Physiology, Faculty of Medicine, Masaryk University, Brno, Czechia [9]Dept of Surgery, Vitkovice Hospital, Ostrava, Czechia [10]Dept of Surgical Disciplines, Faculty of Medicine, University of Ostrava, Ostrava, Czechia

### Abstract

Interactions between obesity and the skeleton have been extensively studied; however, the results are inconsistent, partially because the effects of an excessive amount of adipose tissue on bone health are mediated by both mechanical and biochemical factors. These factors are further modulated by several variables, including adipose tissue distribution, sex, and age.

Here, we employed an untargeted approach to comprehensively identify proteins associated with bone health parameters in a cohort of obese subjects. We conducted a cross-sectional analysis of 32 patients with severe obesity (16 males and 16 females) who underwent bariatric surgery. Serum samples, along with adipose tissue samples, were subjected to proteomics liquid chromatography-mass spectrometry (LC-MS). Body composition was assessed using dual-energy X-ray absorptiometry (DXA). Correlation analyses between differentially expressed proteins and bone health parameter – Bone mineral density (BMD) total.

Of the 1,206 serum proteins, we identified 41 proteins with positive and 37 with negative correlations with BMD total. Further, we compared these proteins with potentially secreted proteins significantly upregulated or downregulated in subcutaneous and visceral adipose tissue. This resulted in the identification of six proteins with the capability for inter-tissue communication. Adipsin and Alkaline Phosphatase – Tissue-Nonspecific Isozyme (AP-TNAP) showed a positive correlation with BMD, while T-cadherin, MCAM, CD44 Antigen, and Integrin Alpha-2 (ITGA2) exhibited a negative correlation with BMD.

In conclusion, we employed state-of-the-art untargeted proteomics analysis to identify candidate proteins associated with bone health parameters in a cohort of obese subjects. Six potentially secreted proteins enriched in adipose tissue, which correlated with BMD, were identified, including Adipsin, TNAP, T-cadherin, MCAM, CD44 Antigen, and ITGA2. Further research is warranted to elucidate the functional roles of these proteins in adipose-bone crosstalk and their potential as therapeutic targets for bone health in obesity.

### P53

### Preventing periprosthetic joint infection: A novel antimicrobial sol-gel approach

Sarah Boyce[1], Christine Le Maitre[2], Thomas Smith[1], Tim Nichol[1]

[1]Sheffield Hallam University, Sheffield, UK [2]The University of Sheffield, Sheffield, UK

### Abstract

**Introduction**

Arthroplasty surgeries and their subsequent complications are increasing, including devastating periprosthetic joint infections which follow 1-3% of surgeries[1].

The biofilm nature of these infections makes them resistant to treatment and a significant challenge. Prevention strategies include antibiotic-loaded bone cement; however, with increasing cementless applications there is a need for alternative, local antimicrobial delivery methods[2].

A novel, ultrathin silica-based sol-gel technology is evaluated in this research as a localised antibiotic delivery method to inhibit infection following surgery, including its biofilm inhibition, antibiotic elution and osteocompatibility.

**Methods**

Reduction in clinically relevant microbial activity and biofilm reduction by antimicrobial sol-gel coatings, containing a selection of antibiotics, were assessed via disc diffusion and microdilution culture assays using the Calgary biofilm device[3].

Proliferation, morphology, collagen, and calcium production by primary bovine osteoblasts cultured upon antimicrobial sol-gel surfaces were examined, and cytotoxicity evaluated using Alamar blue staining and lactate dehydrogenase assays. Concentrations of silica, calcium and phosphorus compounds within the cell layer, and eluted into media, were quantified using ICP-OES. Furthermore, cellular phenotype was assessed using alkaline phosphatase activity with time in culture.

**Results**

Low antibiotic concentrations within sol-gel had an inhibitory effect on biofilm growth, for example 0.8 mg $ml^{-1}$ tobramycin inhibited clinically isolated *S. aureus* (MRSA) growth, an 8-log reduction in viable colony forming units.

There was no significant difference in metabolic activity between untreated and sol-gel exposed primary bovine osteoblasts in elution-based assays ($p= 0.81$). Reduction (1.72-fold) in metabolic activity in direct contact assays ($p= 0.04$) were likely to be due to increased osteoinduction, whereas no impact upon cell proliferation were observed ($p=0.92$ at 14 days culture). Morphology of primary osteoblasts was unaffected by culture on sol-gel and collagen production maintained, whilst calcium containing nodule production within bovine osteoblastic cells was increased 16-fold after 14 days culture upon sol-gel ($p= <0.0001$).

**Conclusions**

The ultrathin sol-gel coating showed low cytotoxicity, strong biofilm reducing activity and antimicrobial activity, which was comparable to antibiotics alone, demonstrating that sol-gel delivery of antibiotics could provide local antimicrobial effects to inhibit PJI growth without the need for bone cement. Future work will develop and evaluate sol-gel performance in an *ex vivo* explant bone infection model which, reducing need for animal experimentation.

1. P. Izakovicova; O. Borens; A. Trampuz *EFORT Open Rev.* **2019,** *4(7),* 482-494.
2. K. Garfield *et al*. *BMC Med.* **2020,***18*, 335.
3. H. Ceri *et al*. *J Clin Microbiol.* ***1999****, 37*(6), 1771–1776

### P54

### Generation and characterization of immortalised equine osteoblast cell lines

Esther Palomino Lago, Debbie Guest, Isabel Orriss, Scott Roberts

Royal Veterinary College, Hatfield, UK

### Abstract

An imbalance in bone remodelling results in several skeletal disorders. The anatomy and biology of horses lend equine model systems as an attractive animal model of human musculoskeletal injuries. *In vitro* cell models offer a promising tool for exploring diseases, understanding cellular behaviour or testing new drugs and biomaterials. Immortalised cell lines are often used in research, instead of primary cells, as a simple model for more complex biological systems. Equine osteoblasts were isolated from distal limb trabecular bone explants of a 2-year old horse using collagenase II digestion for 1 hour and subsequent outgrowth. The osteogenic nature of the cells was confirmed by assessing the expression of osteoblast-associated genes (*COL1A1, SPARC, SPP1, IBSP, RUNX2, BGALP, ALPL*) in the primary cells at passage 1. Moreover, these genes were highly expressed in osteoblasts compared with tendon, cartilage, and skin cells. For example, expression levels of *SPP1* and ALPL were 500 and 100 times higher, respectively, in osteoblasts. Once at passage 3-4, equine osteoblasts were cultured in osteogenic media (alpha MEM, 5mM β-glycerophosphate, 300mM L-ascorbic acid, 10nM dexamethasone) for 21 days. The alizarin red S assay demonstrated the ability of the cells to form mineralised matrix, with a significant fold change in staining compared to basal conditions ($p<0.01$). Positive staining for hydroxyapatite was also evident after 21 days of osteogenic culture compared with negative staining of cells cultured in basal media. An alkaline phosphatase (ALP) assay revealed an increase in enzyme activity at day 7 (average of 2.3 mU/ml) compared with day 0 (average of 0.10 mU/ml) before declining on day 21 (average of 1.19 mU/ml). After characterization, primary cells at passage 3 were immortalized using SV40-T and/or hTERT by retrovirus transduction and antibiotic selection. 10 days after selection, cells were highly diluted to obtain monoclonal cell populations. Morphology and proliferation rate was visually monitored to select up to 6 clones from each transduction. Stable integration of SV40 and/or hTERT genes was confirmed by PCR at passage 7 in all clones and all clones could be successfully frozen and thawed. Osteoblast gene expression and the ability to form a mineralised matrix in osteogenic culture was determined over subsequent passages. Continued passaging of the cells is on-going to reach a minimum of 30 passages when their properties will be confirmed again.

**P55**

*Withdrawn from publication*

### P56

### Pamidronate does not modify bone parameters in *Sost*$^{-/-}$ mice: Implications for the role of osteoclasts in sclerosteosis

Jacob A C Keen[1], Timothy J Dreyer[1], Gill Holdsworth[2], Andrew A Pitsillides[1], Scott J Roberts[1]

[1]The Royal Veterinary College, London, UK [2]UCB Pharma, Slough, UK

### Abstract

Sclerostin, encoded by the *SOST* gene, is a negative regulator of bone formation whilst mediating bone mechanotransduction. Mature osteocytes secrete sclerostin, binding to receptors LRP4/5/6 of osteoblasts, thus inhibiting Wnt/β-catenin signalling. *SOST* gene mutations result in heightened bone mass as observed in Sclerosteosis, with *Sost*$^{-/-}$ mice effectively recapitulating this disease.

Aberrant bone formation in the *Sost*$^{-/-}$ mouse has been reported to be remodelling based, with inhibition of bone resorption via treatment with anti-RANKL in *Sost*$^{-/-}$ mice resulting in suppression of bone formation (Koide et al 2022). As such we hypothesise that inhibiting osteoclast activity may potentially decrease bone formation in *Sost*$^{-/-}$ mice, analogous to that observed with anti-RANKL. Bisphosphonates function by promoting osteoclast apoptosis, with pamidronate disodium previously being utilised in paediatric cases for treatment of osteogenesis imperfecta.

Pamidronate's efficacy was validated using primary osteoclast assays from C57/BL6 and *Sost*$^{-/-}$ mice cultured for 7 days on dentine discs. To test therapeutic potential, 6-week-old male *Sost*$^{-/-}$ mice (n=10) received two subcutaneous injections per week for two weeks of 2mg/kg pamidronate disodium (Chen et al 2019) or PBS, and underwent mechanical bone loading of the right tibia at a peak of 20N during the two weeks of treatment, mimicking loading events during childhood. Mice were then maintained for 6 weeks, and microarchitecture of the tibia and vertebrae analysed by microcomputed tomography (µCT) using CTan (Bruker, Belgium).

Whole tibial analysis showed no significant differences ($p$>0.05) between treatment groups in either the trabecular or cortical bone in both the loaded and non-loaded limbs (mid-point bone area: vehicle non-loaded = 0.293mm$^2$, vehicle loaded = 0.308mm$^2$, treated non-loaded = 0.299mm$^2$, treated loaded = 0.295mm$^2$). Analysis of the cortical (bone area, $p$=0.203; tissue area, $p$=0.2686; bone area/tissue area ratio, $p$=0.1232) and trabecular (bone volume, $p$=0.3694; tissue volume, $p$=0.6687; bone volume/tissue volume ratio, $p$=0.0535; trabecular thickness, $p$=0.0630; trabecular spacing, $p$=0.1842; trabecular number, $p$=0.1635) regions of the vertebrae also showed no significant change, suggesting that 2mg/kg subcutaneous injection of pamidronate disodium is not sufficient to supress bone formation in the *Sost*$^{-/-}$ mouse.

In summary, converse to published reports suggesting anti-RANKL suppressed bone formation in *Sost*$^{-/-}$ mice, administration of the osteoclast mediator pamidronate disodium, resulted in no change in bone parameters. Although pamidronate disodium had no effect alone within this study, the bisphosphonate could be evaluated in the future in combination with small molecules as a targeting moiety for treatment of Sclerosteosis.

### LB1

### Bone surface models: A new tool to accurately categorize elbow osteoarthritis patients

Taqwa Abusalem[1,2], Alexei Zhurov[1], Tim Matthews[3], Manhal Agha[4], Hannah Shaw[2], Sharon Dewitt[5], Daniel Aeschlimann[6]

[1]Matrix Biology & Tissue Repair Research Unit, School of Dentistry, College of Biomedical and Life Sciences, Cardiff University, Cardiff, UK [2]School of Biosciences, College of Biomedical and Life Sciences, Anatomy Centre, Cardiff University, Cardiff, UK [3]Dept of Trauma & Orthopaedics, Cardiff & Vale NHS Trust, University Hospital of Wales, Heath Park, Cardiff, UK [4]Dept of Trauma & Orthopaedics, Cardiff & Vale NHS Trust, University Hospital of Wales, Heath Park, Cardiff, UK [5]Matrix Biology & Tissue Repair Research Unit, School of Dentistry, College of Biomedical and Life Sciences, Cardiff University, Cardiff, UK [6]Matrix Biology & Tissue Repair Research Unit, School of Dentistry, College of Biomedical and Life Sciences, Cardiff University, Cardiff, UK

### Abstract

**Keywords:** Elbow, Osteoarthritis, Three-dimensional, Statistical-models, Diagnosis.

**Background.** Elbow osteoarthritis (OA) is a debilitating condition known for its painful symptoms, limited range of motion, and potential for joint locking. A distinctive manifestation of this condition is extensive osteophyte formation around the joint margins. To date, elbow OA remains challenging to treat due to the lack of early diagnostic tests. We aim to categorise elbow OA patients using an unbiased approach based on three-dimensional surface models.

**Study design**. CT elbow data from osteoarthritic (n=9) and healthy males were processed to extract three-dimensional (3D) surface images of elbow bones. Participant CT scans were acquired following informed consent under Wales REC3 10/MRE09/28:AR UK BBC Multi-project ethical submission. 3D surfaces of the humerus, radius and ulna of cadaveric bones were scanned, scaled and aligned to enable comparison (n=30, SREC 23 09-01). We averaged the respective data sets using an in-house software algorithm to obtain 3D statistical models. Model validation was performed against standard 3D CT images of non-OA individuals. These models were subsequently employed to evaluate and categorise differences in OA patients.

**Results**. Using 15 to 18 individual bone data sets was adequate to generate an accurate statistical model through averaging. Further data incorporation resulted in negligible change, less than 0.05 mm on average across the entire surface, considering that CT scan accuracy is ~0.5 mm. The range of normal variation in non-OA bones (statistical models) was determined to be 3.8 mm (humerus), 1.5 mm (radius) and 4.2 mm (ulna) for 99.7% of the bone surface. Given this, we set a 4mm threshold for both the humerus and ulna and 1.5 mm for the radius to identify OA-related changes, followed by generating colour maps to visualise these changes. Additionally, osteophyte location and size tables were created for all patients to identify patterns in the OA changes. Furthermore, we categorised our patients using two distinct methods: one based on the distance between specific landmarks and the other utilised the surface area affected by the osteophyte. Both categorisation techniques complement each other, offering a comprehensive assessment of osteophyte severity.

**Conclusion.** The adopted approach has proven instrumental in characterising unique changes in the elbow joint that are associated with a specific aetiology. This information not only enhances our understanding of elbow osteoarthritis but also holds the potential for facilitating early diagnosis and more accurate disease course prediction, thereby improving patient outcomes in clinical practice.

### LB2

### Raman spectroscopy identified nail compositional differences between sexes but not between hands and fingers

Nai-Hao Yin[1], Frances Griffiths[2], Helen Dawes[3], Richard van Arkel[4], Jemma Kerns[1]

[1]Lancaster University, Lancaster, UK [2]The University of Warwick, Warwick, UK [3]University of Exeter, Exeter, UK [4]Imperial College London, London, UK

### Abstract

The structural protein keratin in nails can show similar changes to the collagen in bones. Combining with spectroscopy, it has been proposed that measuring the compositions of nails, an easily accessible tissue, can be a feasible approach to screen people at risk of developing osteoporosis. Raman spectroscopy is a light-based technique sensitive to structural changes in materials and has been applied on human fingernails to identify differences between healthy and osteoporotic patients. While this approach is promising for screening bone health at the population level, there is a lack of basic knowledge about differences within and between individuals (e.g. sex or age). Specifically, previous studies have focused on females with nails clipped from one randomly selected finger. Therefore, the aim of this study was to test the hypothesis that there are no spectral, therefore chemical, differences within one individual, but that there will be differences due to sex.

Five males (24 – 38 yrs) and females (25 – 38 yrs) without known dermatological or musculoskeletal diseases donated fingernail clippings from all fingers. Nail clippings were gently cleaned with an acetone-based solution and left to dry prior to Raman spectroscopy measurements (785nm, 300mW at source) using standardised protocols. Spectral data (450 – 1800cm$^{-1}$) were then baseline corrected with polynomials and normalised to phenylalanine peak before subjected to principal component analysis (PCA) and univariate analysis to compare differences between fingers and between sexes.

In total 500 spectra were collected and analysed. Sex differences were identified by PCA and group means (Fig.1A). Males have significantly higher intensity than females at bands 591cm$^{-1}$ (0.23±0.13 vs. 0.19±0.08), 1063cm$^{-1}$ (0.34±0.11 vs. 0.28±0.07), 1295cm$^{-1}$ (0.78±0.19 vs. 0.71±0.14), and 1450cm$^{-1}$ (1.41±0.20 vs. 1.28±0.13), indicating different keratin structure or total protein content exist between sexes. There were no significant differences between left and right hands (Fig.1B) and between fingers.

This pilot study demonstrates that nail compositions, measured by Raman spectroscopy, are similar between hands/fingers and could imply that handedness, habitual, sport or occupational hand usage may have little influence on the nail keratin structure. However, despite the small sample size, our results suggest that significant sex differences could exist in nail compositions among healthy individuals. Future studies using nails as a surrogate tissue for measuring bone health, such as osteoporosis, at the population level should be done with care since fundamental compositional difference may exist between sexes.

(A)
Sex difference
Male
Female
Absorbance (a.u.)
Wavenumber (cm-1)
1.6
1.4
1.2
1
0.8
0.6
0.4
0.2
0
1800
1600
1400
1200
1000
800
600
(B)
Hand difference
Left
Right
Absorbance (a.u.)
Wavenumber (cm-1)
1.6
1.4
1.2
1
0.8
0.6
0.4
0.2
0
1800
1600
1400
1200
1000
800
600

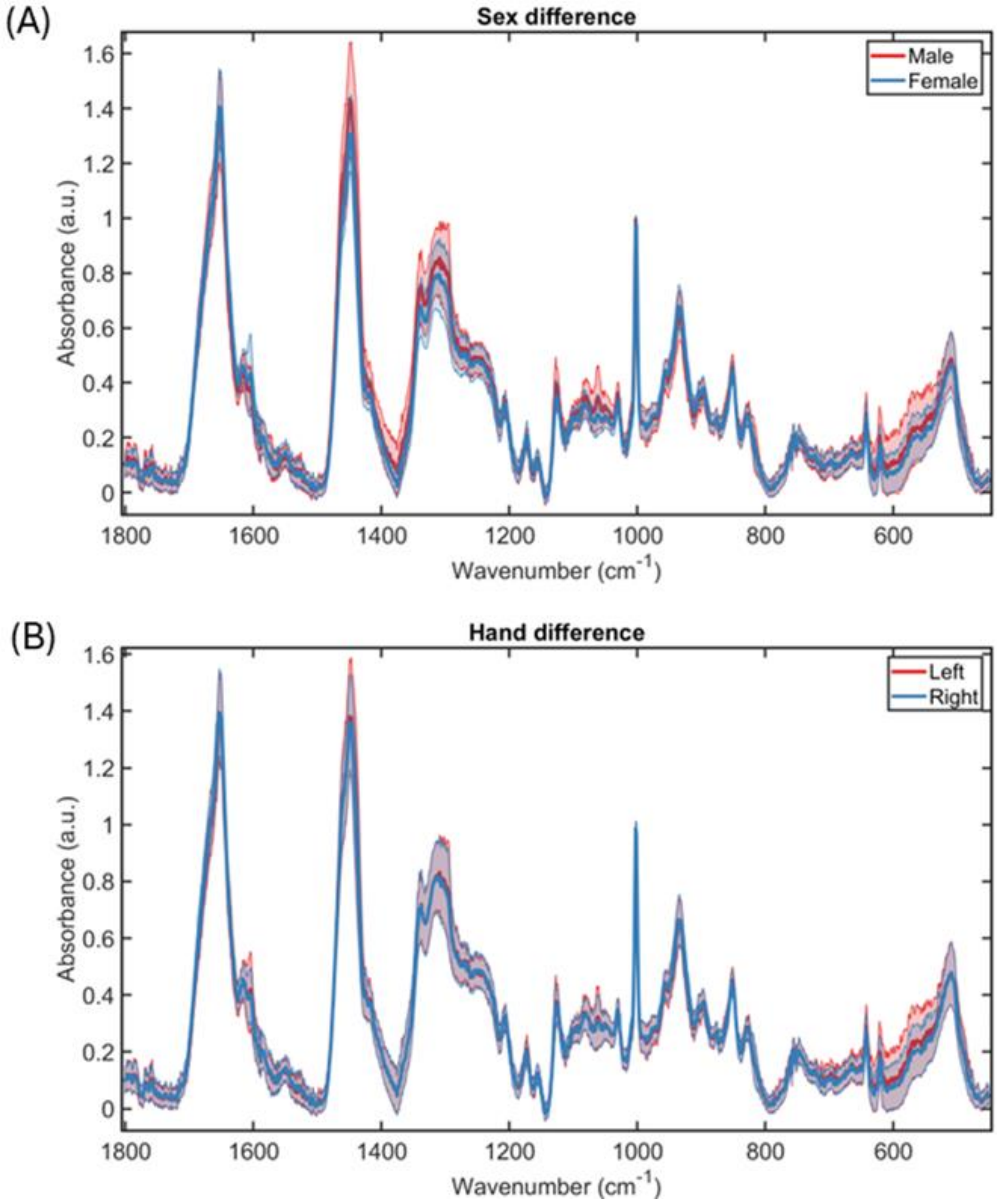

### LB3

### Evaluating the relationship between type 2 diabetes, popliteal artery circumference and osteoarthritis in the knee

Paul Rothwell[1,2], Nathan Jeffery[1], Alistair Bond[2], James Gallagher[1]

[1]University of Liverpool, Institute of Life Course and Medical Sciences, Liverpool, UK [2]University of Liverpool, Human Anatomy Resource Centre, Liverpool, UK

### Abstract

Introduction

Osteoarthritis (OA) is the most common condition to affect joints and a major cause of limitation and morbidity globally; affecting 528 million people. Diabetes is the most prevalent and rapidly increasing metabolic disorder in the world; in 2019 over 3.9 million people in the UK had diabetes with 90% of these having type 2 diabetes mellitus (T2DM). There is conflicting evidence suggesting that individuals with T2DM have an increased risk of OA.

Articular cartilage, whilst avascular, relies indirectly upon an adequate blood supply to the joint. If vessels become diseased, as is common in T2DM, it is postulated that this can result in poor joint health leading to the initiation or acceleration of OA.

Aims

This study aims to evaluate whether changes in blood vessel circumference, observed in T2DM, is related to the presence of OA in the knee joint.

Method

Using data from the Osteoarthritis Initiative (OAI) (https://nda.nih.gov/oai/), MRI knee scans have been used to measure popliteal artery luminal circumference. To account for the non-linear path taken by the artery, a 3D reconstruction was used to orientate slices in the correct plane for accurate measurement (Fig1 A, B). Average circumference is compared to Kellgren and Lawrence OA classification according to the OAI database.

MRIs of patients with a BMI <30 and T2DM were compared to non-diabetic controls. Age, sex and BMI have all been considered to rule out confounding results.

Results

Results (Fig1 C) show males with T2DM have a smaller lumen, but this does not relate to severity of OA. Females with T2DM have smaller lumen size and this is related to OA severity.

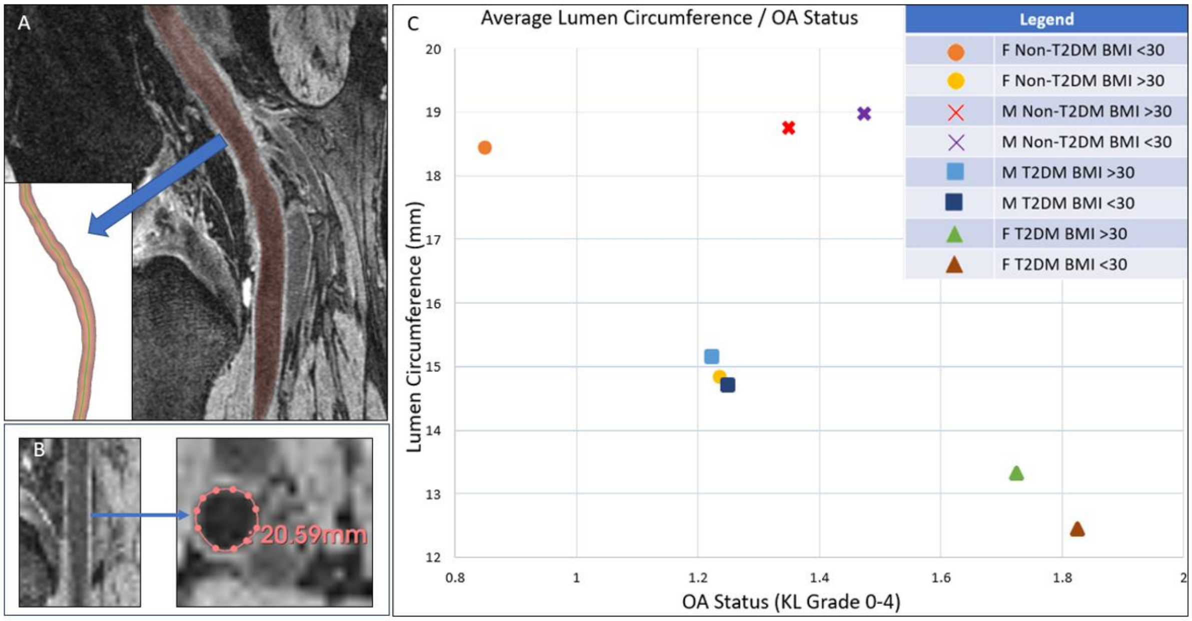

Fig 1. 3D Reconstruction of lumen used to orientate each MRI slice to centreline (A). Axial view of straightened volume used to measure lumen (B). Popliteal luminal circumference vs Kellgren Lawrence Grade of 160 patients (20 patients per cohort, left and right knee) (C).

The method used above will be validated using cadaveric knees of T2DM and non-T2DM. Knees will be scanned using the same MRI protocol in OAI study. The popliteal artery will then be dissected out to accurately measure the lumen.

### Conclusion

OA severity is related to lumen size in women with T2DM. However, these findings are not seen in men. This suggests that there may be a possible link between T2DM and OA severity in women and this could potentially be a target for therapeutic intervention. Further assessment from more patients will help to confirm this. With better understanding of this mechanism patients with T2DM can be advised to manage their condition better and be at less risk from poor joint health.

### LB4

### Should same day discharge be the standard in guided growth plates insertion

Vishal Dayal, Hussein Noureddine

Leicester Royal Infirmary, Leicester, UK

### Abstract

### Purpose

To assess if guided growth plate surgery can be performed as a day case to expedite patient rehabilitation, reduce hospital stay and achieve cost reductions.

### Methods

Data was collected retrospectively on all cases of guided growth plates (temporary hemiepiphysiodesis) performed over a 1-year period. The parameters examined included: number of plates inserted, anatomical location of plates, time spent in theatre, lead consultant, date of admission and length of hospital stay.

### Results

A total of twenty-seven cases were identified and evaluated of which only 10 cases (37%) were discharged on the same day. Most cases involved insertion of two plates and only 50% of these patients were discharged on the same day. Those who had three plates (20% SDD) or four plates (17% SDD) were more likely to stay overnight. And there was one outlier with one of the patients experiencing prolonged stay of six days due to their medical background

### Conclusion

Most surgeons will decide if a case is suitable to be a day case depending on patient factors, the number of plates, and the location of plates. Our data suggested that no single variable governs suitability for same day discharge, however prior planning , early engagement of physiotherapy, and set protocols developed in conjuction with the ward nursing staff generally enables the majority of said cases to be discharged on day case basis regardless of the number or location of the plates. This is in line with the published literature [1].

Our study prompted us to set a protocol for guided growth surgery working alongside the allied health professionals, aiming as a general rule to achieve same day discharge, but still accommodating patient variables including medical background. We aim to re-evaluate our outcomes after implementing the protocol in one year.

### LB5

### Selective venous sampling and radio frequency ablation in a patient with tumor induced osteomalacia

Manjunath P R[1], Praveen V P[2]

[1]Department of Endocrinology, Ramaiah Medical College, Bengaluru, India; [2]Department of Endocrinology, Amrita Institute of Medical Sciences, Cochin, India

### Abstract

Tumor-induced osteomalacia (TIO) is a rare paraneoplastic condition characterized by renal phosphate wasting due to excess fibroblast growth factor 23 (FGF23) leading to phosphaturia and hypophosphatemia. Presentation of this is usually as musculoskeletal pain and fractures. Biochemically patients have inappropriately low vitamin D levels with low phosphorous levels. Mesenchymal tumors of bone or soft tissue are the most common aetiologies causing this condition. Diagnosis of this disease is often challenging as the clinical course is slow and the tumors are usually small, but diagnosis is very important as it's a treatable condition with complete resolution of symptoms on treatment of the tumor, usually by surgical resection. The following case report describes a middle-aged male investigated for probable TIO.

Despite normal FGF23 levels on two occasions in the presence of severe disease, investigations including MRI and PET-CT showed a subcentimetric suspected lesion in the right greater trochanteric region. Subsequent investigation by selective venous sampling found high FGF23 levels in the right common femoral vein. The lesion was subsequently ablated using radiofrequency ablation (RFA), rapidly followed by dramatic improvements in the hypophosphatemia and symptoms.

This case report highlights the potential important role of selective venous sampling of FGF23 in patients with suspected TIO but with normal FGF23 level. RFA seems an effective alternative to surgery in vry ill patients or in those with lesions in surgically inoperable sites.
